# An Exploration of the Universal and Switchable RAFT-Mediated Synthesis of Poly(styrene-*alt*-maleic acid)-*b*-poly(*N*-vinylpyrrolidone) Block Copolymers

**DOI:** 10.1021/acs.macromol.4c02741

**Published:** 2025-01-13

**Authors:** Lauren E. Ball, Michael-Phillip Smith, Rueben Pfukwa, Bert Klumperman

**Affiliations:** Department of Chemistry and Polymer Science, University of Stellenbosch, Private Bag X1, Matieland 7602, South Africa

## Abstract

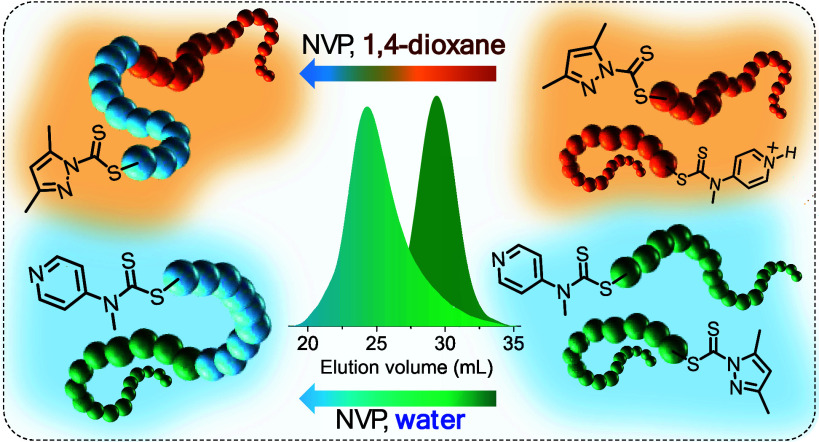

The synthesis of poly(styrene-*alt*-maleic anhydride) (SMAnh) and poly(4-*tert*-butylstyrene-*alt*-maleic anhydride) (*t*BuSMAnh) macro-RAFT agents was investigated using universal 3,5-dimethylpyrazole dithiocarbamate and stimuli-responsive *N*-(4-pyridinyl)-*N*-methyldithiocarbamate RAFT agents. SMAnh/*t*BuSMAnh macro-RAFT agents of targeted molecular weight and narrow molecular weight distribution could be synthesized with intentional variation of the terminal monomer unit, allowing for the assessment of two distinctive macro-R-groups. SMAnh macro-RAFT agents were utilized to mediate the thermally initiated polymerization of *N*-vinylpyrrolidone (NVP), yielding SMAnh-*b*-PVP, but with significant thermolysis and hydrolysis of dithiocarbamate ω-chain ends. Alternatively, the redox-initiated RAFT-mediated polymerization of NVP at ambient temperatures using hydrolyzed macro-RAFT agents, i.e., poly(styrene-*alt*-maleic acid) (SMA) and poly(4-*tert*-butylstyrene-*alt*-maleic acid) (*t*BuSMA), was explored. Double hydrophilic SMA-*b*-PVP and *t*BuSMA-*b*-PVP block copolymers could be synthesized but with significant broadening of the molecular weight distribution. This is a result of the formation of dead chains derived from the alkaline hydrolysis of macro-RAFT agents prepolymerization and hydrolysis of dithiocarbamate chain ends throughout the polymerization. The latter is exacerbated by the insertion of NVP at the ω-chain end, which was subsequently investigated via the kinetic analysis of the xanthate- and dithiocarbamate-mediated aqueous homopolymerization of NVP.

## Introduction

Amphiphilic block copolymers constituting a hydrophobic block and hydrophilic block undergo self-assembly upon application of the appropriate experimental parameters, e.g., a change in solvent composition, polymer concentration, or temperature. Double hydrophilic block copolymers (DHBCs), on the other hand, constitute two or more hydrophilic polymers of variable composition, and the amphiphilicity of the BCP can be amplified via introduction of a stimulus (pH, temperature, ionic strength, complexing molecules, etc.).^1,2^ This promotes self-assembly, giving DHBCs utility in applications such as crystal growth modification, nanoparticle fabrication, metal oxide particle stabilization, controlled drug delivery, gene transfection, cell and organelle mimics, etc.^2−6^ Poly(acrylic acid)-*block*-poly(ethylene glycol) (PAA-*b*-PEG) is a DHBC that has been used for the synthesis of hybrid polyion complexes (HPICs). Complexation of the acid groups of the PAA block to multivalent cations such as Gd^3+^ or Fe^3+^ or cationic drugs facilitates the self-assembly of PAA-*b*-PEG, effectively creating a chemical reservoir of the cation for application as core–shell catalysts, magnetic resonance imaging (MRI) contrasting agents, or drug delivery vehicles.^3,4,7^ While PAA-*b*-PEG is an effective DHBC in a variety of applications, there is increasing evidence within the biomedical field that repeated administration of PEGylated formulations can induce unexpected immune responses.^8,9^ Additionally, the preparation of PEG-based block copolymers lacks modularity, as PEG chain ends require multistep transformations to facilitate their chain extension via radical polymerization processes. Alternative DHBCs such as PAA-*block*-poly(*N*-vinylpyrrolidone) (PAA-*b*-PVP) have been developed and exhibit similar complexation capabilities compared to PAA-*b*-PEG, but with an improved ability to prevent antibody opsonization and inflammatory responses.^7,10,11^ An interesting alternative to PAA-*b*-PVP is poly(styrene-*alt*-maleic acid)-*block*-PVP (SMA-*b*-PVP), where the stimuli-responsive PAA block is replaced by a comparatively more amphiphilic SMA block, which has established significance in the biomedical field. SMA has been utilized for the synthesis of pH-sensitive cisplatin (CDDP)-SMA complexes for the treatment of various cancers, and amphiphilic block copolymers constituting SMA have been utilized for the encapsulation of hydrophobic anticancer drugs such as DOX and parthenolide.^12−14^ Due to its amphiphilic nature, SMA and its analogues have also been successfully employed in the solubilization of drug targets (membrane proteins) from their native lipid environment within the cell membrane into nanosized discoidal structures termed SMA-lipid particles (SMALPs).^15^

DHBCs are synthesized using controlled radical polymerization techniques such as reversible addition–fragmentation chain transfer (RAFT)-mediated polymerization or via conjugation of individual macromolecular building blocks by means of click chemistry.^1^ A well-controlled RAFT-mediated polymerization results in the synthesis of polymers with predictable molecular weight, narrow molecular weight distribution, minimal retardation of the polymerization kinetics, high end-group fidelity, and therefore access to complex macromolecular architectures. Control over the polymerization is facilitated by the appropriate selection of RAFT agent (with the general structure ZC(=S)SR) for the monomer type undergoing polymerization (“more activated” monomers (MAMs) vs “less activated’ monomers (LAMs)).^16^ A variety of poly(MAM)-*b*-poly(LAM) DHBCs comprising PVP as the poly(LAM) block have been reported.^11,17−20^ These synthetic protocols utilize either xanthate or dithioester RAFT agents, resulting in compromised RAFT agent activity for one of the blocks. Dithioesters are known to retard (and in some cases completely inhibit) the RAFT-mediated polymerization of NVP.^21^ Additionally, some protocols employ an unconventional block order.^17−19^ Generally, poly(LAM) propagating radicals are poor homolytic leaving groups compared to poly(MAM) propagating radicals; therefore, poly(MAM) macro-RAFT agents conventionally are synthesized first.^22^ To overcome the constraints in scope and utility of individual RAFT agent types, the range of RAFT agents is continually expanding, especially toward the development of universal RAFT agents. Dithiocarbamate (Z = NR’R”) RAFT agents are specifically attractive as their activity can be tuned through variation of the R’ and R” substituents.^22^ Dithiocarbamates of relevance to our study are the universal 3,5-dimethylpyrazole dithiocarbamates, which provide balanced activity for both LAMs and MAMs,^23,24^ and the switchable *N*-methyl-*N*-(4-pyridinium) dithiocarbamates,^25,26^ which can be switched from their low-activity unprotonated state (suitable for LAM polymerizations) to a high-activity protonated state (suitable for MAM polymerizations) via the stoichiometric addition of a strong acid.^25^

**Scheme 1:**
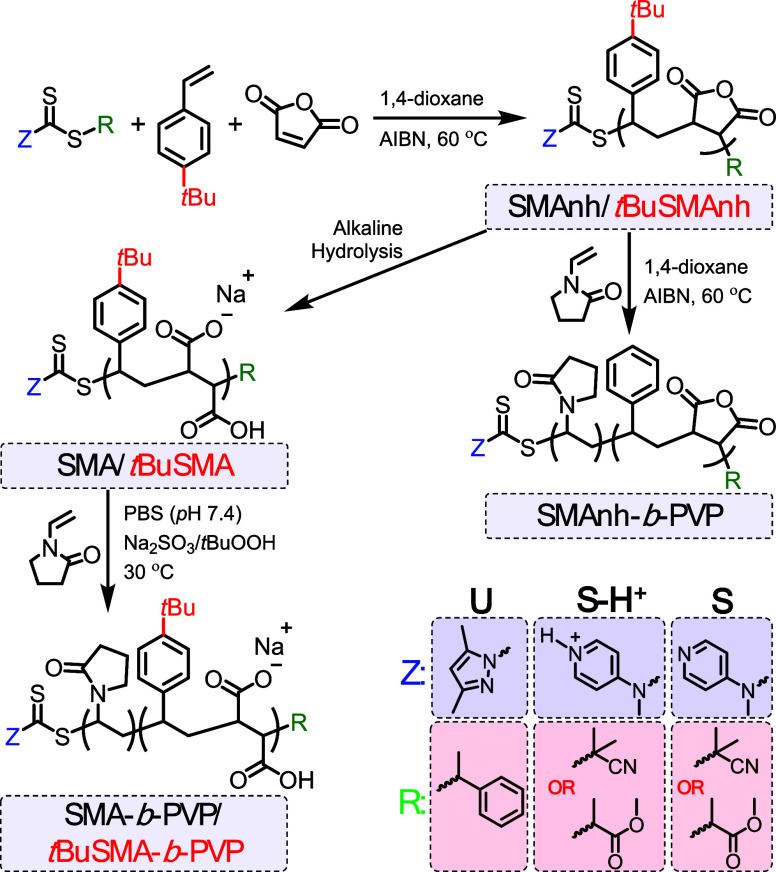
Synthesis of SMAnh/*t*BuSMAnh Macro-RAFT Agents Using Universal (**U**) or Switchable (**S-H**^**+**^ and **S**) RAFT Agents and Their Subsequent Chain Extension with PVP

In this investigation, we explore the synthesis of SMA-*b*-PVP and its more hydrophobic analogue *t*BuSMA-*b*-PVP via RAFT-mediated polymerization using universal and switchable dithiocarbamates. The general synthetic procedure followed is outlined in Scheme 1, where a universal/switchable SMAnh or *t*BuSMAnh (poly(MAM)) macro-RAFT agent is synthesized first. As this investigation serves as the first example of the dithiocarbamate-mediated synthesis of SMAnh/*t*BuSMAnh, an exploration of the RAFT-mediated polymerization kinetics is undertaken. The thermally initiated chain extension of SMAnh/*t*BuSMAnh macro-RAFT with PVP in organic media is presented. Alternatively, the macro-RAFT agents undergo alkaline hydrolysis to yield water-soluble SMA/*t*BuSMA macro-RAFT agents, which are used to mediate the aqueous redox initiated polymerization of NVP. The RAFT polymerization of NVP is historically associated with many synthetic challenges.^27,28^ The exploration of limitations associated with the xanthate-mediated polymerization of NVP is well-reported, but very little information is available regarding whether dithiocarbamate-mediated polymerization of NVP suffers the same limitations. Thus, the homopolymerization of NVP in water using xanthate or universal or switchable dithiocarbamates is also presented.

## Experimental Methods

### Materials

Styrene (STY, Merck, ≥99%, stabilized with *tert*-butylcatechol) and 4-*tert*-butylstyrene (*t*BuSTY, Merck, 93%, stabilized with *tert*-butylcatechol) were eluted from an aluminum oxide (Merck, activated basic, Brockmann I) column prior to use. Maleic anhydride (MAnh, Merck, 99%) and 1,3,5-trioxane (Merck, 99%) were recrystallized from distilled chloroform, filtered, and dried under a vacuum or alternatively sublimed as needed. Azobis(isobutyronitrile) (AIBN, Merck, 98%) was recrystallized from anhydrous methanol, filtered, and dried under a vacuum. 4-*N*,*N*-Dimethylaminopyridine (DMAP, Merck, ≥99%) was recrystallized from dry toluene, filtered, and dried under a vacuum. THF was predried over KOH pellets, filtered, and distilled over sodium/benzophenone. A PBS solution (pH = 7.4) constituting 0.01 M phosphate buffer, 0.0027 M potassium chloride, and 0.137 M sodium chloride was prepared via dissolution of one PBS tablet (Merck) in 200 mL deionized (DI) H_2_O. KOH (Merck, 90%, flakes), CS_2_ (Kimix, 99%), 3,5-dimethylpyrazole (Merck, 99%), bromo acetonitrile (Merck, 97%), 1-bromoethylbenzene (Merck, 97%), *N*,*N*′-dimethyl *N*,*N*′-di(4-pyridinyl)thiuram disulfide (Merck), methyl 2-(methyl(pyridin-4-yl)carbamothioylthio)propanoate (Merck, 95%), trifluoromethanesulfonic acid (TfOH) (Merck, 98%), NVP (Merck, ≥99%, stabilized by NaOH), Na_2_SO_3_ (Merck, ≥98%), *t*BuOOH (Merck, 70 wt % in H_2_O), (trimethylsilyl)diazomethane (Merck, 2.0 M in hexane), Na_2_CO_3_ (Merck, ≥98%), 1,4-dioxane (Merck, anhydrous, 99.8%), acetone (Merck, ≥99.5%), *N*,*N*-dimethylformamide (DMF, Merck, anhydrous, 99.8%), NaOH (Merck, pellets for analysis), and 3500 MWCO SnakeSkin dialysis tubing (Thermo Fisher Scientific) were used as received.

### Synthesis of SMAnh/*t*BuSMAnh Macro-RAFT Agents

RAFT agents used in this study were synthesized according to previously reported procedures (see the Supporting Information).^23,25^ Considering **U**SMAnh (Table 1, entry 1) as a representative copolymerization, 1-phenylethyl 3,5-dimethyl-1*H*-pyrazole-1-carbodithioate (**U**) (0.23 g, 0.82 mmol), STY (2.1 g, 20 mmol), MAnh (2.0 g, 20 mmol), AIBN (26 mg, 0.16 mmol), 1,3,5-trioxane (34 mg, 0.38 mmol), and 1,4-dioxane (14 mL) were added to a three-neck round-bottom flask fitted with a magnetic stirrer bar, rubber septum, and bubbler. The solution was sparged with dry argon for 45 min, and the flask was subsequently immersed in an oil bath preheated to 60 °C. After 21 h, the polymerization was quenched via exposure to atmospheric oxygen, the solution was cooled and diluted with acetone (5–8 mL), and the copolymer was subsequently isolated via precipitation in pentane (160 mL) and centrifugation (4500 rpm, 3 min). This was repeated twice more followed by drying of the SMAnh pellets in a vacuum oven at ambient temperature for 24 h. For copolymerizations that employ an excess of either comonomer, the same protocol was followed with the inclusion of the excess in the initial comonomer feed or alternatively added at 8 h. For the latter, a solution of the comonomer in 1,4-dioxane (30 w/v%) was prepared (with an additional 0.2 equiv AIBN relative to the RAFT agent for copolymerizations using excess STY/*t*BuSTY) and sparged with dry argon for 0.5 h prior to addition to the polymerization mixture with a degassed syringe. For kinetic copolymerization experiments, aliquots (∼0.5 mL) of the polymerization mixture were withdrawn with a degassed syringe at specified time intervals, where 0.1 mL was diluted in (CD_3_)_2_CO for ^1^H NMR spectroscopic analysis, and the rest were dried under a vacuum and redissolved in THF (5% AcOH) at 2 mg/mL for SEC analysis. All SMAnh/*t*BuSMAnh copolymers were characterized via ^1^H NMR, ATR-FTIR, UV–vis spectroscopy, and SEC.

**Table 1:** Monomer Conversion and Molecular Weight Analysis for **U**/(**S-H**^**+**^)-Mediated Synthesis of SMAnh/*t*BuSMAnh

entry	sample code^a^	reagent ratio^b^	α^STY^ (%), α^MAnh^ (%)^c^	*M*_n_^theo^ (g/mol)^d^	*M*_n_^SEC^ (g/mol)^e^	*Đ*^e^	*k*_p_^app^ (h^–1^)^f^
**1**	**U**SMAnh	1:25:25:0.2	100, 97	5300	5300	1.15	0.27
**2**	**U***t*BuSMAnh*S1*	1:50(+15):51:0.2	84, 100	14,000	17,800	1.33	0.60
**3**	**U***t*BuSMAnh*S2*	1:29:20:0.2	83, 100	6100	6600	1.24	0.29
**4**	**(S-H**^**+**^**)***t*BuSMAnh*S*	1:50(+15):51:0.2	85, 100	14,100	17,100	1.31	0.49
**5**	**(S-H**^**+**^**)**SMAnh*S*	1:25(+7):25:0.2	87, 100	5600	6800	1.39	
**6**	**U**SMAnh*M1*	1:25:25(+7):0.2	100, 86	5600	1800*	1.48*	0.32
**7**	**U**SMAnh*M2*	1:25:32:0.2	100, 77	5300	1700*	1.46*	0.35
**8**	**U**SMAnh*M3*	1:25:32:0.2	100, 81	5400	1800*	1.48*	
**9**	**U**SMAnh*S*_*3k*_	1:18:15:0.1	96, 100	3500	3700	1.13	
**10**	**U**SMAnh*M*_*3k*_	1:15:19:0.1	100, 79	3300	2700	1.22	

a **U** or **S-H**^**+**^ indicates the use of 1-phenylethyl 3,5-dimethyl-1*H*-pyrazole-1-carbodithioate or 2-cyanopropan-2-yl methyl(pyridin-4-yl) carbamodithioate as RAFT agent, respectively. *S* or *M* specifies the terminal monomer unit as STY or MAnh, respectively.

b [RAFT]/[STY]/[MAnh]/[AIBN], where values included in brackets indicate that an additional amount of the monomer was added after 8 h of polymerization.

c Monomer conversions determined via ^1^H NMR spectroscopy using 1,3,5-trioxane as the internal standard and eq S1.

d Calculated using eq S2.

e Determined via SEC analysis using THF (5% AcOH) as eluent and PS calibration standards. Samples marked with an asterisk were analyzed using DMF (0.05 M LiBr, 40 °C) and PMMA calibration standards.

f Calculated using the slope of ln([*M*]_0_/[*M*]_*t*_) vs time curves (Figure 1 and Figure S2).

### Hydrolysis/Deprotonation of SMAnh/*t*BuSMAnh

In a typical SMAnh/*t*BuSMAnh alkaline hydrolysis protocol (using (**S-H**^**+**^)SMAnh*S*, Table 1 entry 5, as an exemplary experiment), the copolymer (13 g, 2.3 mmol) was dissolved in acetone (∼20 mL) and added to a solution of Na_2_CO_3_ (10 g, 97 mmol, 1.7 equiv relative to MAnh units) in DI water (125 mL), facilitating the deprotonation of the Z-group and precipitation of the copolymer. The suspension was stirred at 40 °C for up to 24 h or until the precipitate had fully dissolved in the predominantly aqueous solution due to significant hydrolysis of MAnh units to corresponding maleic acid (MAc) units. The solution was added to 3500 MWCO dialysis tubing and dialyzed against DI water for 3 days with water changes occurring three to four times daily until the dialysis water maintained a neutral pH. Alternatively, hydrolyzed copolymers were dialyzed via tangential flow filtration (TFF) until the collected filtrate maintained a consistent pH. For comparatively more hydrophobic*t*BuSMAnh copolymers, a similar hydrolysis protocol was employed, but suspensions were prepared at 3 w/v % instead of 9 w/v % (SMAnh suspensions). Dialyzed copolymers were lyophilized and characterized via ^1^H NMR, ATR-FTIR, and UV–vis spectroscopy.

### Synthesis of SMAnh-*b*-PVP

Thermally initiated block copolymerizations of NVP using SMAnh macro-RAFT agents were all conducted in freshly distilled 1,4-dioxane and NVP. Considering the **U**SMAnh*M*-*b*-PVP (Table 2, entry 6) as a typical block copolymerization, **U**SMAnh*M* was synthesized according to the general procedure described above but using freshly sublimed MAnh (instead of recrystallized MAnh to limit contamination with MAc) and in a Schlenk flask fitted with a magnetic stirrer bar and rubber septum. The **U**SMAnh*M* polymerization mixture was deoxygenated via six freeze–pump–thaw cycles, backfilled with dry argon, and immersed in a preheated oil bath (60 °C) for 18 h. The flask remained sealed while the solution cooled, and an aliquot was withdrawn with a degassed syringe for ^1^H NMR spectroscopic analysis. To the remaining solution of **U**SMAnh*M* (theoretical mass = 2.1 g, 0.39 mmol), NVP (2.3 g, 21 mmol), AIBN (11 mg, 67 μmol), and 1,4-dioxane (7.6 mL) were added. The solution was deoxygenated via six freeze–pump–thaw cycles, backfilled with dry argon, and immersed in a preheated oil bath (60 °C) for 24 h. The solution was cooled and diluted with dichloromethane (∼5 mL), and the block copolymer was precipitated in diethyl ether (80 mL) and centrifuged (4500 rpm, 3 min). This precipitation procedure was repeated twice, and the resulting pellets were dried under a vacuum at ambient temperature for 24 h. **U**SMAnh*M*-*b*-PVP was characterized via ^1^H NMR, DOSY NMR spectroscopy, and SEC.

**Table 2:** Monomer Conversions and Molecular Weight Analysis for **U**SMAnh*S* Macro-RAFT Agents and the Corresponding **U**SMAnh*S*-*b*-PVP Block Copolymers

entry	sample^a^	reagent ratio^b^	α^STY^ (%), α^MAnh^ (%^c^	α^NVP^ (%)^c^	*M*_n_^theo^ (g/mol)^d^	*M*_n_^SEC^ (g/mol)^e^	*Đ*^e^
**1**	**U**SMAnh*S*	1:32:25:0.1	91, 100		5800	5600	1.14
**2**	**U**SMAnh*S*-*b*-PVP	1:60:0.5		96			
**3**	**U**SMAnhS*	1:31:26:0.2	92, 98		5700		
**4**	**U**SMAnh*S*-*b*-PVP*	1:60:0.2	65, 100	4	6200		
**5**	**U**SMAnhM*	1:25:25:0.1	100, 91		5300	3700	1.40
**6**	**U**SMAnh*M*-*b*-PVP*	1:60:0.2	-, 100	35	7700	4700	1.69

a **U** indicates that the universal RAFT agent was used. *S*/*M* indicates either STY or MAnh adjacent to the thiocarbonylthio group. Samples labeled with an asterisk indicate that the chain extension step takes place *in situ.*

b [RAFT]/[STY]/[MAnh]/[AIBN] for SMAnh samples and [macro-RAFT]/[NVP]/[AIBN] for SMAnh-*b*-PVP samples.

c Determined via ^1^H NMR spectroscopy using 1,3,5-trioxane as the internal standard and eq S1 (for SMAnh samples) and eq S3 (for SMAnh-*b*-PVP samples).

d Calculated using eq S2 (for SMAnh samples) and eq S4 (for SMAnh-*b*-PVP samples).

e Determined via SEC analysis using THF (5% AcOH) as eluent and PS calibration standards (entry 1). Alternatively, DMF (2 mM LiBr, 60 °C) and SMAnh calibration standards were utilized.

### Synthesis of SMA-*b*-PVP

All experiments using SMA/*t*BuSMA macro-RAFT agents for the aqueous RAFT-mediated polymerization of NVP were conducted in PBS (at 10 w/v%) using redox initiation. Considering **U***t*BuSMAS_25_-*b*-PVP_34_ (Table 5, entry 2) as a typical block copolymerization, **U***t*BuSMAS_25_ (3.3 g, 0.46 mmol), NVP (2.3 g, 21 mmol), *t*BuOOH (21 mg, 0.23 mmol), Na_2_SO_3_ (30 mg, 0.24 mmol), DMF (0.10 g, 1.4 mmol), and PBS (*p*H 7.4, 29 mL) were added to a Schlenk flask fitted with a magnetic stirrer bar and rubber septum. If the *t*BuSMA copolymer increased the *p*H of the solution significantly, it was titrated with aliquots of HCl (1.0 M) until a pH of∼7.6 was achieved. The polymerization mixture was deoxygenated via six freeze–pump–thaw cycles and backfilled with dry argon, and the flask was immersed in a preheated oil bath (30 °C) for 24 h. Sparging an aqueous solution of *t*BuSMA with argon facilitates significant foaming, and therefore, freeze–pump–thaw sparging protocols are preferred. For block copolymerizations using SMA macro-RAFT agents, the freeze–pump–thaw protocol is unnecessary (as this copolymer has a lower surfactant activity than *t*BuSMA), and the solution can be sparged by using argon gas. Block copolymers were purified via dialysis (using either a 3500 MWCO dialysis tubing or a TFF protocol) and lyophilized prior to characterization via SEC (after methylation), ^1^H NMR, DOSY NMR, ATR-FTIR, and UV–vis spectroscopy.

### Synthesis of PVP

In a typical aqueous RAFT-mediated homopolymerization of NVP (e.g., **S**PVP, Table4, entry 3), 2-cyanopropan-2-yl methyl(pyridin-4-yl) carbamodithioate (**S**) (63 mg, 0.25 mmol), NVP (5.0 g, 45 mmol), *t*BuOOH (11 mg, 0.13 mmol), DMF (0.1 g, 1.4 mmol), and PBS (*p*H 7.4, 16 mL) were added to a three-neck round-bottom flask fitted with a magnetic stirrer bar, rubber septum, and bubbler. The solution was sparged with argon gas for 45 min, and a solution of Na_2_SO_3_ (16 mg, 0.12 mmol) in PBS (1.0 mL, sparged for 15 min with argon gas) was added using a degassed syringe prior to immersion in a preheated oil bath (30 °C) for ∼24 h. For kinetic polymerizations, aliquots were withdrawn from the polymerization using a degassed syringe. 0.1 mL reaction mixture was diluted in (CD_3_)_2_SO for NVP conversion determination. Circa 0.6 mL reaction mixture was lyophilized and subsequently dissolved in (CD_3_)_2_SO or in DMF (2 mM LiBr) for ^1^H NMR spectroscopy and SEC analysis, respectively.

### Characterization

NMR spectroscopic analyses were carried out using a 400 MHz Agilent NMR Spectrometer or a 400 MHz/600 MHz Bruker Ascend Spectrometer, specified per sample. Samples were dissolved in (CD_3_)_2_CO (Merck, Magni-Solv, 99.9%), (CD_3_)_2_SO (Merck, Magni-Solv, 99.9%), or D_2_O (Merck, Magni-Solv, 99.9%) prior to analysis, specified per sample. All DOSY NMR spectroscopy measurements were performed at 298 K on an Agilent Inova 400 NMR spectrometer operating at 400 MHz and equipped with a two-channel multinuclear *z*-gradient inverse probe head capable of producing gradients in the *z* direction with a calibrated gradient strength of 0.00213 G/cm/DAC. The DOSY spectra were acquired with the Dbppste_cc (convection compensation) pulse program from the VnmrJ 4.2 topspin software. All spectra were recorded with 16–32 K time domain data points in the t2 dimension and 30 t1 increments. The gradient strength was logarithmically incremented in 30 steps from 5 to 100% of the maximum gradient strength. All measurements were performed with a diffusion delay of around 250 ms and a gradient pulse length of 2 ms. Both of these parameters were adjusted slightly to ensure a signal attenuation of more than 85%. The diffusion dimension of the 2D DOSY spectra was processed by a licensed Mnova 12 software package.

Size exclusion chromatography was conducted using three different systems, specified per sample. The first protocol, referred to as DMF (0.05 M LiBr, 40 °C) in the main text, employed LiBr stabilized DMF (Merck, Chromosolv Plus, for HPLC, ≥99.9%), with samples dissolved at 2 mg/mL prior to analysis. The samples were filtered using 0.45 μm PTFE filters (Sartorius) prior to analysis with an Agilent 1260 HPLC instrument fitted with a quaternary pump, thermostated column compartment set at 40 °C, an autosampler, a differential refractometer set at 40 °C, and a diode array UV detector set at 290 and 320 nm. Columns utilized were PSS 10 μm GRAM columns (guard column of 50 × 8 mm inner diameter and two linear M analytical columns of 300 × 8 mm i.d.). The flow rate during analysis was 1.0 mL/min, and the injection volume per sample was 100 μL. The system was calibrated using low *Đ* PMMA calibration standards with a molar mass range of 800–2,200,000 g/mol. The second protocol, referred to as DMF (2 mM LiBr, 60 °C) in the main text, utilized the same setup above with the thermostated column compartment and differential refractometer alternatively set at 60 °C and 50 °C, respectively. Columns utilized were the Agilent PLgel Mixed-C (5 μm) guard column (50 × 7.5 mm i.d.) and two analytical columns (300 × 7.5 mm i.d.). PMMA calibration standards (800–2,200,000 g/mol) as well as RAFT-synthesized SMAnh calibration standards (600–90 000 g/mol) were utilized. The third protocol, termed THF (5% AcOH) in the main text, employed THF (5% v/v AcOH with 0.125% BHT, Merck, for HPLC, ≥99.9%) with samples dissolved at 2 mg/mL and filtered using 0.45 μm RC filters (Sartorius) prior to analysis. The analysis was performed on an Agilent 1260 HPLC instrument fitted with a quaternary pump, a column compartment thermostated at 30 °C, a differential refractometer set at 30 °C, and a diode array UV detector set at 254 and 320 nm. The columns utilized were two Agilent Technologies PLgel 5 Mixed-C columns (300 × 7.5 mm inner diameter) and a PLgel 5 Guard column (50 × 7.5 mm i.d.). The flow rate during analysis was 1.0 mL/min, and the injection volume per sample was 100 μL. The system was calibrated using low *Đ* PS calibration standards with a molar mass range of 580–2.0 × 10^6^ g/mol.

Attenuated total reflectance infrared (ATR-FTIR) spectroscopy was performed using a Thermo Scientific Nicolet iS10 Smart iTR using 128 scans over the wavelength range of 600–4000 cm^–1^, with a background spectrum (64 scans) obtained prior to each sample analyzed.

UV–vis spectroscopic analyses were conducted using a Shimadzu UV-1800 spectrophotometer with a double beam (deuterium lamp and tungsten-halogen lamp) and a silicon photodiode detector. Wavelength accuracy was up to ±0.1 nm, wavelength reproducibility was ±0.1 nm, and absorbance range was −4 to 4. Samples were analyzed within a wavelength range of 190–1100 nm in solvents and at concentrations specified per sample.

Tangential flow filtration was utilized for dialysis of samples using a PALL Minimate EVO system fitted with a peristaltic pump, two pressure gauges, sample reservoir, a magnetic stirrer plate, and a Minimate TFF capsule (Omega 1K membrane). Purifications were run using a pump flow rate of 40 mL/min and a transmembrane pressure of 2 bar until a desired amount of filtrate had been collected or the pH of the filtrate reached a desirable value.

Dynamic light scattering analyses for assessment of the polymer aggregates within polymerization mixtures were conducted using a ZetaSizer 1000 HSa (Malvern Instruments, Malvern) fitted with a 4 mW He–Ne laser operating at a wavelength of 633 nm and a scattering angle of 90°. Crude polymerization mixtures (1 mL) were analyzed, and then 10 μL of the crude mixture was withdrawn and dispersed in 1 mL of PBS (pH = 7.4) buffer and reanalyzed. Analyses were conducted using ZetaSizer Software 7.11, and the data were processed using OriginPro 9.0 software.

## Results and Discussion

### Synthesis of SMAnh/*t*BuSMAnh Macro-CTAs

STY and MAnh (MAMs) are generally copolymerized by using dithiobenzoates and trithiocarbonates. To the best of our knowledge, there are no reports of their dithiocarbamate-mediated RAFT copolymerization reported in the literature.^29−32^ SMAnh readily undergoes postpolymerization modifications via the MAnh units along the backbone, allowing for tuning of chemical functionality and amphiphilicity. Modification of the MAnh units is achieved using nucleophiles such as primary amines. These reagents, however, can also transform the RAFT thiocarbonylthio end groups, making this approach incompatible where the modified copolymer must be used as a macro-RAFT agent. Alternatively, the amphiphilicity of the copolymer can be adjusted by using analogues of the styrene comonomer, for example, alkylated styrene derivatives such as *t*BuSTY.^33^ In this way, the amphiphilicity of the stimuli responsive block can be modulated to afford DHBCs with a tunable solution behavior. The use of universal or switchable dithiocarbamates for the synthesis of SMAnh-type macro-RAFT agents additionally provides the opportunity to synthesize a wide variety of poly(MAM)-*b*-poly(MAM) and poly(MAM)-*b*-poly(LAM) copolymers; thus, our investigation has great potential to enrich the synthetic toolbox for the synthesis of highly functional block copolymers.

The RAFT-mediated copolymerization of STY/*t*BuSTY with MAnh was investigated using two different RAFT agents, the “universal” 1-phenylethyl 3,5-dimethyl-1*H*-pyrazole-1-carbodithioate (indicated with **U** for each copolymer) and the “switchable” 2-cyanopropan-2-yl methyl(pyridin-4-yl) carbamodithioate (indicated with **S–H**^**+**^ for each protonated copolymer and **S** for deprotonated copolymers), where variations in the R-group employed are stipulated per sample. All SMAnh/*t*BuSMAnh copolymerizations were performed in 1,4-dioxane (30 w/v%) at 60 °C using AIBN as the radical source and are summarized in Table 1. Generally, a [RAFT]/[STY/*t*BuSTY]/[MAnh]/[AIBN] ratio of 1:25:25:0.2 was applied, where exceptions are specified per experiment.

**U**SMAnh was synthesized using an equimolar comonomer feed (*f*_0_^STY/MAnh^ = 0.5) (Table 1, entry 1) and kinetic samples withdrawn periodically, where comonomer conversion was determined via ^1^H NMR spectroscopy and the molecular weight distribution was characterized via SEC. Quantitative monomer conversion was achieved with equimolar consumption of STY and MAnh at any given point during the copolymerization, characteristic of the strong alternating tendency of this copolymerization. The semilogarithmic plot for **U**SMAnh (Figure 1A) indicates a linear evolution of ln([*M*]_0_/[*M*]_*t*_) with time for the first 8 h of polymerization, indicating that this copolymerization follows pseudo-first order kinetics, after which point a deviation from linearity was observed due to depletion of the comonomers. The apparent propagation rate (*k*_p_^app^) was obtained from the slope of the ln([*M*]_0_/[*M*]_*t*_) vs time curves reported in Table 1 for each copolymerization. *M*_n_^theo^, *M*_n_^SEC^, and *Đ* were plotted as a function of the total comonomer conversion (Figure 1B). The evolution of *M*_n_^SEC^ with increasing monomer conversion was linear, and a decrease in *Đ* (with *Đ* = 1.15 at 24 h) with increasing monomer conversion was observed, both indications of a well-controlled RAFT-mediated copolymerization. *M*_n_^theo^ and *M*_n_^SEC^ correlated well throughout the polymerization, suggesting the complete conversion of the initial universal RAFT agent into a macro-RAFT agent.

**Figure 1:**
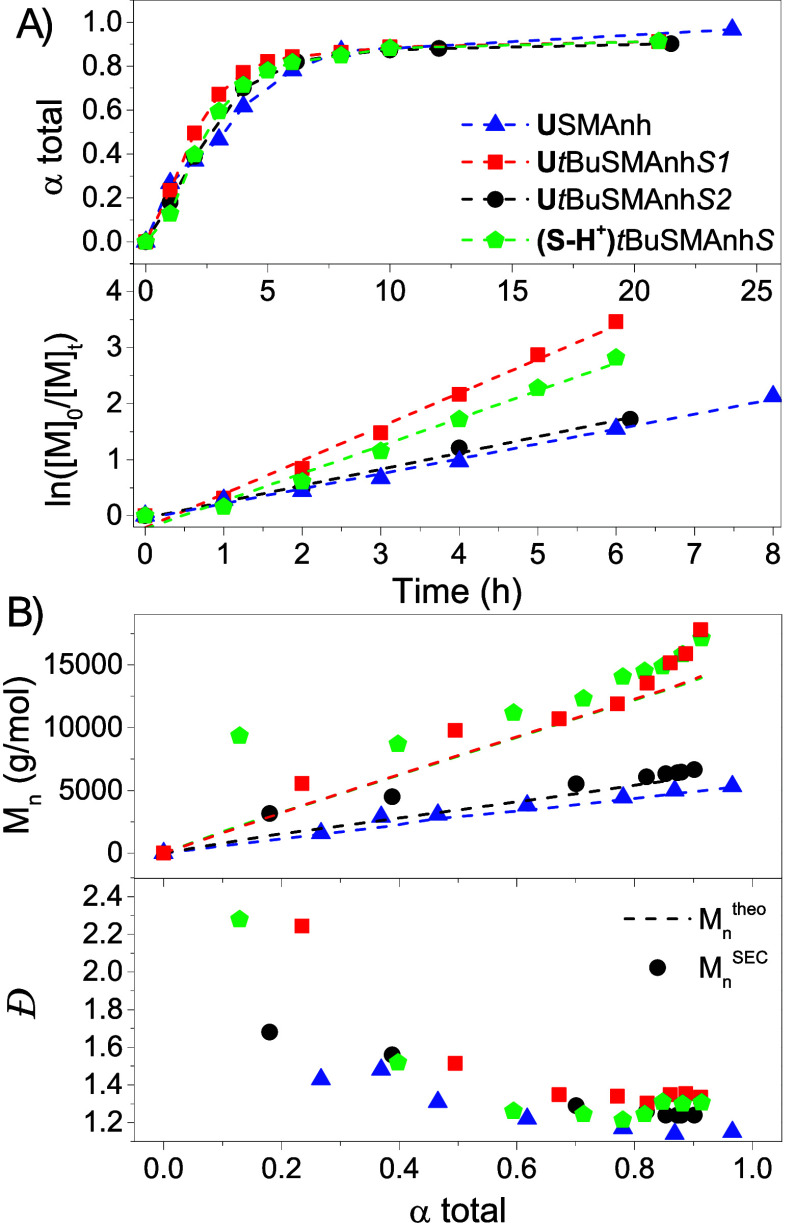
(A) RAFT-mediated polymerization kinetic analysis for the synthesis of SMAnh and *t*BuSMAnh using universal and switchable RAFT agents. Total monomer conversion (STY/*t*BuSTY + MAnh) vs time curves and corresponding semilogarithmic plot determined via ^1^H NMR spectroscopy. The blue data represent **U**SMAnh (triangles) and have equimolar amounts of MAnh/STY in the initial comonomer feed (*f*_0_^STY^ = 0.5); the black data represent **U***t*BuSMAnh*S2* (circles) correspond to *f*_0_^STY^ = 0.6; the red data represent **U***t*BuSMAnh*S1* (squares) with a *f*_0_^STY^ = 0.5 and an additional 0.3 equiv STY added at 8 h; and the green data represent **(S-H**^**+**^**)***t*BuSMAnh*S* (pentagons) with *f*_0_^STY^ = 0.5 and an additional 0.3 equiv STY added at 8 h. (B) *M*_n_^theo^ (dotted lines), *M*_n_^SEC^ (symbols), and *Đ* vs total monomer conversion curves following a similar color code as outlined above. Additional information regarding these polymerizations is provided in Table 1.

The macro-R-group of the **U**SMAnh/**S**SMAnh macro-RAFT agents has potentially different efficiencies as homolytic leaving groups and reinitiating radicals during the RAFT-mediated polymerization of NVP depending on whether the terminal monomer unit adjacent to the thiocarbonylthio moiety is STY or MAnh. To investigate this in more detail, either an excess of one comonomer is added in the initial comonomer feed or the addition occurs at the point where near-quantitative monomer conversion is reached. As the SMAnh RAFT-mediated copolymerization has a strong alternating tendency, *f*_0_^STY/MAnh^ ≠ 0.5 does not significantly affect the alternating character of the copolymerization until one of the comonomers is fully consumed, at which point a short polystyrene block may form (if *f*_0_^STY^ > 0.5) or a MAnh unit is inserted at the ω-chain end with no further polymerization taking place (if *f*_0_^STY^ < 0.5).

The universal RAFT-mediated copolymerization of *t*BuSTY and MAnh was conducted with *f*_0_^*t*BuSTY^ = 0.5 (Table 1, entry 2), and kinetic samples were withdrawn periodically throughout the copolymerization (Figure 1A, **U***t*BuSMAnh*S1*). Quantitative comonomer conversions were reached after ∼8 h, with the semilogarithmic plot indicating pseudo-first-order kinetics for the first 6 h of polymerization. The *tert*-butyl substituent on *t*BuSTY creates an electron-rich vinyl bond, resulting in a better electron-donor comonomer (compared to STY), for the *t*BuSTY-MAnh donor–acceptor pair. This yields a copolymerization with *k*_p_^app^ = 0.60 h^–1^, which is double that observed for the **U**SMAnh copolymerization (*k*_p_^app^ = 0.29 h^–1^). For the first 6 h of polymerization (α^total^ = ∼80%), the evolution of *M*_n_^SEC^ with increasing monomer conversion is mostly linear with a decrease in *Đ* observed (*Đ*_82% (6 h)_ = 1.30), indicative of a well-controlled RAFT-mediated polymerization. At 8 h, a solution with an additional 0.3 equiv of *t*BuSTY (relative to MAnh) and AIBN was added to the polymerization, and after an additional 13 h, an average of ∼3 *t*BuSTY units was inserted at the ω-chain end (calculated using monomer conversion). Above α^total^ = 82%, deviations of *M*_n_^SEC^ from *M*_n_^theo^ were observed, characterized by the appearance of a high-molecular-weight shoulder causing slight broadening of the molecular weight distribution (Figure S1). This could be due to the prevalence of termination side-reactions at higher monomer conversion (exacerbated by the added AIBN at 8 h). Alternatively, the universal RAFT-mediated copolymerization of *t*BuSTY and MAnh was conducted with *f*_0_^*t*BuSTY^ = 0.6 (Table 1, entry 3), resulting in a significant reduction in the *k*_p_^app^ (0.29 h^–1^, Figure 1A, **U***t*BuSMAnh*S2*), a phenomenon that has been observed for SMAnh copolymerizations with *f*_0_^STY^ > 0.5.^34^**U***t*BuSMAnh*S2*, similarly to **U***t*BuSMAnh*S1*, exhibited some hybrid behavior, as *M*_n_^SEC^ was larger than *M*_n_^theo^ at lower conversions but coincided with the calculated *M*_n_^theo^ at higher monomer conversions. This was not observed for **U**SMAnh, which might suggest that the initialization of the *t*BuSMAnh copolymerization with this universal RAFT agent is not as selective as that of the SMAnh copolymerization. Despite this, **U***t*BuSMAnh*S2* exhibited a linear increase in *M*_n_^SEC^ and a decrease in *Đ* with increasing monomer conversion, with the absence of the high-molecular-weight shoulder at higher monomer conversions (Figure S1).

Similar experimental parameters were used for the switchable RAFT-mediated copolymerization of *t*BuSTY and MAnh (Table 1, entry 4), with the inclusion of a stoichiometric amount of trifluoromethanesulfonic acid (TfOH) to facilitate the protonation of the pyridinyl group. The synthesis of **(S-H**^**+**^**)***t*BuSMAnh*S* was conducted with *f*_0_^*t*BuSTY^ = 0.5, with an additional 0.3 equiv *t*BuSTY (relative to MAnh) and AIBN added to the polymerization at 8 h. A comparison of the ln([*M*]_0_/[*M*]_*t*_) vs time curves for **(S-H**^**+**^**)***t*BuSMAnh*S* and **U***t*BuSMAnh*S1*, which are similar copolymerizations in every aspect apart from the RAFT agent used, indicates that the switchable RAFT agent causes slight retardation of the copolymerization kinetics. Furthermore, **(S-H**^**+**^**)***t*BuSMAnh*S* exhibits a more pronounced hybrid behavior at low monomer conversions compared to that of **U***t*BuSMAnh*S1*. At α^total^ > 40%, the evolution of *M*_n_^SEC^ with monomer conversion is mostly linear, with a corresponding decrease in *Đ*, which suggests that some control over the copolymerization is obtained despite the observed hybrid behavior.

Universal/switchable RAFT-mediated copolymerizations with an initial *f*_0_^MAnh^ = 0.5 and added 0.3 equiv MAnh at 8 h (Table 1, entry 6) or, alternatively, copolymerizations with *f*_0_^MAnh^ > 0.5 (Table 1, entries 7 and 8) were also conducted, but kinetic samples could not be analyzed using the same SEC protocol as entries 1–4 and as such are not included in Figure 1. The relevant monomer conversion vs time and semilogarithmic kinetic plots are included in the Supporting Information (Figure S2). Copolymerizations such as **U**SMAnh*M2* that employed *f*_0_^MAnh^ = 0.57 had a slightly higher apparent propagation rate (*k*_p_^app^ = 0.35 h^–1^) compared to copolymerizations with *f*_0_^MAnh^ = 0.50, resulting in quantitative monomer conversion within 6 h of polymerization. SMAnh copolymers synthesized using the universal RAFT agent and *f*_0_^STY^ > 0.50 were found to have generally lower *Đ* compared to those with *f*_0_^STY^ < 0.50, a phenomenon that has been reported for dithiobenzoate-mediated SMAnh RAFT copolymerizations.^34^ This can be observed for the exemplary copolymers **U**SMAnh*S*_*3k*_ and **U**SMAnh*M*_*3k*_ (Table 1, entries 9 and 10).

**Figure 2:**
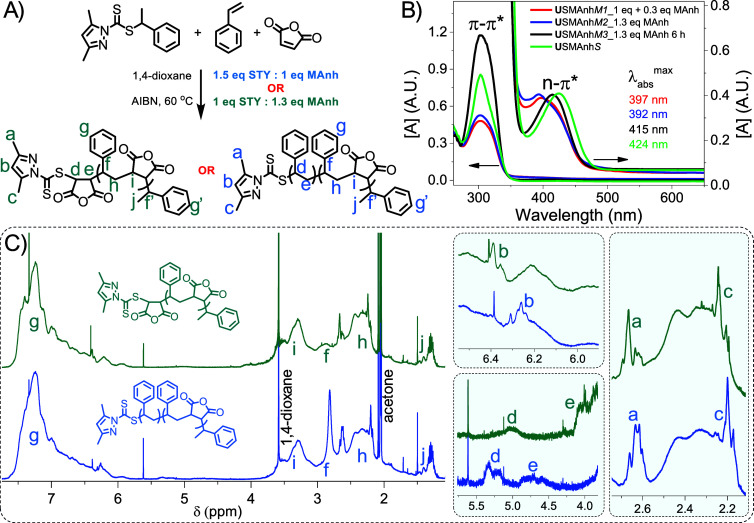
(A) Scheme for the universal RAFT-mediated copolymerization of STY and MAnh. (B) UV–vis spectroscopic analysis of **U**SMAnh*S* and **U**SMAnh*M* copolymers in 1,4-dioxane. (C) ^1^H NMR spectroscopic analysis (600 MHz, Bruker) of STY terminal **U**SMAnh*S* (blue) and MAnh terminal **U**SMAnh*M* (green) in (CD_3_)_2_CO, with insets indicating protons characteristic of the pyrazole Z-group adjacent to either STY/MAnh terminal monomer units.

To confirm the successful insertion of either MAnh or STY/*t*BuSTY at the ω-chain end (Figure 2A), the purified copolymers were dissolved in 1,4-dioxane or (CD_3_)_2_CO and characterized via UV–vis spectroscopy (Figure 2B) and ^1^H NMR spectroscopy (Figure 2C), respectively. All **U**SMAnh copolymers exhibited a π–π* absorbance band at 303 nm irrespective of whether the thiocarbonylthio group was adjacent to a MAnh or STY unit, but exhibited a blue shift in the spin forbidden n-π* absorbance band from 424 nm (STY-adjacent chromophore) to 415 nm (MAnh-adjacent chromophore). For copolymers **U**SMAnh*M1* and **U**SMAnh*M2*, quantitative monomer conversion was achieved within 10 and 6 h, respectively, but they were heated at 60 °C in the presence of AIBN and excess MAnh up to the time of quenching at 21 h. Alternatively, the **U**SMAnh*M3* copolymerization was quenched immediately after full monomer conversion was achieved (6 h).

The **U**SMAnh*M1*/*M2* copolymers were isolated with an off-white/pale orange-yellow discoloration, while the **U**SMAnh*M3* copolymer had the characteristic bright yellow coloration of SMAnh with a thiocarbonylthio group at the ω-chain end. UV–vis spectroscopic analysis of **U**SMAnh*M1* and **U**SMAnh*M2* showed that the n-π* absorbance band was shifted from 415 nm (for **U**SMAnh*M3*) to 392–397 nm (Figure 2B), suggesting that some change in the thiocarbonylthio group could have occurred with prolonged exposure to the polymerization reaction medium after quantitative monomer conversion was achieved. It should be noted that the SEC analysis of **U**SMAnh*M1*–*M3* polymers (Table 1) showed a discrepancy between *M*_n_^theo^ and *M*_n_^SEC^ (with high *Đ*) due to the utilization of a SEC protocol with suboptimal parameters for the analysis of SMAnh-type copolymers (discussed in the SI, Figure S3). For **U**SMAnh copolymers with MAnh at the ω-chain end, the protons characteristic of the 3,5-dimethylpyrazole Z-group (labeled “a”, “b”, and “c” in Figure 2) were shifted downfield relative to the same Z-group protons of **U**SMAnh with STY at the ω-chain end. Furthermore, the protons of the terminal monomer adjacent to the thiocarbonylthio moiety have characteristic chemical shifts (labeled “d” and “e” in Figure 2), a phenomenon that has been demonstrated by Moriceau *et al*. previously.^35^ The same analysis can be applied to the **(S-H**^**+**^**)**SMAnh and *t*BuSMAnh copolymers (Figures S4 and S5, respectively), where a MAnh at the ω-chain end also causes a downfield shift of the Z-group protons and a shift of the absorbance bands of the thiocarbonylthio moiety to slightly lower wavelengths (Figure S6).

### Deprotonation and Hydrolysis of Macro-RAFT Agents

**Figure 3:**
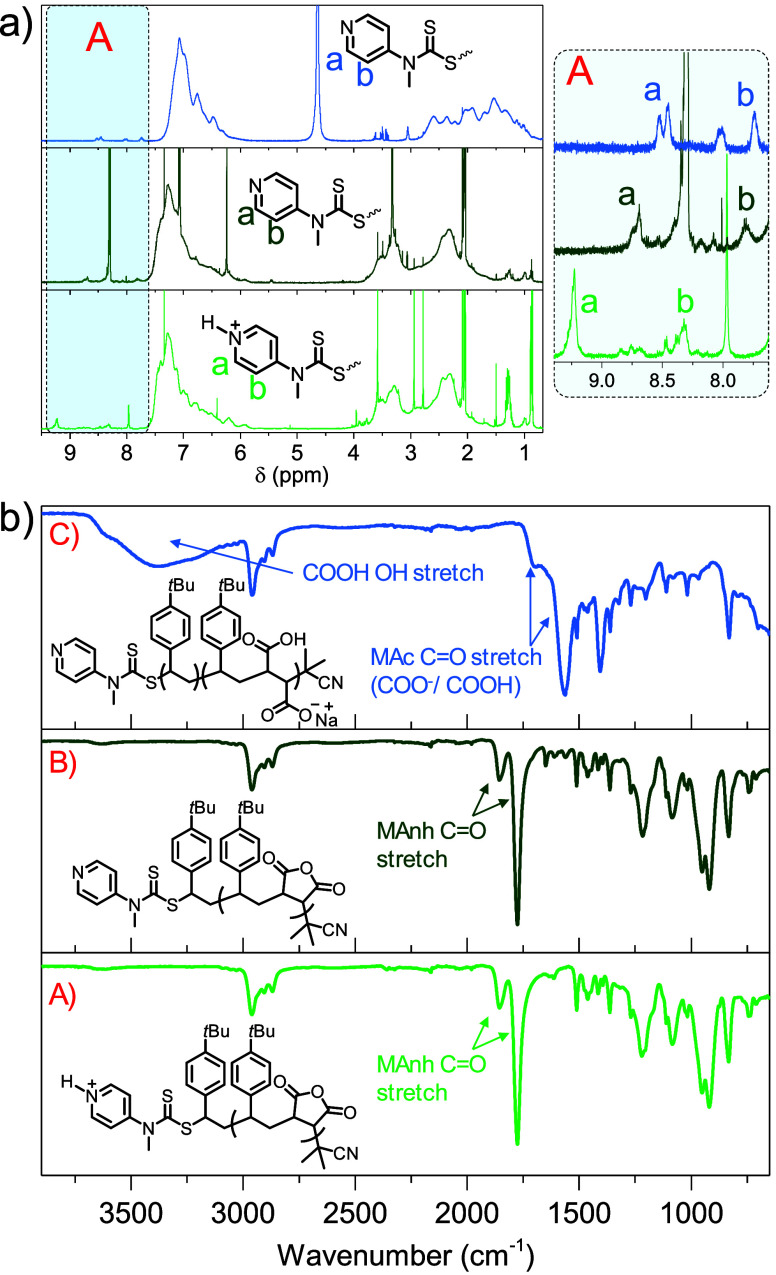
(a) ^1^H NMR spectra (600 MHz, Bruker) for **(S-H**^**+**^**)**SMAnh in (CD_3_)_2_CO (light green), **S**SMAnh in (CD_3_)_2_CO (dark green) post DMAP-mediated deprotonation, and **S**SMA in D_2_O (light blue) post Na_2_CO_3_-mediated deprotonation and hydrolysis, with the inset (A) indicating the pyridinyl protons between 7.7–9.4 ppm. (b) ATR-FTIR spectra for (A) **(S-H**^**+**^**)***t*BuSMAnh, (B) **S***t*BuSMAnh post DMAP-mediated deprotonation, and (C) **S***t*BuSMA following Na_2_CO_3_-mediated deprotonation and hydrolysis.

The hydrophobic **U**SMAnh or **U***t*BuSMAnh universal macro-RAFT agents can be used to mediate the polymerization of MAMs or LAMs in organic media. Alternatively, the MAnh repeat units along the SMAnh/*t*BuSMAnh backbone are amenable to alkaline hydrolysis, yielding their corresponding MAc form and producing an amphiphilic water-soluble macro-RAFT agent (**U**SMA/**U***t*BuSMA). For the switchable **(S-H**^**+**^**)**SMAnh or **(S-H**^**+**^**)***t*BuSMAnh macro-RAFT agents, the Z-group must be deprotonated to mediate the polymerization of LAMs, which can be achieved *in situ* using an organic base such as DMAP, yielding the hydrophobic **S**SMAnh/**S***t*BuSMAnh macro-RAFT agent. Alternatively, the Z-group can be deprotonated using aqueous Na_2_CO_3_, yielding the amphiphilic and water-soluble **S**SMA/**S***t*BuSMA macro-RAFT agent. This was demonstrated using **(S-H**^**+**^**)***t*BuSMAnh and **(S-H**^**+**^**)**SMAnh as exemplary macro-RAFT agents (Figure 3). Upon addition of DMAP, the protonated macro-RAFT agent (in acetone at 25 °C) underwent an immediate color change from bright yellow (characteristic of the protonated Z-group at the ω-chain end) to pale yellow/orange. ^1^H NMR spectroscopic analysis of the isolated **S***t*BuSMAnh showed that the pyridinyl protons characteristic of the protonated Z-group (H_a_ and H_b_, Figure 3a) had shifted upfield upon deprotonation with DMAP. Alternatively, the macro-RAFT agent (in acetone) was added to an aqueous Na_2_CO_3_ solution, causing precipitation of the copolymer and an immediate color change to pale yellow/orange. With continued stirring at 25 °C, the solubility of the copolymer in water increased as the MAnh repeat units underwent hydrolysis to MAc. The same characteristic upfield shift of the pyridinyl protons was observed, indicating that successful deprotonation of the Z-group had occurred (Figure 3a). **(S-H**^**+**^**)***t*BuSMAnh, the corresponding **S***t*BuSMAnh copolymer after DMAP-mediated deprotonation, and **S***t*BuSMA, the corresponding copolymer after Na_2_CO_3_-mediated deprotonation and hydrolysis, were analyzed via ATR-FTIR spectroscopy (Figure 3b). The MAnh repeat units along the SMAnh/*t*BuSMAnh backbone are unreactive toward tertiary amines, and as such, the C=O stretches at 1854 and 1776 cm^–1^ were retained post exposure to DMAP. After treatment with aqueous Na_2_CO_3_, however, these C=O stretches disappeared and were replaced with C=O stretches at 1697 and 1564 cm^–1^, and additionally, an OH stretch at 3677–3100 cm^–1^ was observed, indicative of the formation of carboxylic acid/carboxylate groups along the copolymer backbone. Similar hydrolysis procedures can be applied to the universal macro-RAFT agents, where similar ^1^H NMR and ATR-FTIR spectra are obtained (Figure S7).

### Synthesis of USMAnh-*b*-PVP

NVP is an example of a LAM with a nonconjugated vinyl bond, resulting in a highly reactive radical species as the NVP-based radical does not undergo resonance stabilization. RAFT agents that are less active toward radical addition such as xanthates (Z = OR’R”) and low-activity dithiocarbamates (Z = :NR’R”) are generally required to effectively control the RAFT-mediated polymerization of NVP, but some successful polymerizations of NVP using the universal or switchable dithiocarbamates have been reported.^23,25,36,37^ Performing a well-controlled RAFT polymerization of NVP presents several challenges, for example, the loss of NVP to dimerization side reactions or formation of dead chains via thermolysis or hydrolysis reactions occurring at the ω-chain end (summarized in Figure S8).^27,28^ Hydration of the vinyl bond of NVP facilitates the formation of the hydration dimer (Figure S8, structure 2). The formation of the unsaturated NVP dimer, which is catalyzed by protic hydrogen impurities, also depletes the NVP monomer, altering reaction stoichiometry (Figure S8, structure 4). Consequently, great care must be taken when polymerizing NVP in organic solvents by using dry organic solvents, avoiding protic species and keeping out moisture.

**U**SMAnhS (Table 2, entry 1) was synthesized by using freshly sublimed MAnh and distilled 1,4-dioxane followed by purification and immediate use as a macro-RAFT agent within 24 h of synthesis. The chain extension of **U**SMAnhS with PVP was attempted (Table 2, entry 2), with a monomer consumption of 96% determined via ^1^H NMR spectroscopy. However, the calculated monomer consumption primarily corresponded to the formation of hydration and unsaturated NVP dimers (29 and 65% abundance, respectively, with a remaining 6% unreacted NVP) as indicated in Figure S9. The significant abundance of the unsaturated dimer is likely due to the presence of MAc repeat units along the **U**SMAnhS backbone, which arise during the precipitation of the copolymer. To avoid exposure of the copolymer to water, subsequent polymerizations conducted chain extension of the macro-RAFT agent with PVP *in situ* via addition of AIBN and freshly distilled NVP followed by deoxygenation using a freeze–pump–thaw protocol (Table 2, entries 3–6).

Following the synthesis of **U**SMAnhS* (Table 2, entry 3), an excess of MAnh and STY (1 and 3 equiv relative to RAFT agent, respectively) remained in the polymerization mixture for the *in situ* chain extension with PVP. During chain extension, the excess MAnh was fully consumed, an additional 2 STY units were inserted, and 4% NVP conversion (DP = 2) was obtained (of which 0.2% can be attributed to the formation of the NVP hydration dimer, Figure S10, H_8_). Either the excess STY monomer potentially retards the RAFT-mediated polymerization of NVP, or the polymerization is inhibited due to the poor reinitiating efficiency of styrene-based radicals of the macro-R-group.^38^ A sample was withdrawn from the polymerization reaction at 24 h and analyzed by ^1^H NMR spectroscopy (Figure S10). Protons characteristic of the 3,5-dimethylpyrazole Z-group as well as the terminal MAnh monomer unit adjacent to the thiocarbonylthio group (H_b_, H_d_, and H_d_ respectively, Figure S10), which were present prior to the chain extension polymerization, were not observed in the 24 h sample. This could be due to the increased lability of the thiocarbonylthio group following insertion of ∼2 NVP repeat units, explored in more detail below. Notably, protons corresponding to the unsaturated NVP dimer were absent during *in situ* chain extension.

**Figure 4:**
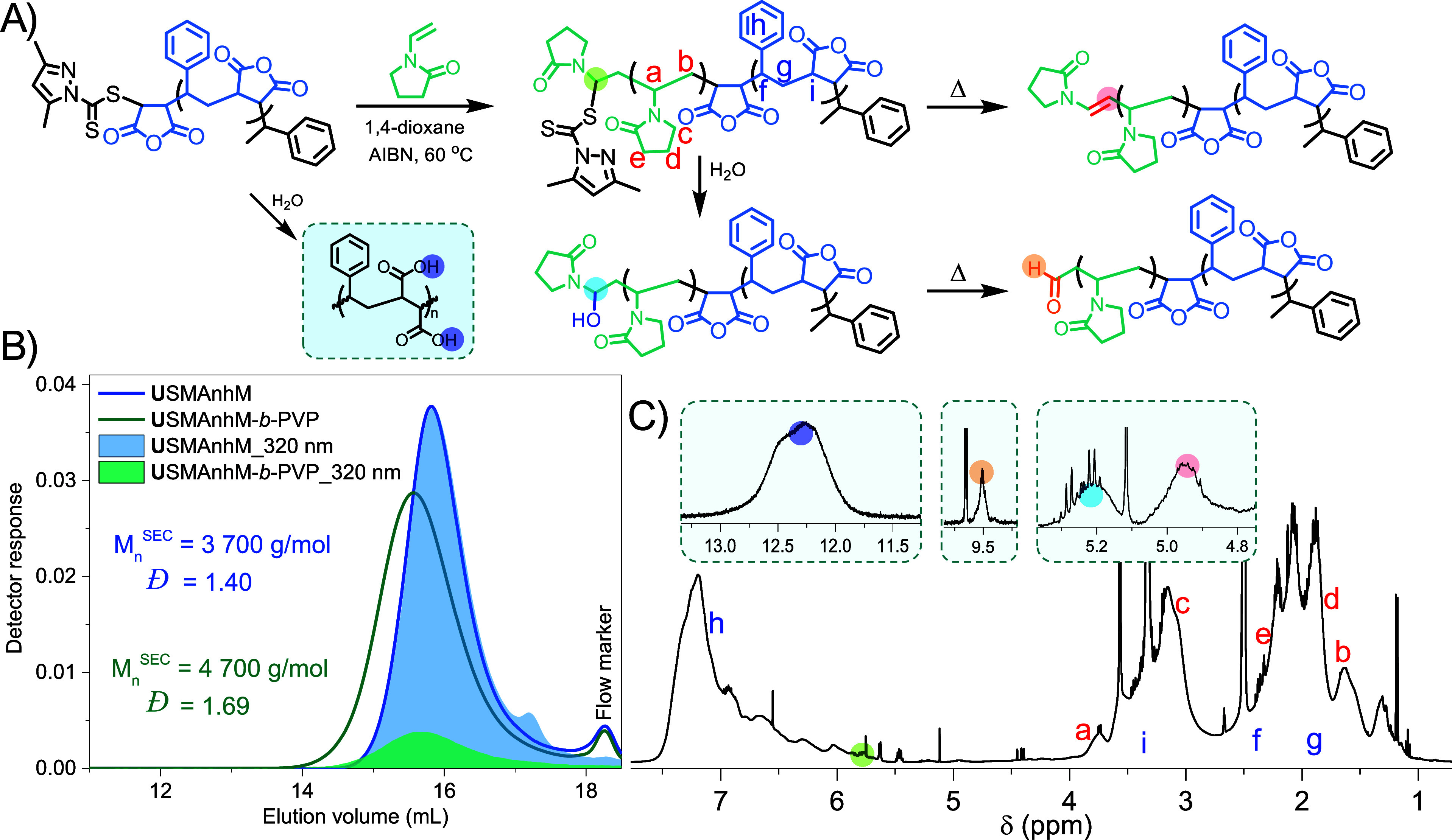
(A) Scheme for the chain extension of **U**SMAnhM* with PVP and subsequent thiocarbonylthio group cleavage via thermolysis and hydrolysis. (B) SEC analysis for **U**SMAnhM* and its corresponding **U**SMAnh*M*-*b*-PVP block copolymer (synthesized using a one-pot two-step procedure), with DMF (2 mM LiBr) as the mobile phase and SMAnh calibration standards. (C) ^1^H NMR spectroscopic analysis (600 MHz, Bruker) of **U**SMAnh*M*-*b*-PVP in (CD_3_)_2_SO with insets indicating protons associated with MAc backbone functional groups and aldehyde, hydroxy, and unsaturated functional ω-chain ends.

To ensure the absence of excess STY in the polymerization mixture after the synthesis of the macro-RAFT agent and a macro-R-group with a MAnh-based radical, an equimolar STY-MAnh comonomer feed was prepared, which yielded α^STY^ = 100% and α^MAnh^ = 91% (**U**SMAnhM*, Table 2, entry 5) due to the partial volatilization of STY during deoxygenation. **U**SMAnh*M* was chain extended with PVP *in situ* (α^NVP^ = 35%, DP = 21), and the isolated **U**SMAnh*M*-*b*-PVP copolymer was characterized via ^1^H NMR, DOSY, NMR spectroscopy, and SEC. The ^1^H NMR spectrum showed protons characteristic of PVP (H_a-e_, Figure 4C) and SMAnh (H_f-i_), but protons characteristic of the Z-group could not be observed directly. Instead, protons corresponding to unsaturated (4.95 ppm), hydroxy (5.20 ppm), or aldehyde (9.50 ppm) functional groups at the ω-chain were observed. The thermolysis or hydrolysis of xanthate Z-groups from the ω-chain end of PVP is well-known and was first demonstrated in detail by Pound *et al*., where conversion of hydroxy chain ends to aldehyde functional chain ends could be achieved with additional heating of the polymer.^28^ Water could have been introduced into the polymerization vessel via the significantly hygroscopic NVP monomer, which would account for the conversion of some MAnh units along the **U**SMAnh*M* backbone into MAc units (12.25 ppm, Figure 4C) as well as hydrolysis of the thiocarbonylthio group. This polymerization was conducted at 60 °C, which could facilitate thermolysis of the thiocarbonylthio group upon the insertion of NVP at the ω-chain end.

SEC analysis of **U**SMAnh*M*-*b*-PVP and the **U**SMAnh*M* macro-RAFT agent showed a clear shift of the block copolymer’s molecular weight distribution to lower elution volumes, and DOSY NMR analysis showed that protons characteristic of SMAnh and PVP had similar diffusion coefficients (Figure S11), indicating that chain extension had been successful. An estimate of thiocarbonylthio group loss was determined by using the reduction in peak area obtained from the UV detector response (Figure S12). Theoretically, chain extension should decrease the peak area of the **U**SMAnh*M*-*b*-PVP UV distribution by 31% as the weight contribution of the chromophore decreases (with the assumption that the molar extinction coefficient undergoes a minimal change). Experimentally, a reduction in UV distribution peak area of 86% following chain extension was observed, which (in combination with the ^1^H NMR spectroscopic analysis) would suggest that significant loss of the thiocarbonylthio group has indeed occurred.

### Synthesis of (U/S)SMA-*b*-PVP

To limit the thermolysis of the thiocarbonylthio group upon insertion of NVP at the ω-chain end, the RAFT-mediated polymerization of NVP was conducted at ambient temperature and additionally in a buffered aqueous medium to limit acid-catalyzed dimerization of NVP. This was investigated using the hydrolyzed water-soluble derivatives of the universal/switchable macro-RAFT agents (i.e., **U**SMA and **S**SMA) in PBS (pH = 7.4) at 30 °C for 24 h using a redox initiating pair constituting *t*BuOOH and Na_2_SO_3_ (3). In the neutral aqueous conditions used during chain extension (*p*H 7.4), the SMA and *t*BuSMA macro-CTAs were shown to be partially ionized (MAc unit *p*K_a1_ = 4.5, *p*K_a2_ = 8.9 and *p*K_a1_ = 5.6, *p*K_a2_ = 8.0, respectively, Figure S13).

**Table 3:** Monomer Conversions and Molecular Weight Analysis of SMA Macro-RAFT Agents and Corresponding Block Copolymers Synthesized via Aqueous **U**/**S**-Mediated Polymerization

entry	sample^a^	reagent ratio^b^	α^STY^, α^MAnh^ (%)^c^	α^NVP^ (%)^c^	*M*_n_^theo^ (g/mol)^d^	*M*_n_^SEC^ (g/mol)^e^	*Đ*^e^
**1**	**U**SMA*M*_25_	1:25:32:0.2	100, 81		5400	6400	1.34
**2**	**U**SMA*M*_25_-*b*-PVP_311_	1:494:0.5:0.5		**63**	40,500	33,100	2.77
**3**	**U**SMA*S*_25_	1:16:12:0.2	95, 100		3000	1600	1.29
**4**	**U**SMA*S*_25_-*b*-PVP_361_	1:495:1:1		**73**	43,400	23,800	2.56
**5**	**S**SMA*M*_25_	1:25:32:0.2	100, 78		6000	10,000	1.43
**6**	**S**SMA*M*_25_-*b*-PVP_324_	1:463:1:1		**70**	41,800	37,400	2.63
**7**	**S**SMA*S*_25_	1:32:25:0.2	84, 100		5500	9100	1.35
**8**	**S**SMA*S*_25_-*b*-PVP_335_	1:479:1:1		**70**	43,000	35,900	1.92

a **U** and **S** indicate the universal and switchable methyl 2-((methyl (pyridin-4-yl) carbamothioyl) thio) propanoate RAFT agents, respectively. SMA(*S*/*M*) specifies the terminal monomer unit, and subscript values indicate the DP of each block.

b [RAFT]/[STY]/[MAnh]/[AIBN] for macro-RAFT syntheses and [macro-RAFT]/[NVP]/[Na_2_SO_3_]/[*t*BuOOH] for block copolymer synthesis.

c Determined via ^1^H NMR spectroscopy using 1,3,5-trioxane as the internal standard and eq S1 (for SMA samples) and eq S3 (for SMA-*b*-PVP samples).

d Calculated using eq S2 (for SMA samples) and eq S4 (for SMA-*b*-PVP samples).

e All samples methylated and analyzed via SEC using DMF (0.05 M LiBr, 40 °C) as eluent and PMMA calibration standards.

**Figure 5:**
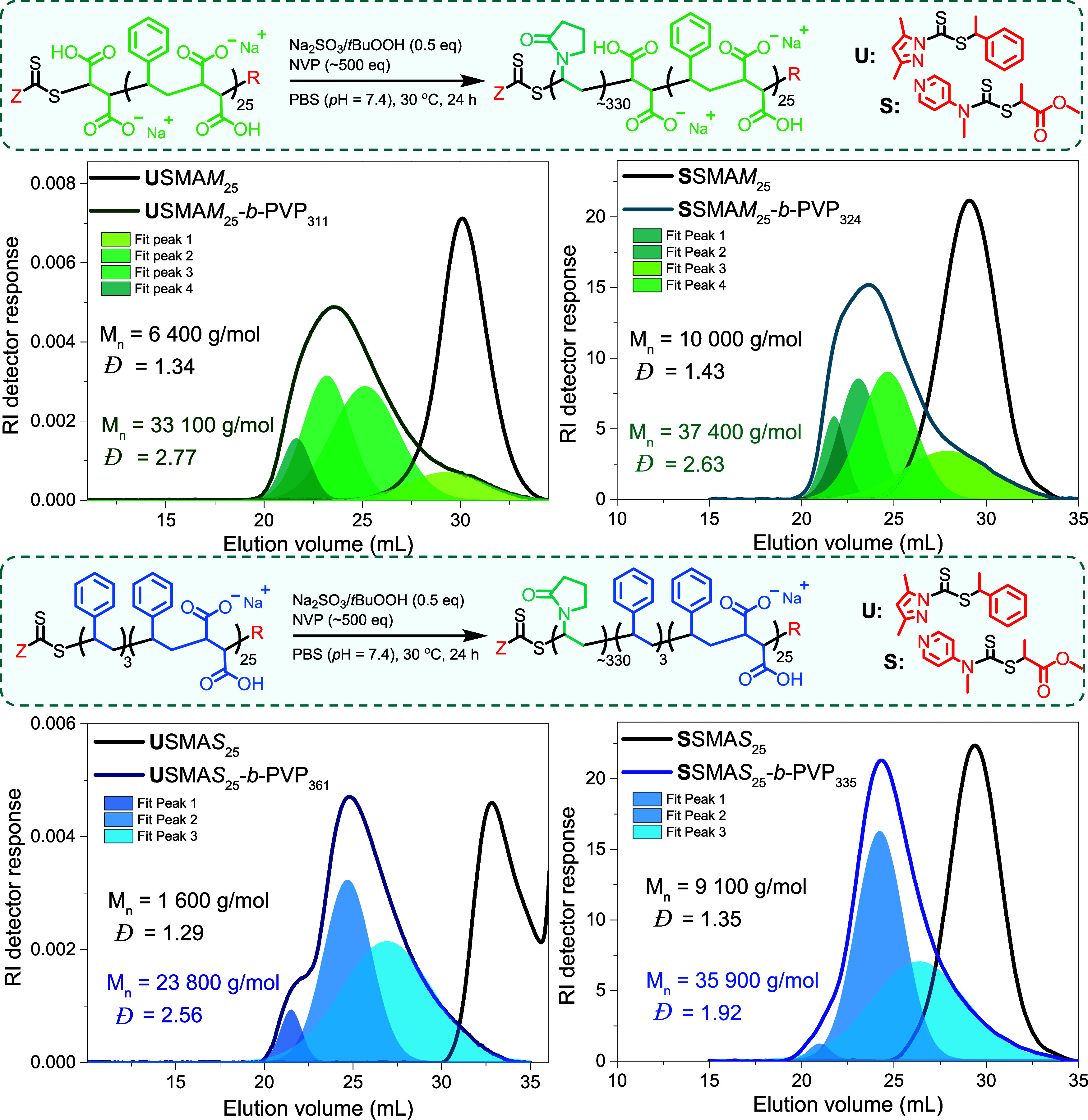
SEC analyses of methylated SMA and SMA-*b*-PVP copolymers using DMF (0.05 M LiBr, 40 °C) as the mobile phase and PMMA calibration standards. **U**SMA macro-RAFT-mediated block copolymerizations are presented on the left and **S**SMA on the right, with the macro-R-groups possessing either a terminal MAc unit (top) or a terminal STY unit (bottom).

All SMA-*b*-PVP block copolymers were characterized via ^1^H NMR, DOSY NMR, ATR-FTIR, and UV–vis spectroscopy as well as SEC, where SMA and corresponding SMA-*b*-PVP copolymers underwent methylation prior to SEC analysis (Figure S14, Figure 5). For all block copolymers (entries 2, 4, 6, and 8, Table 3), SEC analysis showed a shift in the molecular weight distribution to lower elution volumes relative to the SMA macro-RAFT agent (Figure 4), and DOSY NMR analysis indicated that that protons characteristic of SMA and PVP had similar diffusion coefficients (Figure 7), suggesting that successful chain extension had occurred. All samples exhibited a high-molecular-weight shoulder, as well as low-molecular-weight tailing, resulting in significant broadening of the molecular weight distribution (*Đ* = 1.9–2.8). This broadening could be the result of several contributing factors, such as the prevalence of termination events at higher NVP conversions, the loss of thiocarbonylthio groups prior to chain extension (during the hydrolysis of SMAnh macro-RAFT agents), the loss of thiocarbonylthio groups during polymerization (hydrolysis and thermolysis reactions at the ω-chain end), or poor reinitiating efficiency of the SMA macro-R-groups.

**Figure 6:**
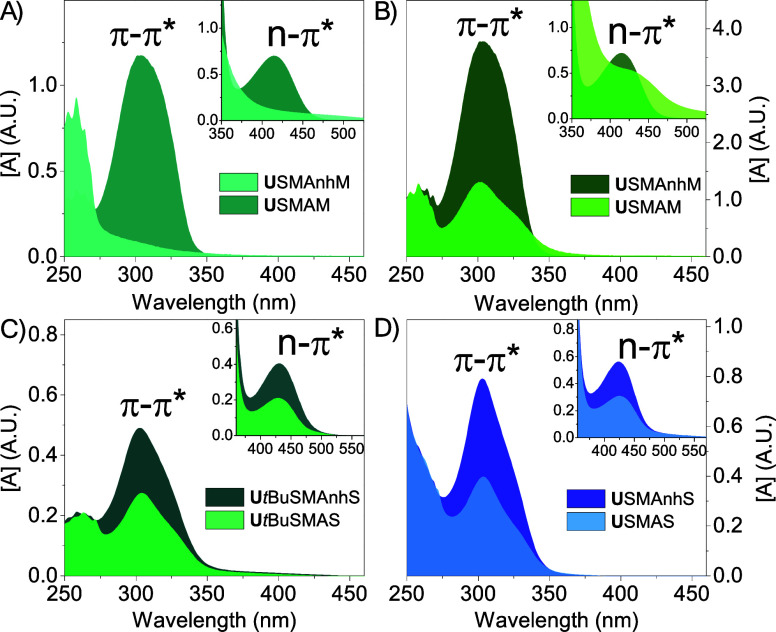
UV–vis spectroscopic analysis of (A) **U**SMAnh*M* in 1,4-dioxane and **U**SMA*M* in DI water (DMSO/H_2_O-mediated hydrolysis at 80 °C for 24 h, yielding a 100% reduction in π–π* absorbance band); (B) **U**SMAnh*M* in 1,4-dioxane and **U**SMA*M* in DI water (Na_2_CO_3_(aq)-mediated hydrolysis at 25 °C for 24 h, yielding a 66% reduction in π–π* absorbance band); (C) **U***t*BuSMAnh*S* in 1,4-dioxane and **U***t*BuSMA*S* in DI water (Na_2_CO_3_(aq)-mediated hydrolysis at 25 °C for 24 h, yielding a 45% reduction in π–π* absorbance band); and (D) **U**SMAnh*S* in 1,4-dioxane and **U**SMAS in DI water (NaOH(aq)-mediated hydrolysis at 25 °C for 24 h, yielding a 49% reduction in π–π* absorbance band). Samples were analyzed at 20 and 0.2 mg/mL in 1,4-dioxane/DI water for resolution of the n-π* and π–π* absorbance band, respectively.

The hydrolytic lability of thiocarbonylthio groups at the ω-chain end is affected by the nature of the RAFT agent used, the monomer type adjacent to the thiocarbonylthio group, the molecular weight of the polymer, the solvent composition of the medium, as well as the pH and temperature of the medium (where the length of exposure at these given conditions also plays a role).^28,39−45^ The SMAnh macro-RAFT agents undergo alkaline hydrolysis at ambient temperatures followed by dialysis in DI water for up to 72 h before lyophilization. As these conditions have been shown previously to exacerbate the hydrolysis of dithioester, trithiocarbonate, and xanthate Z-groups, it is reasonable to expect that the universal and switchable dithiocarbamate Z-groups would be susceptible to hydrolysis as well. The extent of potential thiocarbonylthio group hydrolysis was assessed via UV–vis spectroscopy (Figure 6, Figure S15).

**Figure 7:**
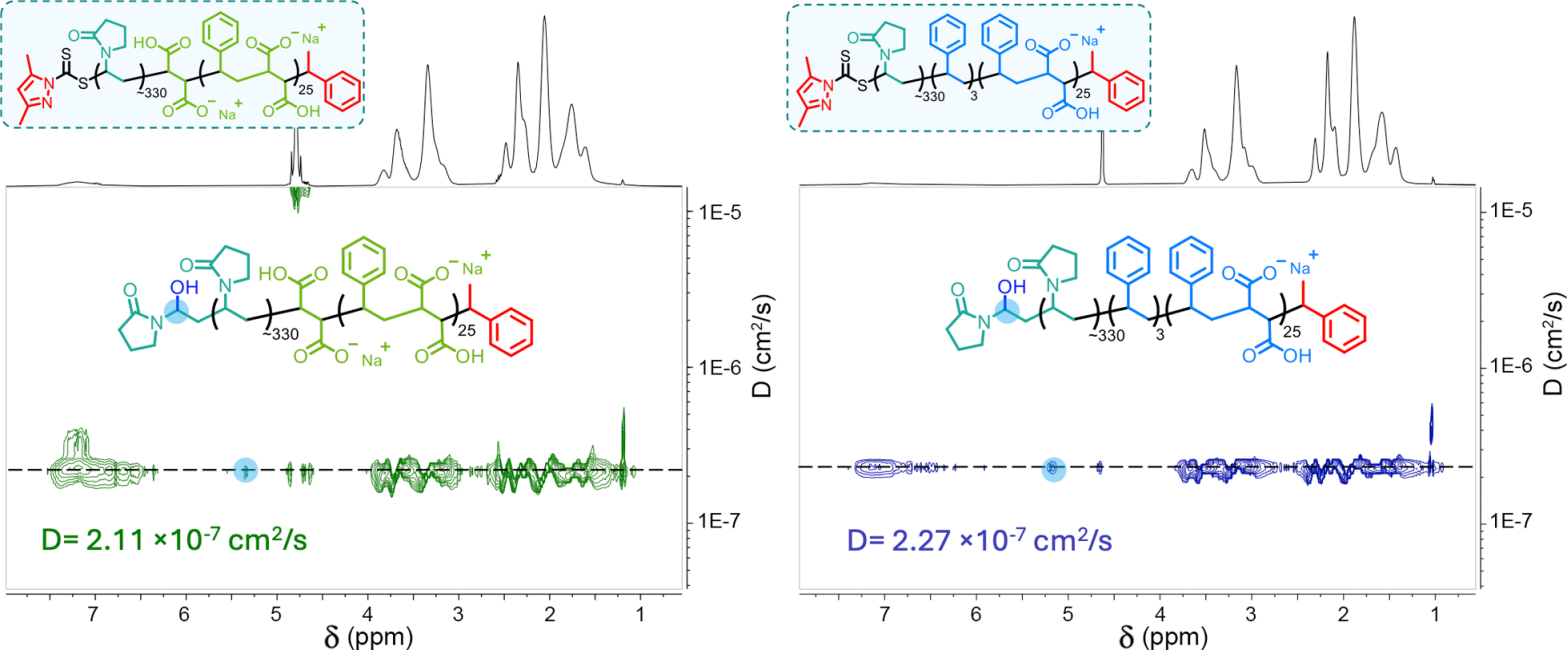
DOSY NMR spectroscopic analysis (400 MHz, Varian) of **U**SMA*M*_25_-*b*-PVP_311_ (left) and **U**SMA*S*_25_-*b*-PVP_361_ (right) in D_2_O.

The universal dithiocarbamate Z-group appeared to have significant hydrolytic lability under various hydrolysis conditions. Simply heating **U**SMAnh*M* in a H_2_O/DMSO mixture at 80 °C allowed for the complete disappearance of the thiocarbonylthio group π–π* absorbance band, while Na_2_CO_3_(aq) mediated hydrolysis at ambient temperature resulted in a 66% reduction in the π–π* absorbance band. Similar alkaline hydrolysis conditions applied to **U**SMAnh*S*/**U***t*BuSMAn*S* resulted in π–π* absorbance band reductions between 45 and 49%, indicating that the thiocarbonylthio group might have higher hydrolytic stability when adjacent to a STY monomer unit. It must be noted that these percentages might not accurately quantify the removal of the thiocarbonylthio group. **U**SMAnh/**U***t*BuSMAnh macro-RAFT agents are analyzed in 1,4-dioxane, while the corresponding **U**SMA/**U***t*BuSMA copolymers were analyzed in water (where the transformation of the terminal MAnh unit to its MAc form might also adjust the molar extinction coefficient of the thiocarbonylthio group chromophore). Nevertheless, the SMA-*b*-PVP copolymers synthesized using SMA macro-RAFT agents with a MAc unit at the ω-chain end appear to have more significant low-molecular-weight tailing at elution volumes similar to the SMA macro-RAFT agent, suggesting a larger proportion of dead chains compared with STY terminal SMA macro-RAFT agents. Furthermore, the **U**SMA*M*_25_-*b*-PVP_311_ DOSY NMR spectrum would suggest that a proportion of dead SMA chains originated during alkaline hydrolysis (to a greater extent than **U**SMA*S*_25_-*b*-PVP_361_), as a proportion of the aromatic protons associated with the SMA block (7.25 ppm) lack the corresponding PVP protons at the same diffusion coefficient (Figure 7).

The experimental findings are further corroborated with computational DFT calculations using **S** as the representative RAFT agent (Table S4, Figure S21). The Δ*G* for thiocarbonylthio group hydrolysis is more favorable for polymer chains terminated with MAc (Δ*G* = −33.99 kcal·mol^–1^) in comparison to that of STY (Δ*G* = −31.95 kcal·mol^–1^). To the best of our knowledge, there is no indication in the literature as to the mechanism of the end group hydrolysis to afford a hydroxy functionality at the polymer chain end. Taking note of both the experimental and computational findings, it seems plausible that the hydrolysis of the thiocarbonylthio group takes place via an S_N_2-type mechanism. It is noted that the reaction is significantly favored in basic media (Δ*G*_basic_ = −31.95 kcal·mol^–1^ vs Δ*G*_acidic_ = 0.16 kcal·mol^–1^ for the STY terminated polymers), i.e., a stronger nucleophile present, and that the hydrolysis occurs more readily on a less stabilized carbon atom (MAc). This strongly suggests an S_N_2 mechanism compared to an S_N_1 reaction (preferential in acidic media and occurring on a more stabilized carbon position).

The occurrence of thiocarbonylthio group hydrolysis is likely exacerbated upon insertion of NVP, which creates hydroxy-functionalized chains that do not partake further in the RAFT process. Computational calculations indeed suggest that incorporation of NVP into the polymer backbone significantly increases the lability of the thiocarbonylthio group (Δ*G*_NVP hydrolysis_ = −36.43 kcal·mol^–1^ vs Δ*G*_MAc hydrolysis_ = −33.99 kcal·mol^–1^ for **S**). The methine proton characteristic of the NVP unit at hydroxy-functional ω-chain ends could be observed during DOSY NMR analysis (with similar diffusion coefficients to associated BCPs), suggesting a slower rate of thiocarbonylthio group hydrolysis compared to the rate of polymerization; *vide infra* (Figure 7). A difference in the reinitiating efficiency of the macro-RAFT agents is also possible depending on whether the terminal monomer unit for the macro-R-group is STY or MAc, but this is difficult to discern due to the difference in the hydrolytic stability of the thiocarbonylthio groups at respective ω-chain ends.

### Dithiocarbamate Z-Group Hydrolysis

**Figure 8:**
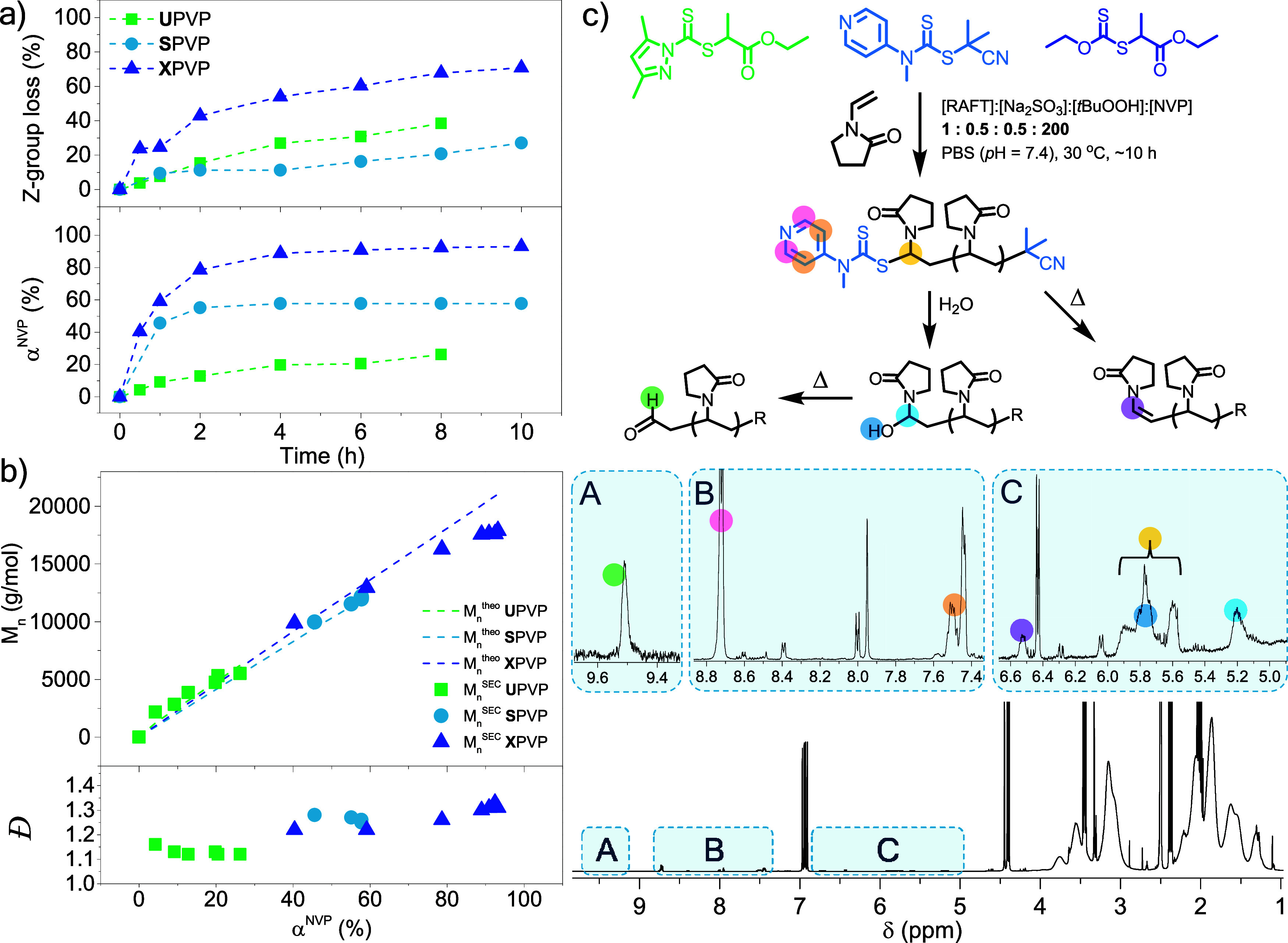
(a) NVP conversion and thiocarbonylthio group loss vs time determined via ^1^H NMR spectroscopic analysis of crude kinetic samples and lyophilized kinetic samples, respectively (Figure S16–S18). (b) SEC analysis of lyophilized kinetic samples using DMF (2 mM LiBr, 60 °C) as the mobile phase and PMMA calibration standards. c) Exemplary ^1^H NMR spectroscopic analysis of a lyophilized **S**PVP kinetic sample (10 h) in (CD_3_)_2_SO with associated structures and spectral insets indicating the variety of ω-chain ends formed during the aqueous RAFT-mediated polymerization of NVP.

Several notable studies have reported the transformation of xanthate moieties at PVP ω-chain ends, but no detailed investigation of the transformation of universal- and switchable-type dithiocarbamates has been reported. To investigate whether the relevant dithiocarbamate Z-groups undergo transformation similar to that reported for xanthates and determine whether this deleteriously impacts the RAFT-mediated polymerization of NVP, kinetic experiments were performed using three different RAFT agents (Figure 8, Table 4). All polymerizations were conducted in PBS (pH = 7.4) (30 w/v%) at 30 °C, where kinetic samples were withdrawn at specified time intervals for entries 1–3 (Table 4). Kinetic sampling generally entailed the withdrawal of ∼0.8 mL of the polymerization mixture with a degassed syringe followed by the dilution of 0.2 mL in (CD_3_)_2_SO and lyophilization of the remaining 0.6 mL for subsequent SEC and ^1^H NMR spectroscopic analyses.

**Table 4:** Monomer Conversions, Thiocarbonylthio Group Removal Determination, and Molecular Weight Analysis for Aqueous RAFT-Mediated Homopolymerization of NVP

entry	sample^a^	time (h)	reagent ratio^b^	α^NVP^ (%)^c^	%EG loss^d^	*M*_n_^theo^ (g/mol)^e^	*M*_n_^SEC^ (g/mol)^f^	*Đ*^f^
**1**	**X**PVP	10	1:201:0.5:0.5	93	71 (10 h)	21,000	17,900	1.31
**2**	**U**PVP	24	1:204:0.5:0.5	26	85 (24 h)	6200	5500	1.12
**3**	**S**PVP	26	1:181:0.5:0.5	58	56 (62 h)	11,900	12,200	1.25
**4**	**U**PVP2	12	1:50:0.2:0.2	33		2100		
**5**	**S**PVP2	18	1:45:0.5:0.5	90	43 (66 h)	4800	2900	1.22
**6**	**S**PVP3	24	1:45:0.5:0.5	88	57 (72 h)	4700	3600	1.37

a **X**, **U**, and **S** indicate that a xanthate, universal, or switchable RAFT agent was used (specified in Figure 8).

b [RAFT]/[NVP]/[Na_2_SO_3_]/[*t*BuOOH].

c Determined via ^1^H NMR spectroscopy using DMF as internal standard and eq S3.

d Calculated using ^1^H NMR analysis of kinetic samples (outlined in Figures S16–S18 and Table S1–S3).

e Calculated using eq S4.

f Determined via SEC analysis using DMF (2 mM LiBr, 60 °C) (entries 1–3), DMF (0.05 M LiBr, 40 °C) (entries 5 and 6), and PMMA calibration standards.

The xanthate-mediated polymerization of NVP (**X**PVP, Table 4) yielded near quantitative monomer conversion within 10 h, with significant hydrolysis of the thiocarbonylthio group occurring throughout the polymerization (Figure 8a). The loss of xanthate moieties was assessed via ^1^H NMR analysis of the lyophilized kinetic samples in (CD_3_)_2_SO, where the Z-group methylene protons (CH_3_C***H*_2_**OC=S–, ∼4.6 ppm, Figure S16) and the methine proton of the terminal NVP unit gradually decreased in intensity, with a corresponding increase in the intensity of signals corresponding to hydroxy-functional ω-chain ends (***H***O(N)CHCH_2_–, ∼5.7 ppm; HO(N)C***H***CH_2_–, ∼5.2 ppm) and aldehyde-functional ω-chain ends (OC***H***CH_2_–, ∼9.5 ppm). After 10 h at 30 °C, the isolated polymer constituted 29% xanthate-functional, 69% hydroxy-functional, and 2% aldehyde-functional PVP (Table S1). This aligns with previous reports where quantitative ω-chain end hydrolysis was obtained by stirring xanthate-functional PVP in distilled water at 40 °C for 16 h.

SEC analysis of the lyophilized kinetic samples showed a linear evolution of *M*_n_^SEC^ with monomer conversion, which correlated well with *M*_n_^theo^ at α^NVP^ < 60% (Figure 8b). At higher NVP conversion, low-molecular-weight tailing could be observed in the molecular weight distribution (without a corresponding UV signal at 290 nm), which potentially corresponds to the formation of dead hydroxy-functional chains throughout the polymerization (Figure S19).

A similar kinetic experiment was conducted using a switchable RAFT agent (**S**PVP, Table 4), yielding 58% monomer conversion (lower than comparable polymerizations conducted without sampling, entries 5 and 6, Table 4). ^1^H NMR spectroscopic analysis of the kinetic samples in (CD_3_)_2_SO revealed similar signals characteristic of hydroxy-functional and aldehyde-functional ω-chain ends as well as unsaturated chain ends (Figure 8c, Figure S17) but with comparatively lower thiocarbonylthio group loss observed throughout the polymerization compared to **X**PVP. After 10 h of polymerization at 30 °C, the total thiocarbonylthio group loss was determined to be 27%; it had increased to 47% at 26 h and was ultimately estimated at 56% after the polymerization mixture was dialyzed in water at ambient temperature for an additional 36 h. **S**PVP samples characterized via SEC showed a linear evolution in *M*_n_^SEC^ as a function of monomer conversion, excellent correlation between *M*_n_^SEC^ and *M*_n_^theo^, and a general decrease in *Đ* with increasing α^NVP^ (Figure 8b). Despite the significant loss of thiocarbonylthio groups during the polymerization, within the 2 h period that the highest monomer conversion is obtained, only 11% of the dithiocarbamate chain ends are lost, yielding a relatively well-controlled RAFT-mediated polymerization (albeit with poor chain end fidelity in the final purified sample).

The same kinetic experiment was conducted using a universal dithiocarbamate (**U**PVP) but with a propionate R-group, as opposed to the 1-phenyl ethyl R-group primarily employed in this study, due to the latter’s inefficiency for the RAFT-mediated polymerization of NVP and its limited solubility in the aqueous polymerization medium. Notably, **U**PVP exhibited retarded polymerization kinetics compared to **X**PVP, which differs only in the thiocarbonylthio group employed, yielding only 26% monomer conversion within 8 h. A similar polymerization (**U**PVP2) conducted without kinetic sampling yielded similar α^NVP^ (33%) within 12 h, suggesting that retardation was not caused by inadvertent oxygen contamination. The **U**PVP dithiocarbamate ω-chain end underwent hydrolysis yielding hydroxy-functional chain ends, and consequently aldehyde-functional chain ends, throughout the polymerization (39% at 8 h) and to a greater extent (**U**PVP_24 h_ = 85%) than **S**PVP (47% at 26 h) (Figure S18). Nevertheless, a mostly linear evolution of *M*_n_^SEC^ with increasing monomer conversion is obtained, with a reasonable correlation observed between *M*_n_^SEC^ and *M*_n_^theo^ (Figure 8b).

The universal and switchable dithiocarbamate ω-chain ends appear to have slightly improved stability compared to xanthates in aqueous environments at ambient temperatures, providing adequate control over the RAFT-mediated polymerization of NVP but yielding polymers with poor end-group fidelity. Overall, a lower relative rate of hydrolysis compared to the rate of polymerization was demonstrated above (Figure 8a), accounting for the methine NVP proton at hydroxy-functional ω-chain ends having similar diffusion coefficients to SMA-*b*-PVP protons (Figure 7). The hydrolysis of dithiocarbamate Z-groups during the chain extension of **U**SMA/**S**SMA with PVP in water is likely not the principal contributing factor for the broad molecular weight distributions obtained for the SMA-*b*-PVP copolymers, as reasonable control over the RAFT-mediated polymerization of NVP was demonstrated. For the block copolymerizations outlined in Table 3, it appears that the primary reason for poorly controlled polymerization is the loss of thiocarbonylthio groups during alkaline hydrolysis of the macro-RAFT agents’ MAnh residues, which was intended to make the macro-RAFT agent water-soluble, yielding a large proportion of dead chains from the beginning of the block copolymerization. Control over the block copolymerization was then further impeded by the additional hydrolysis of thiocarbonylthio groups while chain extending with PVP, a possibility demonstrated above.

### BCPs with Smaller Block Ratios

The hydrolysis of dithiocarbamate Z-groups and the subsequent poorly controlled RAFT-mediated polymerization of NVP were investigated above, applying large block ratios to facilitate the separation of chain-extended polymer from dead chains. The exploration of smaller block ratios (Table 5) revealed interesting solution properties of the SMA/*t*BuSMA macro-RAFT agents, investigated using DLS, ^1^H NMR, and DOSY NMR spectroscopy. SMA/*t*BuSMA macro-RAFT agents were synthesized with an excess of STY to facilitate an average insertion of ∼3 STY/*t*BuSTY units at the ω-chain end (estimated via ^1^H NMR analysis), thus improving the hydrolytic stability of the dithiocarbamate Z-group (Table 5, entries 1, 3 and 5). Corresponding block copolymers were synthesized using the same procedure described for experiments outlined in Table 3 and characterized via SEC, ^1^H NMR spectroscopy, and DOSY NMR spectroscopy.

**Table 5:** Monomer Conversion and Molecular Weight Analysis for SMA/*t*BuSMA Macro-RAFT Agents and Their Corresponding Low-Molecular-Weight Block Copolymers

entry	sample^a^	reagent ratio^b^	α^STY^, α^MAnh^ (%)^c^	α^NVP^ (%)^c^	*M*_n_^theo^ (g/mol)^d^	*M*_n_^SEC^ (g/mol)^e^	*Đ*^e^
1	**U***t*BuSMAS_25_	1:25(+7):25:0.2	86, 100		7100	7900	1.30
**2**	**U***t*BuSMAS_25_-*b*-PVP_34_	1:45:0.5:0.5		76	11,400	10,600	1.67
3	**S***t*BuSMAS_25_	1:25(+7):25:0.2	87, 100		7200	8100	1.27
**4**	**S***t*BuSMAS_25_-*b*-PVP_13_	1:15:0.5:0.5		90	9100	9000	1.56
5	**S**SMAS_25_	1:25(+7):25:0.2	87, 100		5600	6800	1.39
**6**	**S**SMAS_25_-*b*-PVP_12_	1:15:0.5:0.5		83	7400	7400	1.70

a **U** and **S** indicate the universal and switchable RAFT agents, respectively. SMA/*t*BuSMA(S/M) specifies the terminal monomer unit, and subscript values indicate the DP of each block.

b [RAFT]/[STY]/[MAnh]/[AIBN] for SMA samples or [macro-RAFT]/[NVP]/[Na_2_SO_3_]/[*t*BuOOH] for block copolymer samples.

c Determined via ^1^H NMR spectroscopy using 1,3,5-trioxane as the internal standard and eq S1 (for SMA samples) and eq S3 (for SMA-*b*-PVP samples).

d Calculated using eq S2 (for SMA samples) and eq S4 (for SMA-*b*-PVP samples).

e All samples methylated and analyzed via SEC using DMF (0.05 M LiBr, 40 °C) as eluent and PMMA calibration standards.

**Figure 9:**
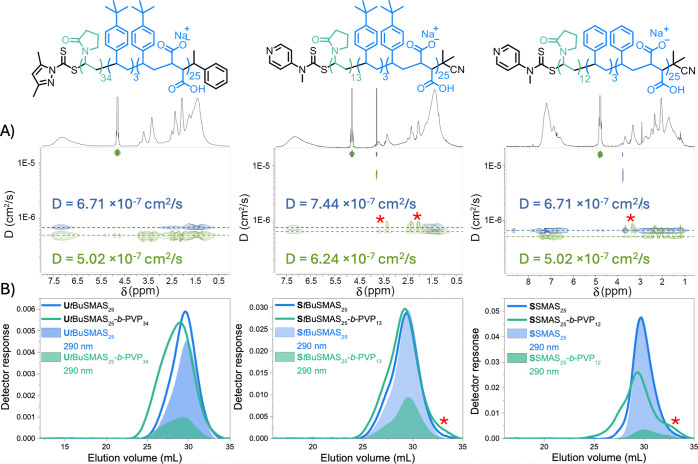
(A) DOSY NMR spectroscopic analysis (400 MHz, Varian) of SMA-*b*-PVP and *t*BuSMA-*b*-PVP copolymers in D_2_O, with blue data at higher diffusion coefficients representing the relevant SMA/*t*BuSMA macro-RAFT agent. (B) SEC analysis of methylated SMA-*b*-PVP and *t*BuSMA-*b*-PVP copolymers using DMF (0.05 M LiBr, 40 °C) as the mobile phase.

All block copolymers (Table 5, entries 2, 4, and 6) exhibited molecular weight distributions shifted to lower elution volumes compared to the corresponding macro-RAFT agent, and characteristic protons associated with each SMA/*t*BuSMA and PVP block with similar diffusion coefficients were observed, suggesting that chain extension had been successful (Figure 9). While **U***t*BuSMAS_25_-*b*-PVP_34_ exhibited a broad molecular weight distribution typical for this system, **S***t*BuSMAS_25_-*b*-PVP_13_ and **S**SMAS_25_-*b*-PVP_12_ had an additional low-molecular-weight shoulder at higher elution volumes than the macro-RAFT agent without a corresponding UV signal at 290 nm. DOSY NMR analysis showed protons characteristic of PVP (marked with a red asterisk on Figure 9) with larger diffusion coefficients than the macro-RAFT agent and associated block copolymer, suggesting that this low-molecular-weight material was a PVP homopolymer. It was hypothesized that aggregation of the macro-RAFT, such that the thiocarbonylthio group was shielded from the polymerization medium, could facilitate the homopolymerization of NVP. The aggregation of a representative macro-RAFT (**S**SMAS_25_) with varying pH, NVP concentration, and organic cosolvent content was assessed via DLS analysis (Figure S20).

DLS analysis of the **S**SMAS_25_ macro-RAFT agent, which had the largest proportion of the PVP homopolymer in the corresponding BCP sample, was conducted at varying concentrations of NVP (Figure S20). **S**SMAS_25_ was solubilized in PBS at 10% w/v, and increasing equivalents of NVP were added (9–45 equiv relative to **S**SMAS_25_) to mimic the polymerization medium for BCPs in Table 5. An aliquot of the solution was diluted in PBS and analyzed via DLS, showing that **S**SMAS_25_ formed large aggregates with a hydrodynamic diameter of ∼160 nm. The ω-chain end constituting the switchable thiocarbonylthio group and approximately 3 STY units is considerably hydrophobic and likely facilitates the association of ω-chain ends and subsequent aggregation of the macro-RAFT agent, enabling the partial homopolymerization of PVP. Upon addition of 500 equiv NVP to a solution of **S**SMAS_25_ (to mimic Table 3 block copolymerizations), 4 nm particles were observed, corresponding to **S**SMAS_25_ unimers as opposed to aggregates, suggesting that NVP has the capacity to cosolvate the **S**SMAS_25_ macro-CTA. Subsequently, ethanol was investigated as a potential cosolvent to facilitate chain extension experiments at low NVP concentrations. **S**SMAS_25_ had limited solubility in PBS with 40–50 v/v% ethanol but was soluble between 0 and 30 v/v% ethanol. All concentrations of ethanol yielded aggregates with variable hydrodynamic diameters, except for 30 v/v%, where a unimeric macro-RAFT agent was obtained. While the use of ethanol as a cosolvent would improve the solubility of the macro-RAFT agent, it is unlikely to vastly improve control over the RAFT-mediated polymerization of NVP, as the macro-RAFT agents have undergone alkaline hydrolysis prior to chain extension, inevitably yielding a significant proportion of dead chains.

## Conclusions

SMAnh and *t*BuSMAnh macro-RAFT agents were synthesized successfully using both universal 3,5-dimethylpyrazole dithiocarbamate and switchable *N*-(4-pyridinyl)-*N*-methyldithiocarbamate RAFT agents. Analysis of copolymerization kinetics for **U**SMAnh/**U***t*BuSMAnh and (**S–H**^**+**^)*t*BuSMAnh indicated that the RAFT-mediated polymerizations were well-controlled, as copolymers with excellent correlation between *M*_n_^theo^ and *M*_n_^SEC^ and low *Đ* could be synthesized. Additionally, the comonomer feed could be varied to allow for the preparation of macro-R-groups with either a STY/*t*BuSTY or MAnh monomer unit at the ω-chain end. **U**SMAnh-*b*-PVP was synthesized via a thermally initiated RAFT-mediated polymerization, confirmed via SEC and DOSY NMR analysis, but with significant thermolysis and hydrolysis of the dithiocarbamate Z-group due to the incorporation of PVP at the ω-chain end. ^1^H NMR spectroscopic analysis of **U**SMAnh-*b*-PVP indicated the presence of hydroxyl, aldehyde, or unsaturated functional groups at chain ends, while SEC analysis suggested that only 14% of thiocarbonylthio end-groups had been retained. To limit the thermolysis of the RAFT moiety, subsequent block copolymerizations were conducted at ambient temperature in an aqueous medium via redox initiated RAFT-mediated polymerization. The hydrophobic SMAnh/*t*BuSMAnh macro-RAFT agents were amenable to alkaline hydrolysis, yielding amphiphilic water-soluble copolymers (SMA/*t*BuSMA) but with significant loss of thiocarbonylthio end-groups, particularly for MAnh functional ω-chain ends. SMA/*t*BuSMA macro-RAFT mediated polymerizations yielded SMA-*b*-PVP and *t*BuSMA-*b*-PVP DHBCs with broad molecular weight distributions and poor retention of the thiocarbonylthio end-groups. In addition to the proportion of dead chain ends derived from alkaline hydrolysis of the macro-RAFT agents, the insertion of NVP at the ω-chain end exacerbated the hydrolysis of thiocarbonylthio end-groups during polymerization, the extent of which was governed by the type of RAFT agent used as well as the length of exposure to the aqueous medium. Both dithiocarbamates had improved hydrolytic stability compared to xanthates, with switchable *N*-(4-pyridinyl)-*N*-methyldithiocarbamate Z-groups exhibiting the highest stability. The well-controlled switchable RAFT-mediated synthesis of PVP could be achieved but with poor end-group fidelity, as the isolated polymer retained only 44% thiocarbonylthio functional ω-chain ends. Due to the susceptibility of the universal/switchable dithiocarbamate thiocarbonylthio groups toward thermolysis and hydrolysis, continued amelioration of this synthetic protocol is required, perhaps via the utilization of photoiniferter (PI)-RAFT polymerization that employs mild polymerization conditions. The PI-RAFT polymerization of NVP using universal macro-CTAs has been reported recently by Lian *et al*., but the prevalence of bimodality and significant broadening of block copolymer molecular weight distributions continue to highlight the challenges associated with the synthesis of poly(MAM-*b*-LAM) copolymers.^46^ Alternatively, the synthetic protocol for SMAnh-*b*-PVP could be ameliorated via synthesis in anhydrous organic solvent using a thermal initiator such as 2,2′-azobis(4-methoxy-2,4-dimethylvaleronitrile) (V-70), which decomposes at comparatively lower temperatures than AIBN.^36^

## Supporting Information Available

Additional experimental information, ^1^H NMR spectra, SEC eluograms, ATR-FTIR spectra, UV–vis spectra, DLS data, and computational data (PDF)

## ABBREVIATIONS

RAFT: reversible addition–fragmentation chain transfer
MAnh: maleic anhydride
STY: styrene
NVP: *N*-vinylpyrrolidone
MAc: maleic acid
SMAnh: poly(styrene-*alt*-maleic anhydride)
*t*BuSMAnh: poly(4-*tert*-butylstyrene-*alt*-maleic anhydride)
PVP: poly(*N*-vinylpyrrolidone)
BCP: block copolymer
DHBC: double hydrophilic block copolymer
