# Supplementary material for: An Exploration of the Universal and Switchable RAFT-Mediated Synthesis of Poly(styrene-alt-maleic acid)-b-poly(N-vinylpyrrolidone) Block Copolymers
Source: Macromolecules. 2025 Jan 13;58(2):1060–76. doi: 10.1021/acs.macromol.4c02741 (PMC11781032; doi:10.1021/acs.macromol.4c02741)
Supplement: Supplementary file 1 — ma4c02741_si_001.pdf [file ma4c02741_si_001.pdf]

Supplementary Information

An exploration of the universal and switchable RAFT-mediated synthesis of poly(styrene-*alt*-maleic acid)-*b*-poly(*N*-vinylpyrrolidone) block copolymers

Lauren E. Ball, Michael-Phillip Smith, Rueben Pfukwa, Bert Klumperman*.*

Synthesis of 1-Phenylethyl 3,5-dimethyl-1*H*-pyrazole-1-carbodithioate (U)

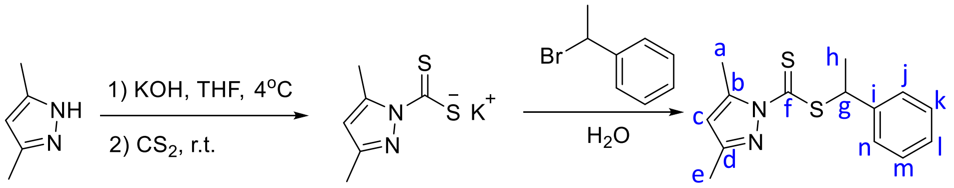

1-Phenylethyl 3,5-dimethyl-1*H*-pyrazole-1-carbodithioate was synthesized using a method adapted from literature.[1] A solution of KOH (4.0 g, 71 mmol) in THF (60 mL) was prepared and stirred at 4 °C. 3,5-Dimethylpyrazole (6.2 g, 64 mmol) in THF (27 mL) was added and the solution stirred for 5 min at 4 °C. CS_2 (6.4 g, 84 mmol) was added drop-wise while stirring at 4 °C, resulting in a colour change from colorless to bright yellow and subsequently the solution was allowed to stir at ambient temperature for 50 min yielding a dark orange suspension. The mixture was filtered and the solid potassium 3,5-dimethyl-1*H*-pyrazole-1-carbodithioate (KPC) washed with diethyl ether and then dried under vacuum. KPC was dried under vacuum and then KPC (7.4 g, 35 mmol) dispersed in acetone (65 mL) and DI water (65 mL). 1-Bromoethyl benzene (6.5 g, 35 mmol) was added dropwise while stirring at 4 °C and the solution stirred for a further 3 h at ambient temperature. The acetone was evaporated under reduced pressure and the aqueous dispersion further diluted with DI water (100 mL). The product was extracted using diethyl ether (3 × 100 mL), stirred over $MgSO_4$ overnight and then concentrated. **U** was recrystallized from MeOH to yield bright yellow crystals (6.8 g, 70% yield) and was analysed using UV-Vis spectroscopy, mass spectroscopy, ^{13}C and ^{1}H NMR spectroscopy and was determined to be 94% pure (determined *via* ^{1}H NMR spectroscopic analysis).

^{1}H NMR (400 MHz, $(CD_3)_2CO$) δ: 7.50–7.46 (m, 2H, H_k and H_m), 7.39–7.33 (m, 2H, H_j and H_n), 7.30–7.25 (m, 1H, H_l), 6.26 (s, 1H, H_c), 5.05–4.99 (q, J = 7.1 Hz, 1H, H_g), 2.66 (s, 3H, H_a), 2.18 (s, 3H, H_e), 1.77–1.74 (d, J = 7.2 Hz, 3H, H_h).

^{13}C NMR (100 MHz, $(CD_3)_2CO$) δ: 201.20 (C_f), 152.49 (C_b), 146.27 (C_d), 142.81 (C_i), 129.43 (C_k and C_m), 128.84 (C_j and C_n), 128.31 (C_l), 114.36 (C_c), 49.70 (C_g), 21.44 (C_h), 17.47 (C_a), 13.44 (C_e).

MS (ES+) m/z: 277.0838 ($M+H^+$, calculated), 277.0842 ($M+H^+$, experimental).

Synthesis of 2-Cyanopropan-2-yl *N*-methyl, *N*-(pyridin-4-yl) carbamodithioate (S)

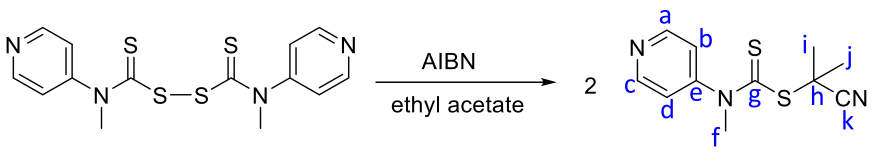

2-Cyanopropan-2-yl *N*-methyl, *N*-(pyridin-4-yl) carbamodithioate (**S**), a switchable RAFT agent first designed and utilized by Benaglia *et al.*, was synthesised according to a procedure adapted from literature.[2] *N*,*N*'-dimethyl *N*,*N*'-di(4-pyridinyl)thiuram disulfide (3.0 g, 8.2 mmol), AIBN (2.0 g, 12 mmol) and ethyl acetate (115 mL) were added to a 3-neck round bottom flask (RBF) fitted with a magnetic stirrer bar, rubber septa, condenser and bubbler. The reaction mixture was sparged for 45 min with dry argon and then the flask was immersed in an oil bath preheated to 80 °C. An additional portion of AIBN (0.68 g, 4.1 mmol) in ethyl acetate (12 mL) was sparged for 30 min and then added to the reaction mixture at 11 h. The reaction was stirred at 80 °C for an additional 5 h and then was quenched *via* removal from heat and exposure to atmospheric oxygen. Ethyl acetate was evaporated under reduced pressure and the crude product purified *via* column chromatography (50:50, ethyl acetate: hexane) and subsequently dried under high vacuum. **S** was obtained in reasonable yield (2.8 g, 67%) as a pale yellow/orange powder and was characterized *via* ^{13}C and ^{1}H NMR spectroscopy and determined to be 91% pure (determined *via* ^{1}H NMR spectroscopic analysis). Characterization data obtained for **S** corresponded well with that available in literature.[2]

^{1}H NMR (600 MHz, $(CD_3)_2CO$) δ: 8.76 (m, 2H, H_a and H_c), 7.47 (m, 2H, H_b and H_d), 3.70 (s, 3H, H_f), 1.81 (s, 6H, H_i and H_j).

^{13}C NMR (100 MHz, $(CD_3)_2CO$) δ: 192.81 (C_g), 152.65 (C_a and C_c), 152.20 (C_e), 122.75 (C_b and C_d), 121.78 (C_k), 44.85 (C_f), 43.21 (C_h), 27.38 (C_i and C_j).

Methylation of SMA/*t*BuSMA and corresponding block copolymers

A typical methylation procedure is described for the macro-CTAs and block copolymers. The copolymer (~50 mg) was dissolved in DI water (1 mL) and acidified with HCl (1.0 M, ~3 mL). This caused the precipitation of the macro-RAFT agent and the resulting suspension was lyophilized to ensure complete removal of water. The copolymer was dissolved (and in some cases suspended) in THF/MeOH (~1 mL, 20 v/v% MeOH), with the inclusion of 10 v/v% DMF for some samples to aid in solubilization of the copolymer. The methylating agent, (trimethylsilyl)diazomethane (2.0 M in hexane), was added drop-wise causing N_2 gas evolution. The methylating agent is bright yellow in colour and causes a corresponding bright yellow coloring of the polymer solution upon addition. Upon consumption of the methylating agent, the solution discolors to the initial colour of the copolymer solution and as such a persistent bright yellow colour, due to an excess of the methylating agent, is indicative of complete methylation having been achieved. The first treatment with methylating agent involved the addition of a 0.2 mL aliquot, and the polymer solution was subsequently stirred at ambient temperature for 3 h. If the solution had discolored, an additional 0.2 mL aliquot was added, but if the bright yellow coloring of the methylating agent had persisted, the solution was allowed to stir at ambient temperature for an additional 21 h. After 24 h, and with retention of the bright yellow coloring, the polymer solution was concentrated and dried under vacuum at 40 °C for 24 h. The extent of methylation was assessed *via* ATR-FTIR

spectroscopic analysis of the crude methylated copolymer and upon confirmation of successful methylation, the copolymer was analyzed *via* SEC.

Equation S1. STY and MAnh conversion calculation using vinylic proton integrals determined *via* ^{1}H NMR spectroscopic analysis of polymerization kinetic samples, using 1,3,5-trioxane as internal standard. Integral ranges are outlined below, where an average of the STY/*t*BuSTY integral is used in the equation.

$$\alpha = \left(1 - \frac{I_{tx}^{STY/MAnh}}{I_{t0}^{STY/MAnh}}\right) \times 100$$

$$\int STY1 = 5.824 - 5.784\, ppm, \int STY2 = 5.247 - 5.215\, ppm, \int MAnh = 7.323 - 7.319\, ppm$$

$$\int tBuSTY1 = 5.779 - 5.719\, ppm, \int STY2 = 5.195 - 5.153\, ppm, \int MAnh = 7.328 - 7.316\, ppm$$

Equation S2. M_n^{theo} calculation using monomer conversions calculated using the above equation.

$$M_n^{theo} = \frac{[STY] \times MW_{STY} \times \alpha_{STY}}{[RAFT]} + \frac{[MAnh] \times MW_{MAnh} \times \alpha_{MAnh}}{[RAFT]} + MW_{RAFT}$$

Where MW_{STY}, MW_{MAnh}, MW_{RAFT} are the molecular weights of STY, MAnh and the RAFT agent respectively. $A_{STY/MAnh}$ is the monomer conversion calculated using **Equation S1**. In cases where *t*BuSMAnh was synthesized, the molecular weight of *t*BuSTY was utilized in place of STY.

Equation S3. NVP monomer conversion calculation using vinylic proton integrals determined via ^{1}H NMR spectroscopic analysis of polymerization kinetic samples.

$$\alpha = \left(1 - \frac{I_{tf}^{6.989-6.929\, ppm}}{I_{t0}^{6.989-6.929\, ppm}}\right)$$

Equation S4. M_n^{theo} calculation for SMA-*b*-PVP copolymers

$$M_n^{theo} = \frac{[NVP] \times MW_{NVP} \times \alpha}{[macroCTA]} + MW_{macroCTA}$$

Where MW_{NVP} is the molecular weight of NVP, α is NVP conversion, and $MW_{macroCTA}$ is the molecular weight of the SMA macro-CTA.

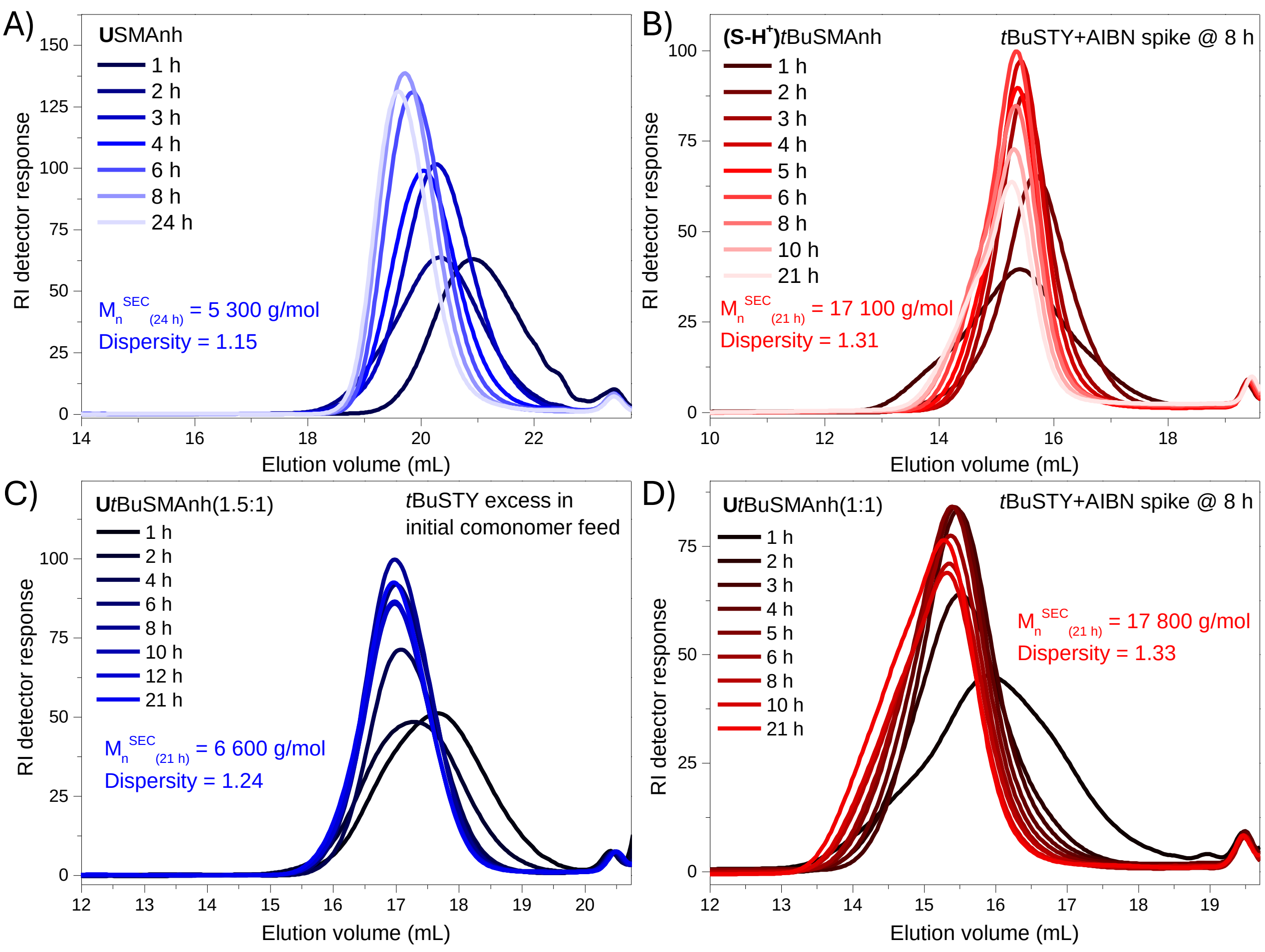

Figure S1. Kinetic samples, analyzed *via* SEC (THF, 5% AcOH) from SMAnh/*t*BuSMAnh copolymerizations (A) **USMAnh** (f_0^{STY}= 0.5), (B) **(S-H$^+$)*t*BuSMAnh*S*** (f_0^{tBuSTY}= 0.5 with *t*BuSTY spike at 8 h), (C) **U*t*BuSMAnh*S2*** (f_0^{STY}= 0.6) and (D) **U*t*BuSMAnh*S1*** (f_0^{tBuSTY}= 0.5 with *t*BuSTY spike at 8 h).

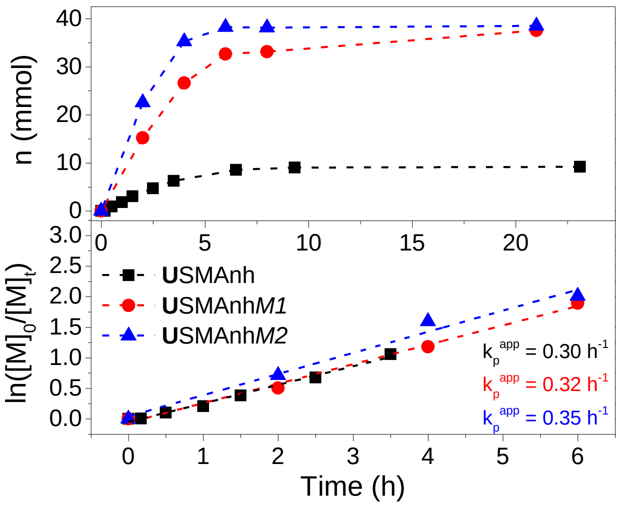

Figure S2. Kinetic analysis of the universal RAFT-mediated copolymerization of STY and MAnh, where the number of mol converted is determined *via* ^{1}H NMR spectroscopy to produce the mol converted *vs* time curve and the semilogarithmic plot, where **USMAnh** has a f_0^{MAnh} = 0.5, **USMAnh(M1)** has a f_0^{MAnh} = 0.5 with an additional 0.3 eq MAnh added at 8 h and **USMAnh(M2)** has a f_0^{MAnh} = 0.57.

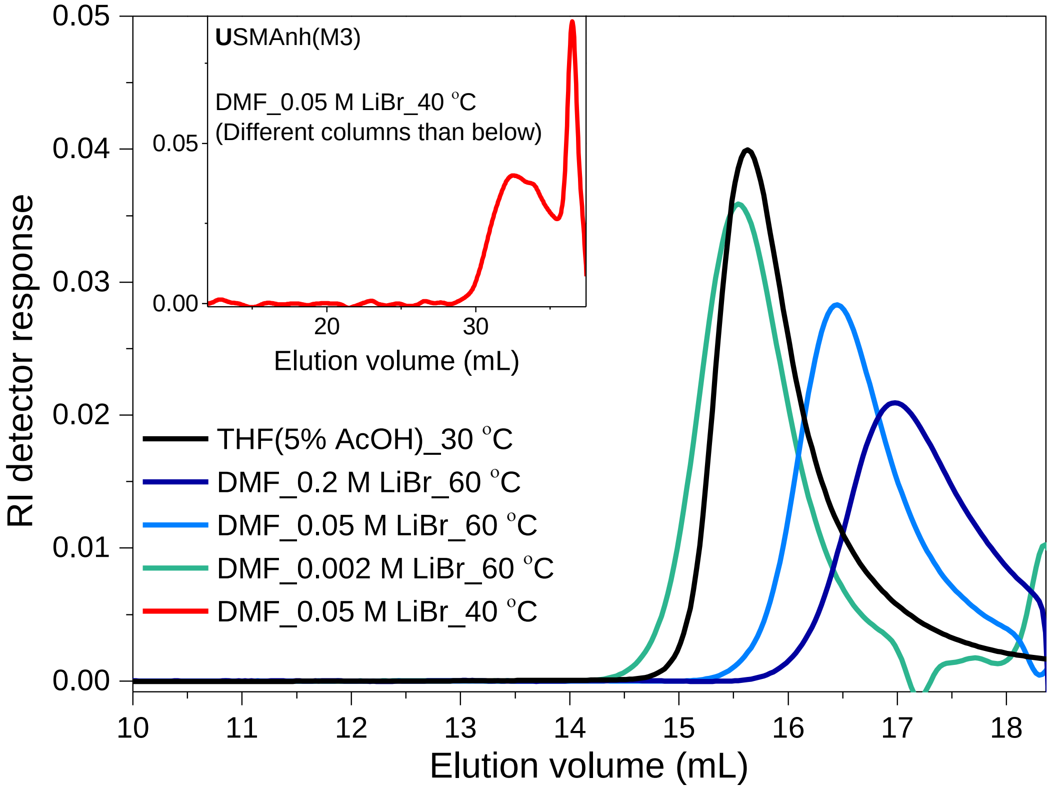

Figure S3. The effect of mobile phase composition in the SEC analysis of (**S-H**$^+$)SMAnh*S* copolymers. The same (**S-H**$^+$)SMAnh*S* copolymer was analyzed using three different SEC protocols, where DMF (0.2 M LiBr, 60 °C), DMF (0.05 M LiBr, 60 °C) and DMF (2 mM LiBr, 60 °C) are protocols using the same instrument components (as described in the main text) and PLgel Mixed-C columns thermostated at 60 °C. The DMF (0.05 M LiBr, 40 °C) protocol is also conducted using the same instrument components, but with PSS 10 µm GRAM columns thermostated at 40 °C (where the inset for samples **U**SMAnh*M3* has been included for reference). The THF (5% AcOH, 30 °C) protocol is conducted using an alternative instrument setup (as described in the main text), but also using PLgel Mixed-C columns.

The analysis of SMAnh type copolymers using THF (5% AcOH) as the eluent and PS calibration standards provides ideal molecular weight data, as the PS calibration standards have similar hydrodynamic volumes compared to SMAnh chains of similar molecular weight, under these solvency conditions. The added AcOH aids in protonation of and hydrolyzed MAnh moieties along the backbone, which tend to facilitate interactions with the column packing material and subsequently lower molecular weight tailing (underestimation of M_n and increase in *Đ*). As such, kinetic samples withdrawn from copolymerizations were analyzed using this SEC protocol. While this protocol is deal for SMAnh-type copolymers, it is incompatible with hydrolyzed SMAnh (SMA) and PVP making it an inappropriate choice for the analysis of SMA-*b*-PVP copolymers (in which case a mobile phase such as DMF is more appropriate).

Considering the analysis of the same (**S-H**$^+$)SMAnh*S* copolymer using the DMF protocols at 60 °C, the concentration of LiBr appears to have a profound effect on the hydrodynamic volume of the SMAnh chains (all analyzed at the same sample concentration of 2 mg/mL). A higher concentration of LiBr potentially creates a more compact hydrodynamic volume causing elution of the copolymer at comparatively higher elution

volumes than samples analyzed at lower LiBr concentration. This results in co-elution with the flow marker and underestimated M_n and increased $Đ$. As the concentration of LiBr is decreased from 0.2 M to 0.002 M the same copolymer elutes at increasingly lower elution volumes, where [LiBr] = 0.002 M yields a similar elution profile to the THF (5% AcOH) system. The calibration standards used for this protocol are PMMA standards, which incidentally overestimate the M_n of **(S-H$^+$)**SMAnh*S* at 0.002 M LiBr (M_n^{theo} = 5 600 g/mol, but M_n^{SEC}(PMMA) = 18 100 g/mol). Therefore, SMAnh calibration standards were synthesized using a trithiocarbonate RAFT agent, which allows for the synthesis of SMAnh with very narrow molecular weight distributions ($Đ$ ~ 1.10–1.20). The **(S-H$^+$)**SMAnh*S* copolymer was analyzed in DMF (0.002 M LiBr, 60 °C) and determined to have M_n^{SEC} = 5 900 g/mol and $Đ$ = 1.56, using the SMAnh calibration standards. While this protocol is useful for the analysis of SMAnh and SMAnh-*b*-PVP, block copolymer samples constituting SMA in its hydrolyzed form required methylation prior to analysis. At that point in the study, only the DMF (0.05 M LiBr, 40 °C) protocol was available, but fortunately methylated SMA did not co-elute with the flow marker as described above (the backbone of methylated SMA is far less rigid than SMAnh), and therefore provided reasonable molecular weight data despite being the least ideal SEC protocol described here for analysis of SMAnh-type copolymers.

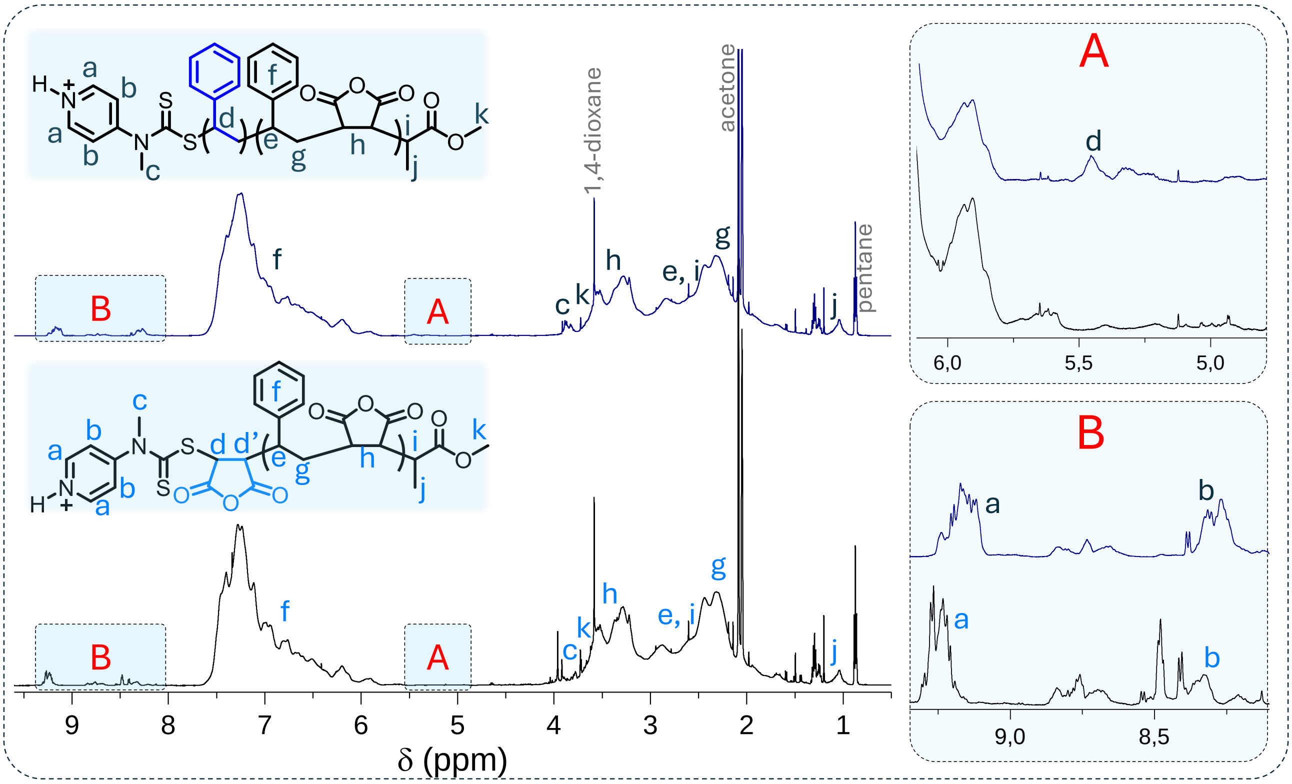

Figure S4. ^{1}H NMR spectroscopic analysis of **(S-H$^+$)**SMAnh(*S*/*M*) in $(CD_3)_2CO$.

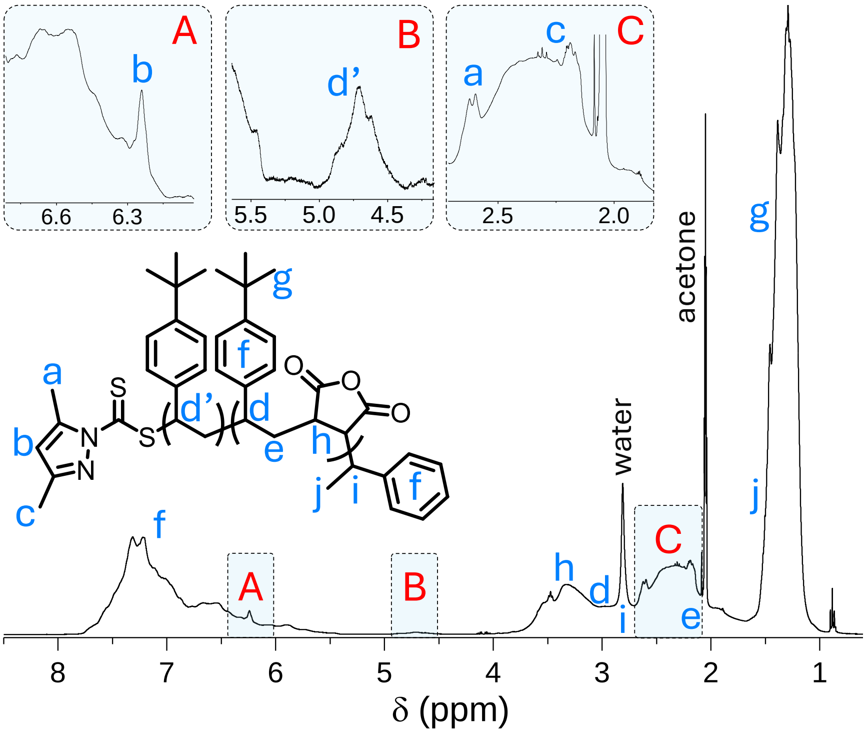

Figure S5. ^{1}H NMR spectroscopic analysis of **U***t*BuSMAnh in $(CD_3)_2CO$..

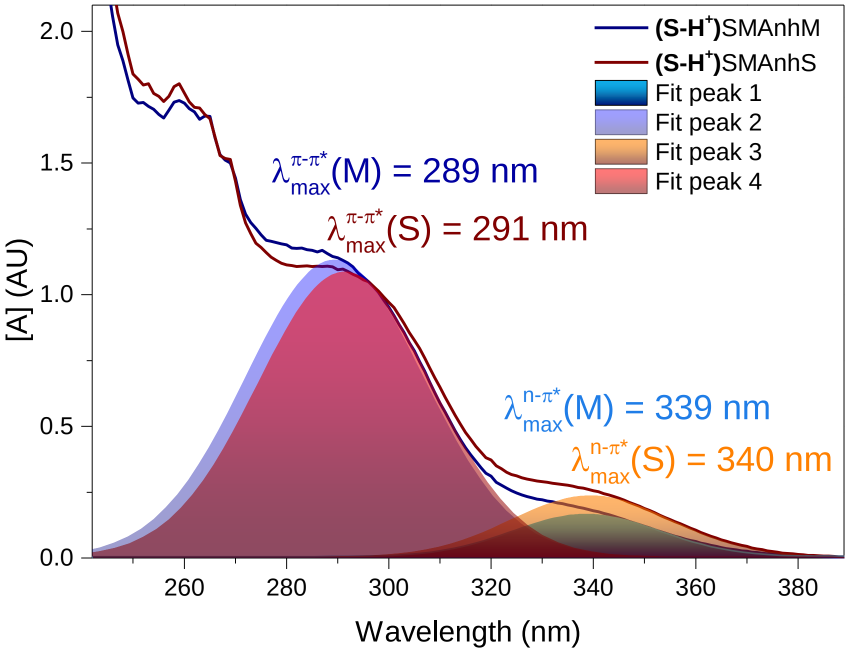

Figure S6. UV-Vis spectroscopic analysis of **(S-H$^+$)**SMAnh*M* *vs* **(S-H$^+$)**SMAnh*S* at 0.6 mg/mL in 1,4-dioxane.

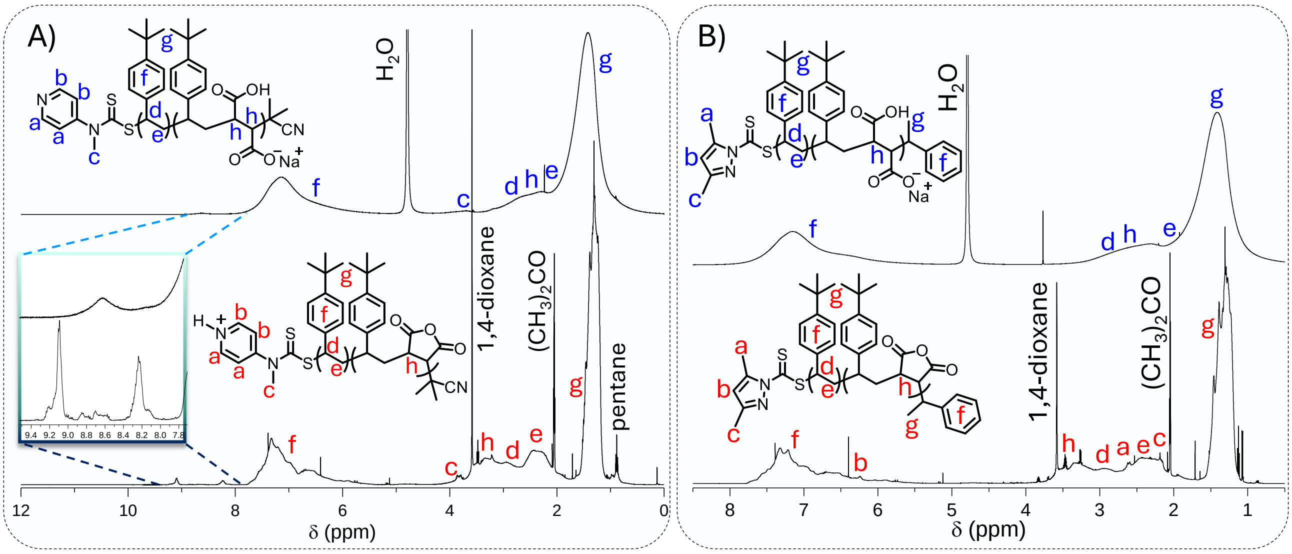

Figure S7. A) ^{1}H NMR spectroscopic analysis (600 MHz, Bruker) of (**S-H**$^{+}$)*t*BuSMAnh*S* (in $(CD_3)_2CO$) and corresponding deprotonated/hydrolyzed (Na_2CO_3-mediated) **S***t*BuSMA*S* (in D_2O) copolymer with inset indicating the upfield shift of the Z-group protons upon deprotonation. B) ^{1}H NMR spectroscopic analysis (600 MHz, Bruker) of **U***t*BuSMAnh*S* (in $(CD_3)_2CO$) and corresponding hydrolyzed (Na_2CO_3-mediated) **U***t*BuSMA*S* (in D_2O).

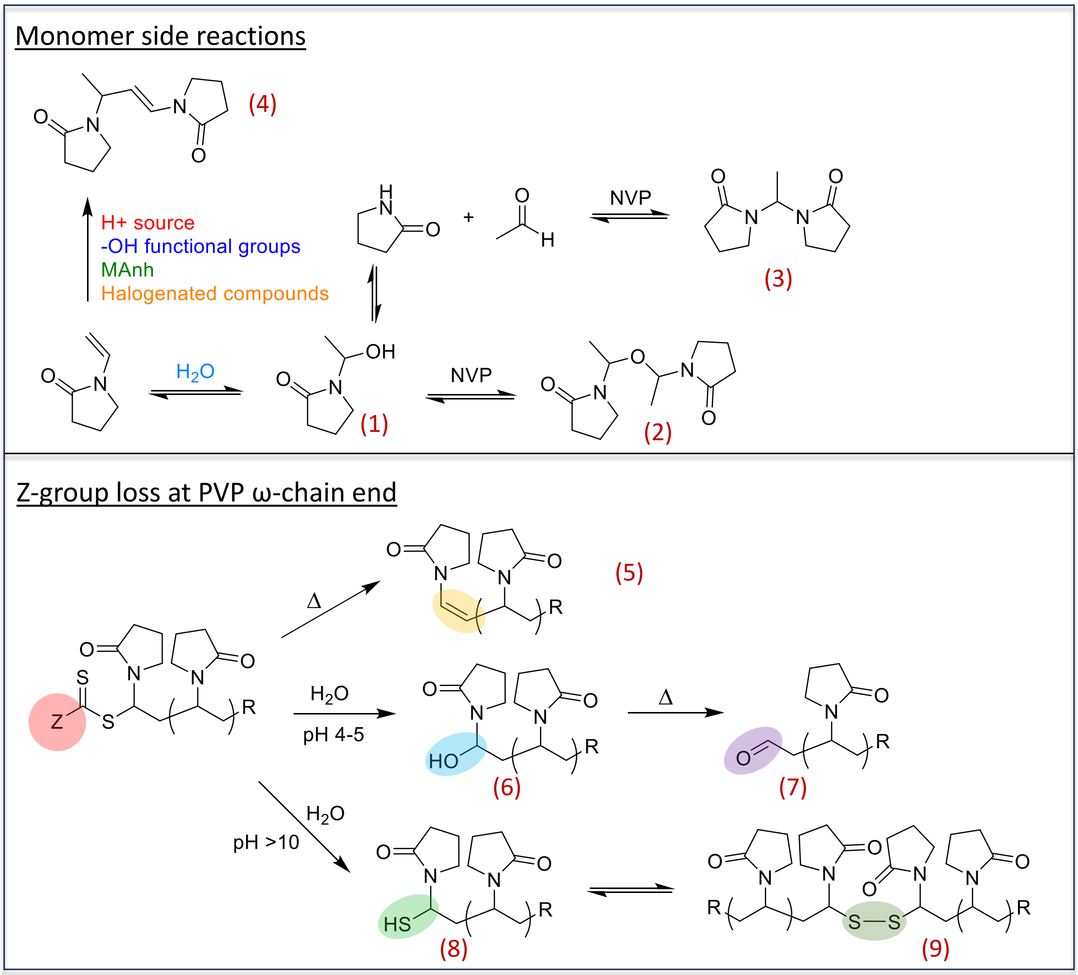

Figure S8. In addition to side reactions related to the RAFT polymerization of NVP (e.g. termination at high monomer conversion), the monomer can also be consumed in reactions related to the hydration of the vinyl bond or dimerization of NVP *via* catalysis with protic compounds, hydroxy functional molecules, MAnh or halogenated compounds (especially those employed in the synthesis of RAFT agents).[3] These side reactions have been summarized here, where firstly the hydration of the vinyl bond in NVP yields compound (**1)** which can either reversibly react with another molecule of NVP to produce the NVP hydration dimer (**2**) or it can further decompose into pyrrolidone and acetaldehyde.[4] The subsequent formation of 1,1-bis(*N*-pyrrolidonyl)-ethane (**3**) *via* the reaction of pyrrolidone and NVP is possible but is also a reversible reaction. Formation of the unsaturated NVP dimer (**4**) can be observed in the presence of protic hydrogens, but has also been shown to form in the presence of halogenated contaminants such as the alkyl halides used in the synthesis of RAFT agents.[3,5] Dissociation of alkyl bromides and subsequent formation of HBr have been shown to result in near quantitative unsaturated dimer (**4**) formation while use of alkyl chlorides have significantly less dissociation and therefore do not provide an abundant source of protic hydrogens, minimizing the prevalence of unsaturated dimer formation.[6] In a study by Pound *et al.*, the complete hydrolysis of the ethyl xanthogenate moieties at PVP chain ends was achieved *via* heating the polymer in distilled water at 40 °C for 16 h (yielding structure **6**).[7] The resulting hydroxy-functional end-groups (**6**) were transformed into aldehyde groups (~90% conversion) (**7**) *via* heating the polymer at 120 °C at 1 mbar for 20 h. If the hydrolysis was conducted at a *p*H higher than 10, it was possible to observe a significant proportion of thiol chain ends (**8**) with the associated

disulfide bridged counterparts (**9**). Additionally, significant elimination of RAFT Z-groups from polymer chains, yielding unsaturated chain ends (**5**), is possible and has a strong dependence on the relative structure of the monomer and RAFT moiety as well as the reaction temperature and the nature of the solvent utilized.[3,8,9] These degradative end-group reactions have been reported primarily within the context of xanthate functional PVP, but have not been demonstrated in great detail for dithiocarbamate Z-groups.[10]

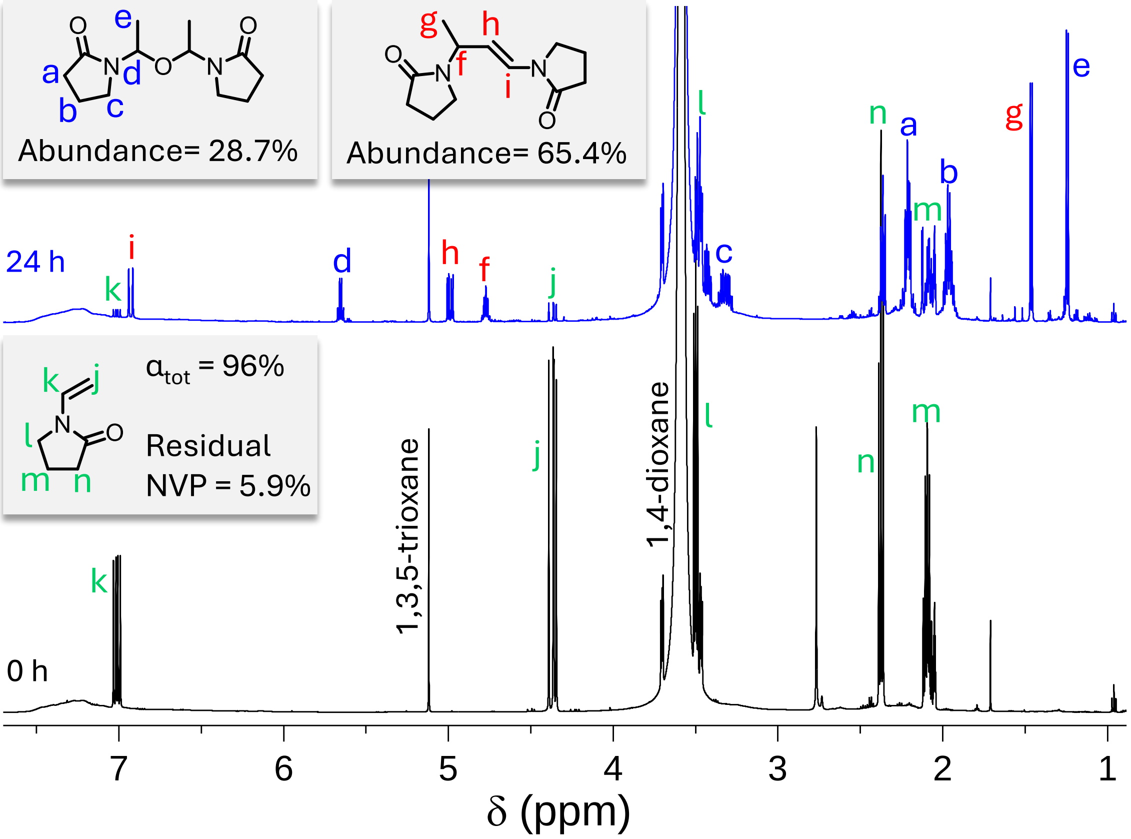

Figure S9. **USMAnh***S* (Table 2, entry 1 in main text) was isolated and subsequently chain extended with PVP in 1,4-dioxane at 60 °C (**USMAnh***S*-*b*-PVP, Table 2, entry 2). Kinetic samples were withdrawn at 0 h and 24 h and analyzed *via* ^{1}H NMR spectroscopy (600 MHz, Bruker), in $(CD_3)_2CO$). Protons characteristic of the NVP hydration dimer (labelled in blue), the unsaturated dimer (labelled in red) and residual unreacted NVP (labelled in green) are indicated with their associated abundance (calculated using the 1,3,5-trioxane internal standard).

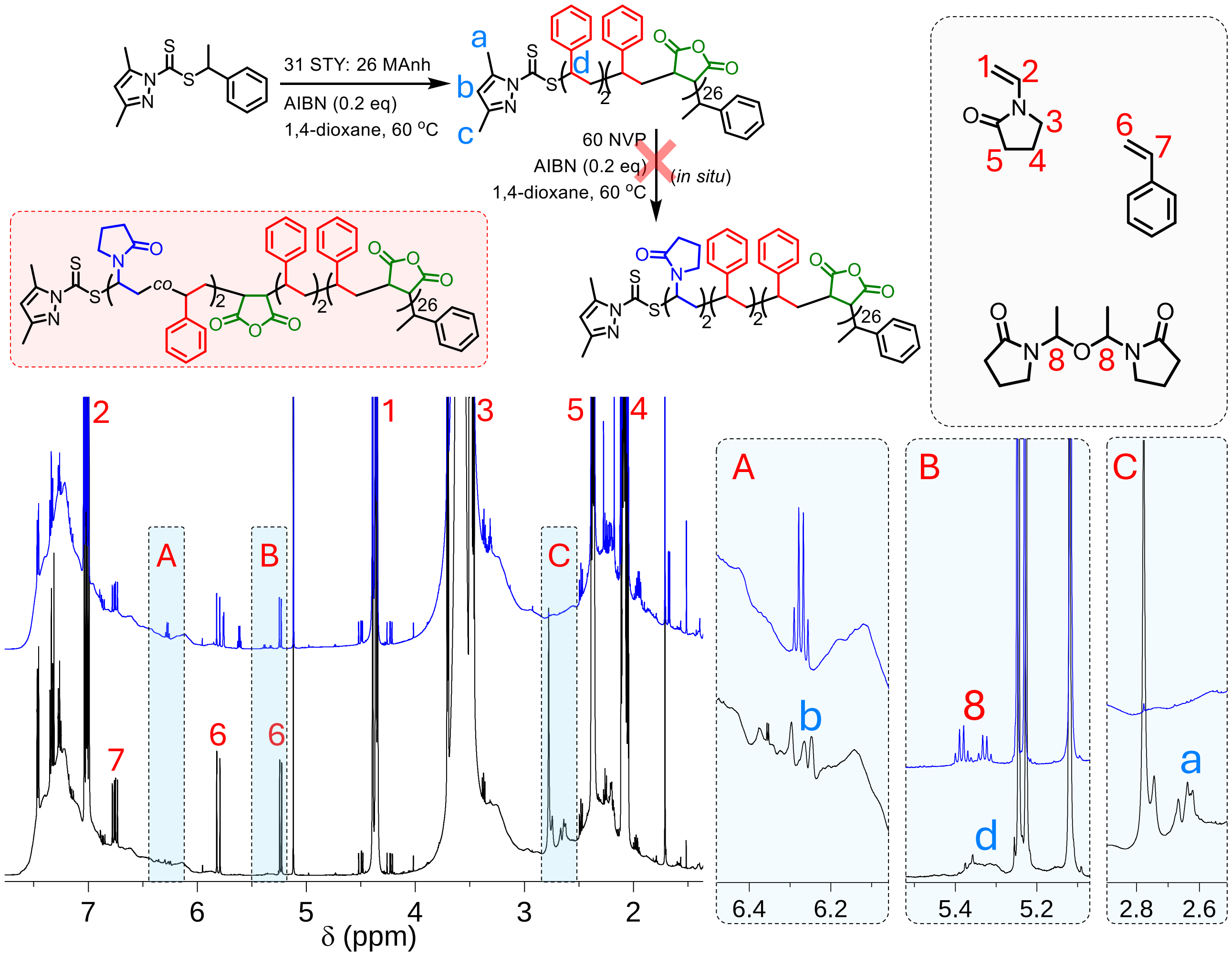

Figure S10. ^{1}H NMR spectroscopic analysis (600 MHz, Bruker) of the chain extension of **U**SMAnh*S** (Table 2, entry 3 in the main text) with PVP (**U**SMAnh*S*-*b*-PVP*, entry 4) in $(CD_3)_2CO$. Residual MAnh was consumed, corresponding to insertion of one MAnh unit, as well as an additional two STY units and two NVP units. Insets A, B and C indicate the loss of the Z-group as neither the 3,5-dimethyl pyrazole protons or terminal monomer unit methine proton could be observed in the 24 h spectrum (blue).

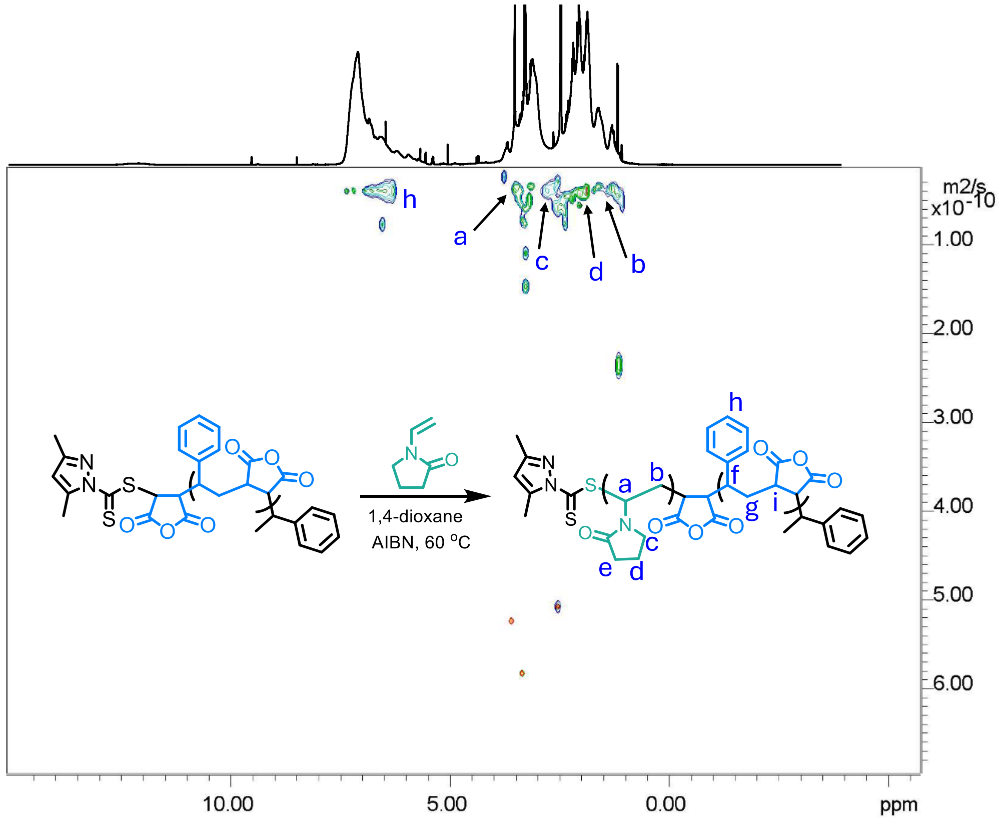

Figure S11. DOSY NMR spectrum for **U**SMAnh*M*-*b*-PVP in $(CD_3)_2SO$ (600 MHz, Bruker), processed using TopSpin and Dynamic Centre.

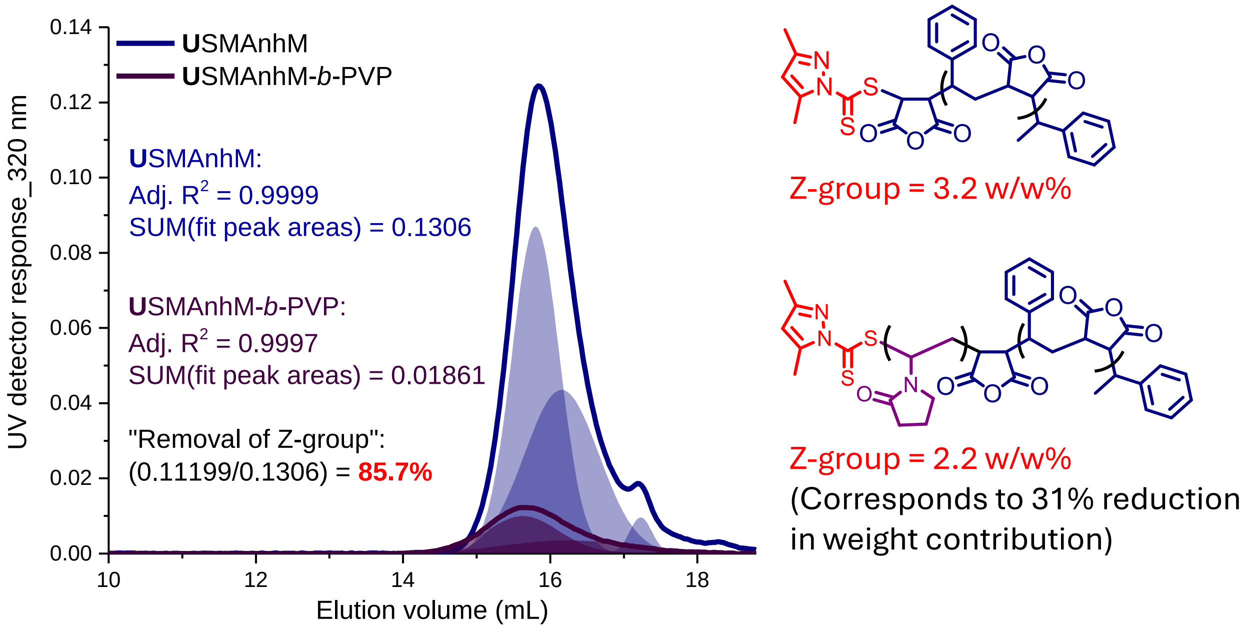

Figure S12. UV detector signals, obtained *via* SEC analysis of **U**SMAnhM* and corresponding **U**SMAnhM-*b*-PVP block copolymer, were deconvoluted using the Peak Analyzer (Fit Peaks Pro) function in Origin Pro 9. The fitting was conducted assuming a Gaussian fit for each peak, with the accuracy of the fit assessed using the adjusted R^2 values listed with the eluograms. Assuming the molar extinction coefficient does not change significantly upon insertion of NVP, the UV signal for the block copolymer should only decrease by approximately 31% as opposed to the observed 86%.

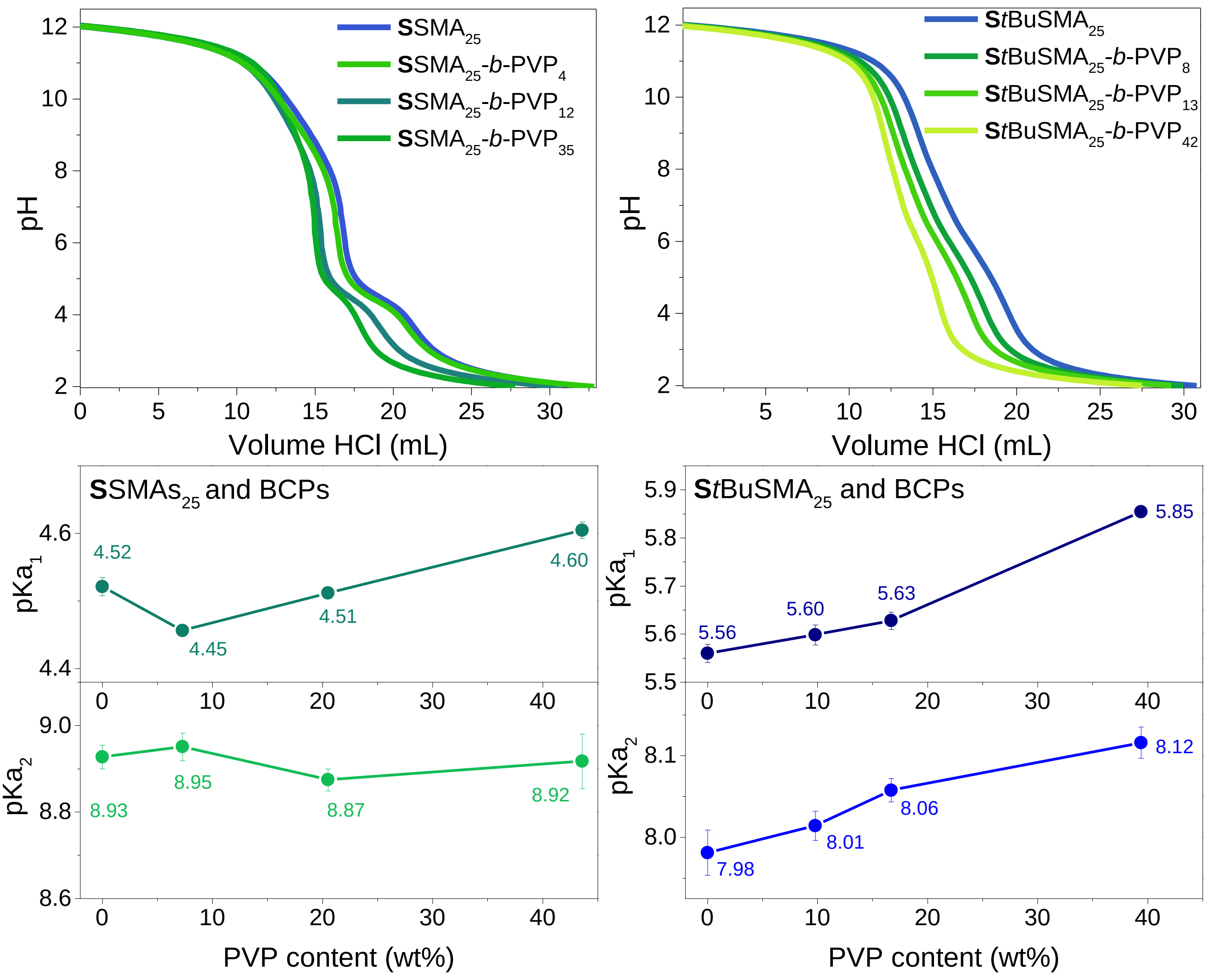

Figure S13. Acid titration of **S**SMA$_{25}$, **S***t*BuSMA$_{25}$ and their respective block copolymers for the determination of pK_{a1} and pK_{a2} for MAc units along the polymer backbone.

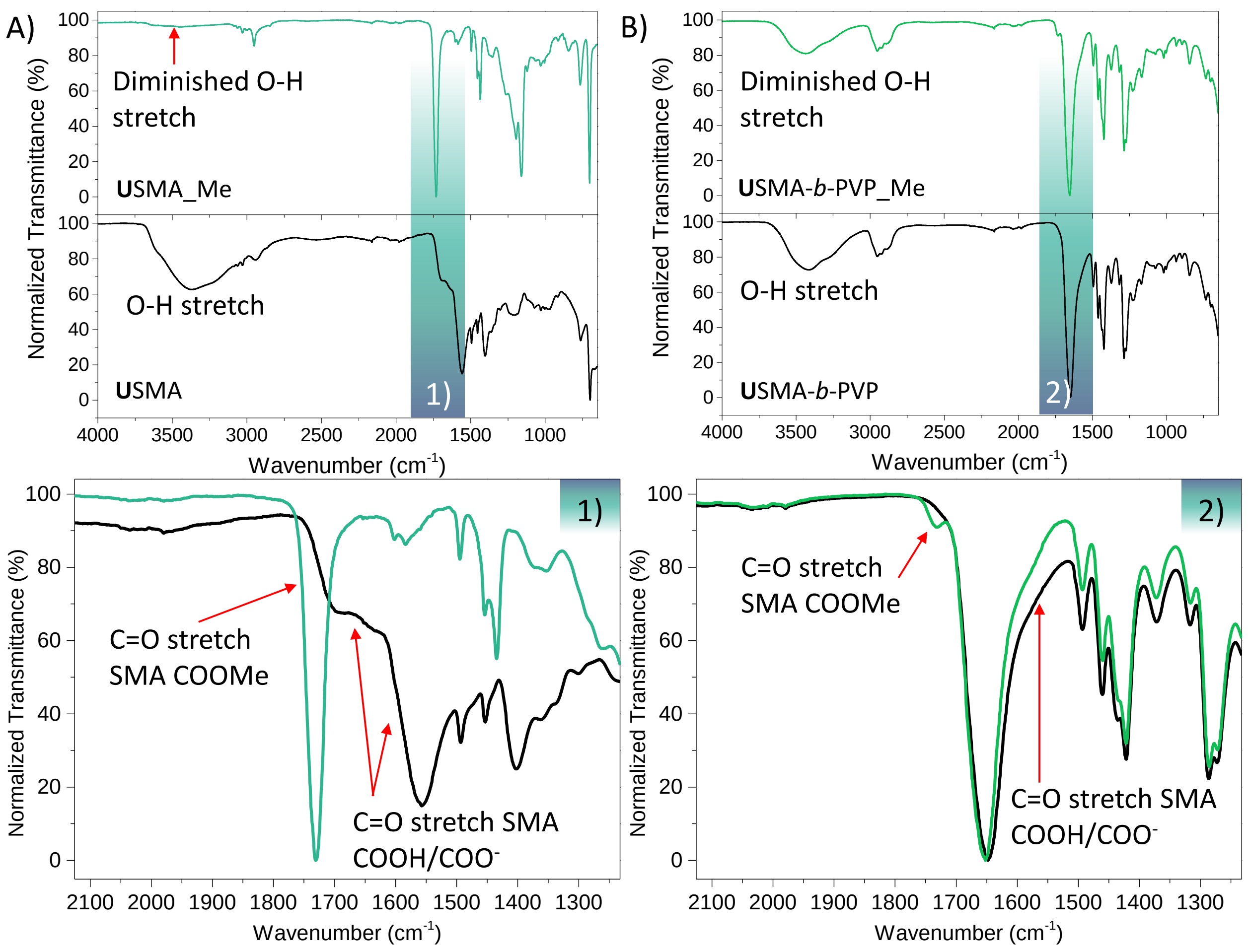

Figure S14. ATR-FTIR spectroscopic analysis for representative **U**SMA and **U**SMA-*b*-PVP copolymers that have undergone methylation using (trimethylsilyl)diazomethane. The disappearance of C=O stretches at 1650 and 1550 cm^{-1} (associated with MAc units along SMA backbone) and the appearance of a C=O stretch at ~1730 cm^{-1} (associated with conversion of MAc carboxylic acids to esters) indicate that methylation was successful. This transformation is also associated with the disappearance of the broad OH stretch at ~3500 cm^{-1}, which is diminished but not completely reduced for SMA-*b*-PVP samples due to water associated to the PVP backbone. The lactam functionalities along the PVP backbone exhibit a broad C=O stretch at ~1650 cm^{-1}, which makes analysis of the methylated samples somewhat challenging, but still diagnostic of successful methylation.

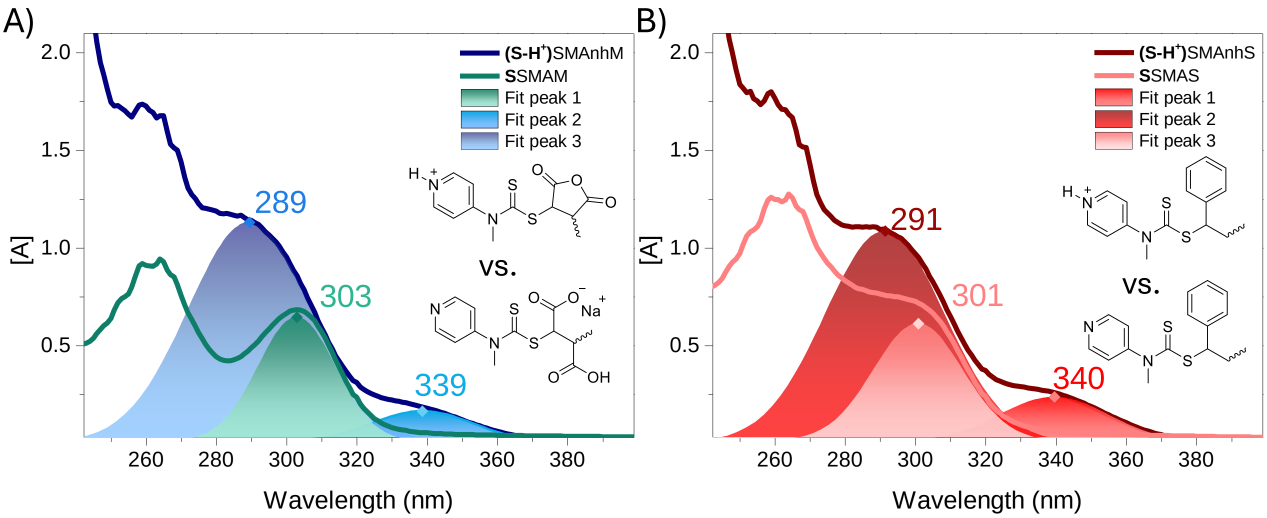

Figure S15. UV-Vis spectroscopic analysis of (**S-H**$^{+}$)SMAnhM/S in 1,4-dioxane and the corresponding **S**SMA(S/M) copolymer after Na_2CO_3-mediated deprotonation and hydrolysis. Quantitative analysis of the reduction in absorbance band areas (relating to the switchable Z-group) was not conducted, as the molar extinction coefficient is likely to have changed significantly with deprotonation of the pyridinyl moiety and the transformation of the MAnh-terminal macro-R-group into the MAc-terminal macro-R-group.

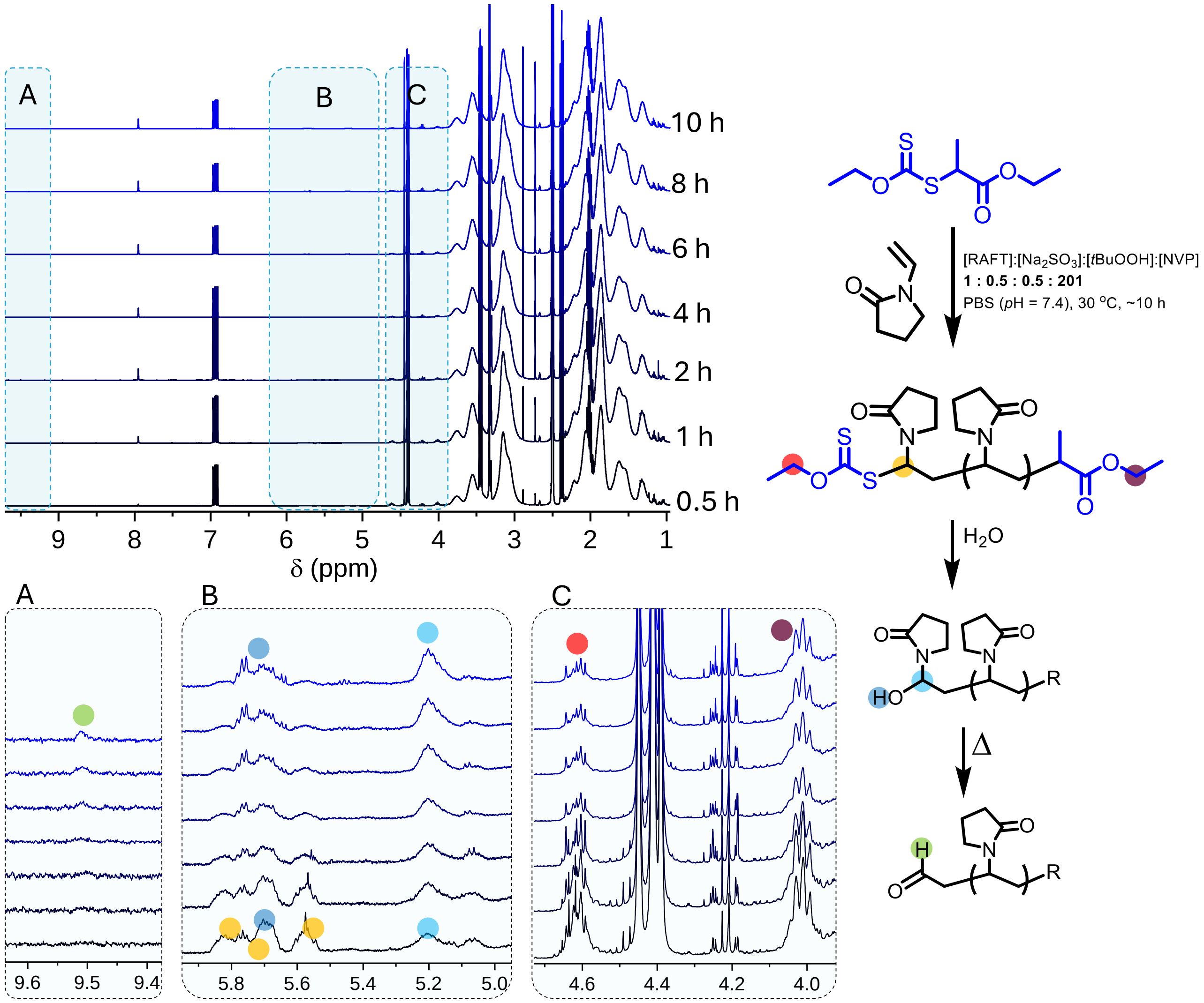

Figure S16. ^{1}H NMR spectroscopic analysis (600 MHz, Bruker) of lyophilized **X**PVP kinetic samples in $(CD_3)_2SO$.

Table S1. Calculation of end group loss using integrated ω-chain end protons for **X**PVP.

**Time (h)**	**I1** (5.872–5.523 ppm)	**I2** (5.283–5.110 ppm)	Corrected NVP CH (I1–I2= **I3**)	**I4** (9.523–9.498 ppm)	Total [sum(I2–I4)=**I5**]	**%Hyd.** (I2/I5)	**%EG loss** [(I2+I4)/I5]
**0.5**	0.84	0.20	0.64	0	0.84	23.8	**23.8**
**1**	0.77	0.19	0.58	0	0.77	24.7	**24.7**
**2**	0.91	0.39	0.52	0	0.91	42.9	**42.9**
**4**	0.89	0.48	0.41	0	0.89	53.9	**53.9**
**6**	0.78	0.47	0.31	0	0.78	60.3	**60.3**
**8**	0.90	0.61	0.29	0.02	0.92	66.3	**68.5**
**10**	0.82	0.58	0.24	0.02	0.84	69.0	**71.4**

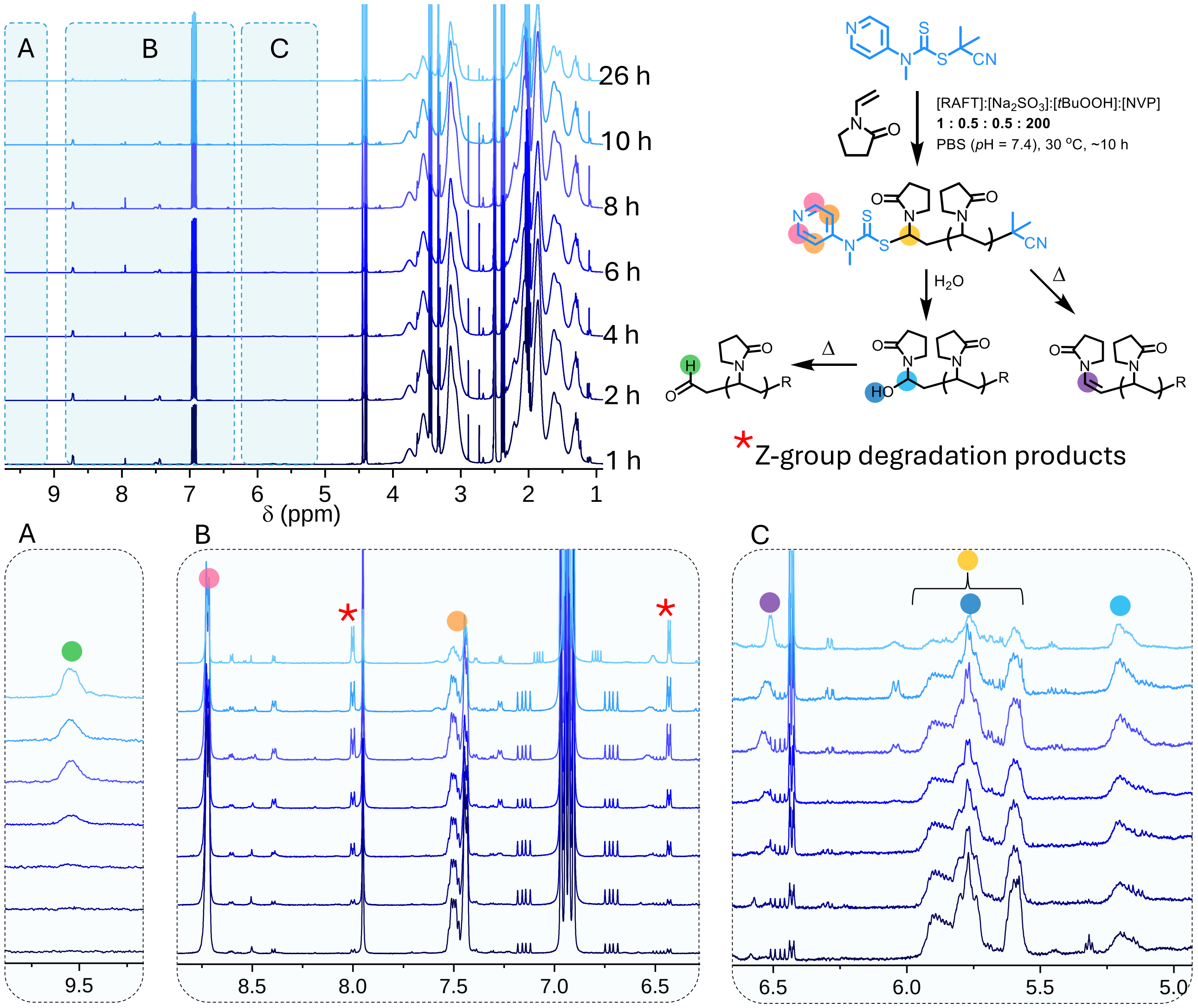

Figure S17. ^{1}H NMR spectroscopic analysis (600 MHz, Bruker) of lyophilized **SPVP** kinetic samples in $(CD_3)_2SO$.

Table S2. Calculation of end group loss using integrated ω-chain end protons for **SPVP**.

**Time (h)**	**I1** (5.948–5.548 ppm)	**I2** (5.248–5.103 ppm)	Corrected NVP CH (I1–I2= **I3**)	**I4** (9.530–9.481 ppm)	**I5** (6.538–6.485 ppm)	Total [sum(I2–I5)=**I6**]	**%Hyd.** (I2/I6)	**%EG loss** [(I2+I4+I5)/I6]
**1**	1.07	0.10	0.97	0	0	1.07	9.3	**9.3**
**2**	1.07	0.12	0.95	0	0	1.07	11.2	**11.2**
**4**	1.05	0.11	0.94	0.01	0	1.06	10.4	**11.3**
**6**	1.14	0.16	0.98	0.02	0.01	1.17	13.7	**16.2**
**8**	1.17	0.18	0.99	0.04	0.04	1.25	14.4	**20.8**
**10**	1.19	0.22	0.97	0.05	0.09	1.33	16.5	**27.1**
**26**	1.69	0.55	1.14	0.20	0.27	2.16	25.5	**47.2**
**62**	2.06	0.99	1.07	0.22	0.13	2.41	41.1	**55.6**

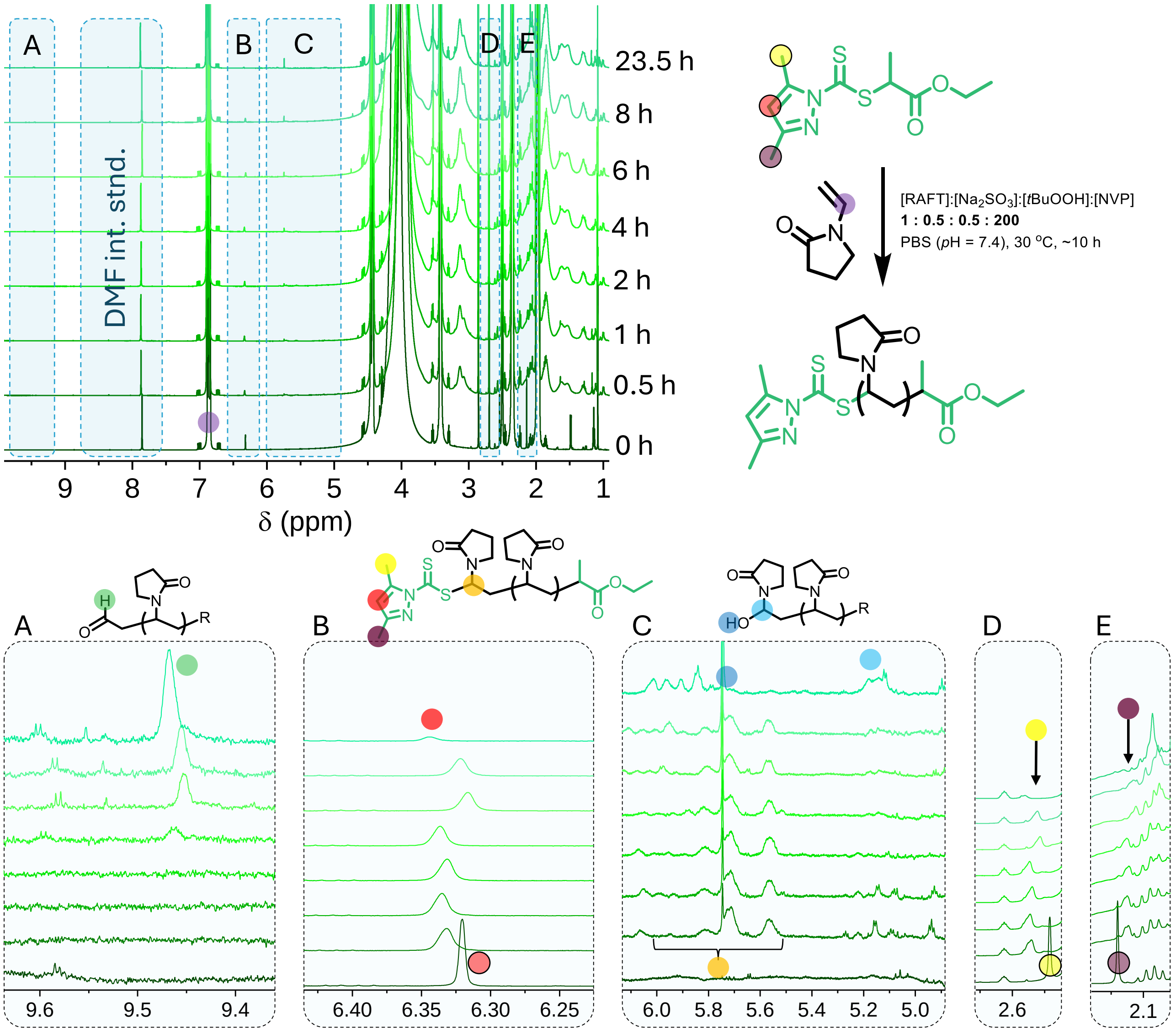

Figure S18. ^{1}H NMR spectroscopic analysis (600 MHz, Bruker) of crude **U**PVP kinetic samples in $(CD_3)_2SO$.

Table S3. Calculation of **U**PVP end group loss using integration of Z-group pyrazole proton *w.r.t* DMF internal standard.

Time (h)	I1 (6.304–6.355 ppm)	%EG loss [1-(**I1**tx/**I1**t0)]
**0**	0.26	**0**
**0.5**	0.25	**3.8**
**1**	0.24	**7.7**
**2**	0.22	**15.4**
**4**	0.19	**26.9**
**6**	0.18	**30.8**
**8**	0.16	**38.5**
**23.5**	0.04	**84.6**

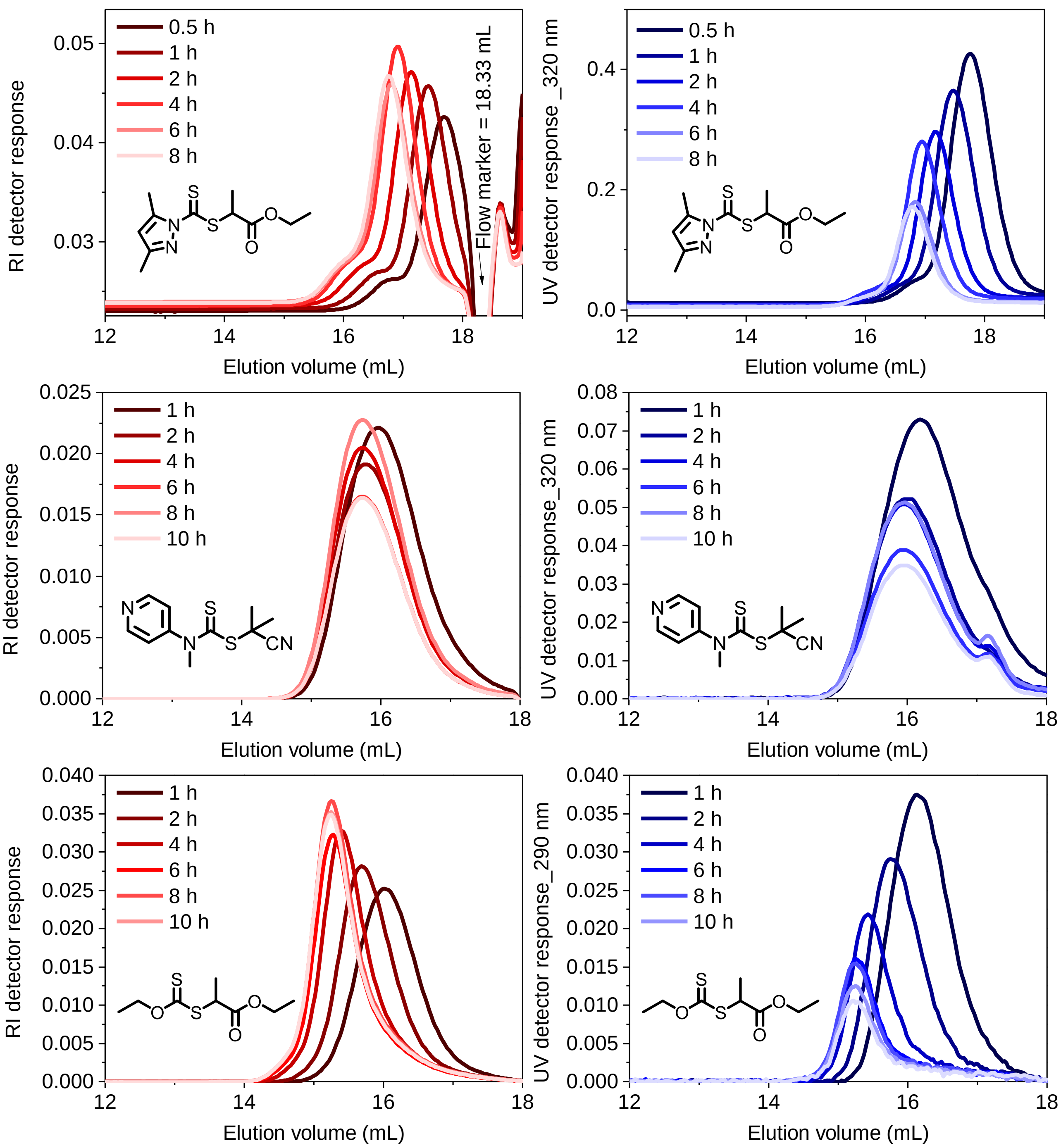

Figure S19. SEC analysis for **X**PVP, **U**PVP and **S**PVP lyophilized kinetic samples in DMF (2 mM LiBr, 60 °C).

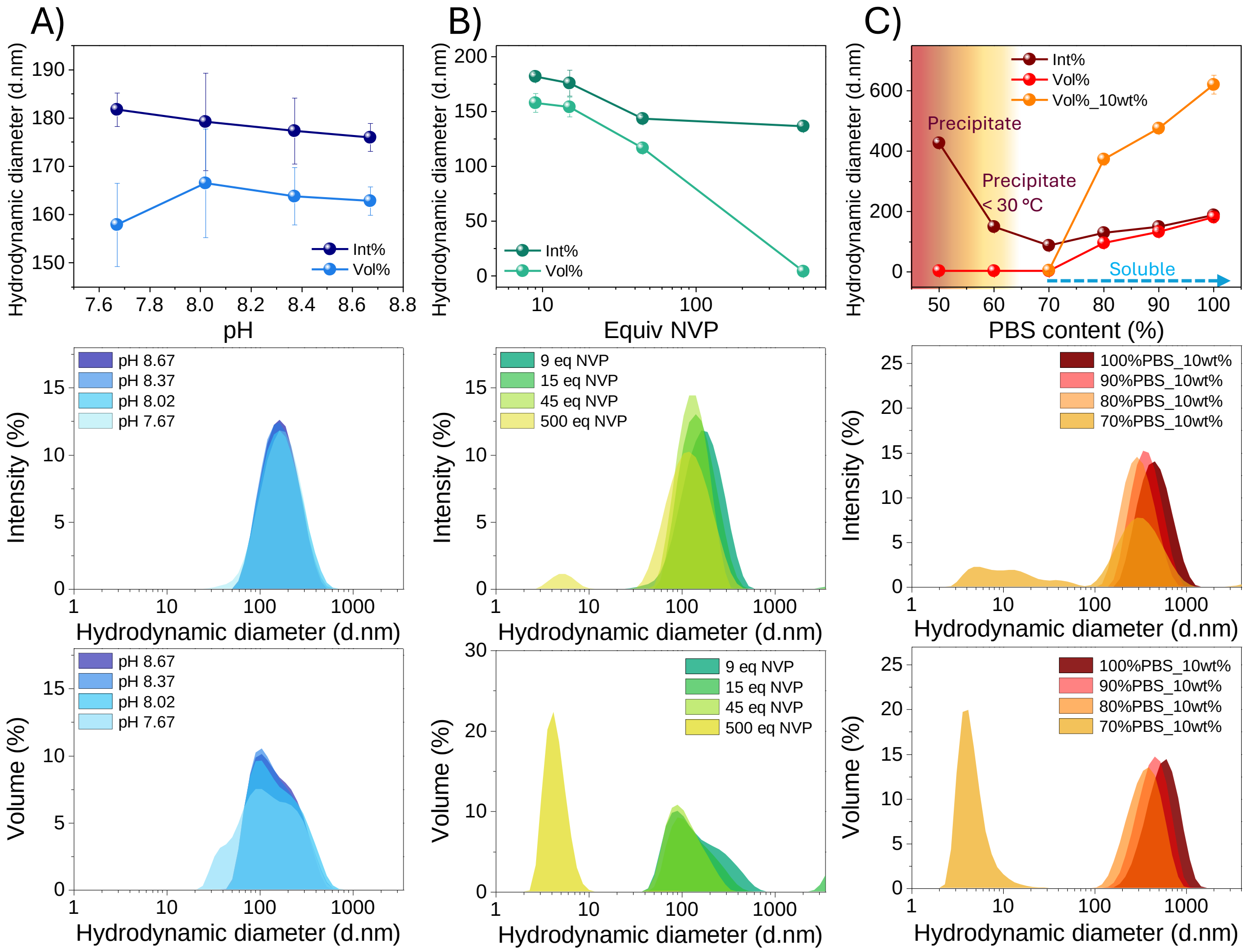

Figure S20. DLS analysis for the **SSMAS**$_{25}$ macro-RAFT agent in PBS (10 w/v% to mimic polymerization mixtures for **Table 3 & 5** in the main text). An aliquot of the concentrated solution is diluted in PBS (10 µL in 990 µL) for all analyses, except where stated otherwise. A) *p*H is varied to mimic titration of alkaline **SSMAS**$_{25}$ solutions to *p*H 7.6 prior to polymerization. B) Concentration of NVP varied to mimic concentrations employed in typical block copolymerization experiments. C) Ethanol content varied between 0–50% to assess applicability as co-solvent for **SSMAS**$_{25}$.

Computational software and methodology

Software

The Jaguar module of the Schrödinger Suite (2021-4),[11] was utilized for all calculations via the Maestro interface.[12] Basis sets were obtained from the Basis Set Exchange.[13-15]

Methodology

All geometries of chemical compounds were optimized using density functional theory at the B3LYP level,[16-19] using the 6-31+G* basis set.[20-23] The check_min=1 function of Jaguar was utilized to confirm if the optimized structures corresponded to a minimum energy where applicable. All minimization calculations were conducted with unrestricted spin. The water solvent environment was modelled using the conductor-like polarizable continuum model (CPCM) and compared to the optimized structure in the gas-phase.[24-26] Single point energy calculations were thereafter conducted at the M06-2X level,[27] utilizing the 6-311+G** basis set,[20, 28-31] in a similar manner as previously conducted in literature.[32]

Table S4. Computational data from the Gibbs free energy calculations conducted on **S**. The calculations were conducted at the B3LYP level, utilizing 6-31+G* basis set.

Reaction number	Gibbs free energy (kcal/mol)
**1**	0.159
**2**	-31.953
**3**	-1.881
**4**	-33.993
**5**	-6.068
**6**	-38.180
**7**	-4.319
**8**	-36.431

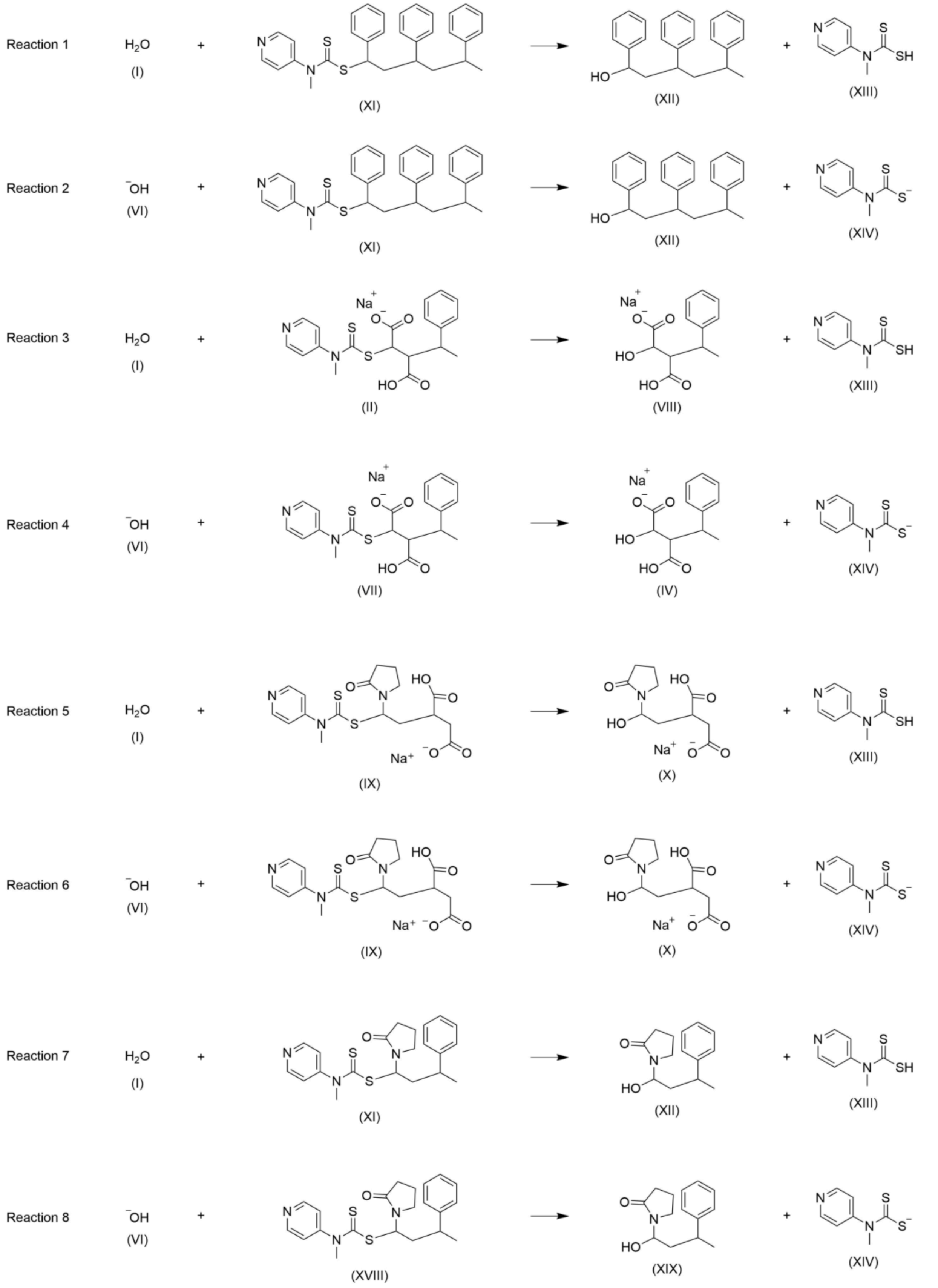

Figure S21. Structures investigated during the computational investigation. The computation was conducted with B3LYP, 6-31+G*.

Optimized atom coordinates and final energies

Compound I

Final energy = -76.430798 hartrees

H	0.00000	0.76820	0.52750
O	0.00000	0.00000	-0.06650
H	0.00000	-0.76820	0.52750

Compound II

Final energy = -2106.242801 hartrees

C	-2.73730	1.33100	0.64960
S	-1.05560	1.55530	0.06320
S	-3.35750	-0.12290	1.22890
C	-0.37600	-0.16690	-0.14200
C	1.16050	-0.02140	-0.03130
C	-0.84020	-0.81390	-1.43470
C	1.91070	-1.37390	0.01640
C	3.44860	-1.16770	0.03060
C	4.08410	-0.57740	-1.24980
C	5.60290	-0.39480	-1.03930
C	3.80880	-1.40130	-2.50280
C	-0.62380	-0.20590	-2.68280
C	-1.03430	-0.82990	-3.86270
C	-1.66580	-2.07870	-3.81780
C	-1.88210	-2.69470	-2.58250
C	-1.47470	-2.06440	-1.40120
C	1.49550	-2.23560	1.20480
C	3.29570	-0.78740	-3.65580
C	3.06600	-1.51840	-4.82710
C	3.34550	-2.88730	-4.86550
C	3.86010	-3.51400	-3.72460
C	4.09100	-2.77780	-2.55880
C	1.06450	-3.55650	1.00650
C	0.71440	-4.37490	2.08640
C	0.78900	-3.88320	3.39300
C	1.21920	-2.56900	3.60670
C	1.56870	-1.75640	2.52400
N	-3.47190	2.46620	0.60000
C	-2.92870	3.72410	0.15160
C	-4.87790	2.48210	1.04340
C	-3.20220	4.19010	-1.13590
C	-2.68290	5.43110	-1.50960
N	-1.93820	6.20090	-0.69890
C	-1.69380	5.73970	0.53790
C	-2.16730	4.51500	1.01420
H	-0.75430	-0.73780	0.70810
H	1.52990	0.56270	-0.88160
H	1.39630	0.55540	0.87130
H	1.65960	-1.93370	-0.89220
H	3.71370	-0.51530	0.87450
H	3.91850	-2.13750	0.24040
H	3.66290	0.42150	-1.41950
H	6.06890	0.06040	-1.92110
H	5.79930	0.25430	-0.17670
H	6.09500	-1.35850	-0.85700

H	-0.13980	0.76670	-2.73580
H	-0.86010	-0.34110	-4.81810
H	-1.98530	-2.56400	-4.73640
H	-2.37140	-3.66410	-2.53350
H	-1.65190	-2.54760	-0.44480
H	3.07050	0.27740	-3.63650
H	2.66550	-1.01770	-5.70550
H	3.16420	-3.46000	-5.77170
H	4.08240	-4.57850	-3.74210
H	4.49470	-3.28550	-1.68570
H	1.00170	-3.95000	-0.00580
H	0.38180	-5.39390	1.90490
H	0.51610	-4.51460	4.23470
H	1.28390	-2.17430	4.61810
H	1.90340	-0.73930	2.71580
H	-5.26880	3.48720	0.88470
H	-5.46280	1.76180	0.46550
H	-4.94320	2.22480	2.10500
H	-3.79470	3.60290	-1.83000
H	-2.87230	5.82230	-2.50650
H	-1.09380	6.37830	1.18270
H	-1.94600	4.18780	2.02520

<u>Compound III</u>

Final energy = -1005.280728 hartrees

O	-0.47820	2.28620	-1.52910
C	-0.69510	1.55820	-0.30500
C	-0.08880	0.15380	-0.46470
C	-2.17400	1.49600	0.04980
C	1.44920	0.06220	-0.31830
C	1.91890	-1.40750	-0.14380
C	1.45290	-2.14060	1.13690
C	1.98700	-3.58870	1.13110
C	1.83800	-1.41640	2.42220
C	-3.13460	1.11960	-0.90290
C	-4.48560	1.03270	-0.55450
C	-4.89650	1.31980	0.75320
C	-3.94760	1.69960	1.70730
C	-2.59620	1.78890	1.35430
C	2.22370	0.71120	-1.45800
C	0.85640	-1.07260	3.36460
C	1.19070	-0.42350	4.55910
C	2.52340	-0.10390	4.83440
C	3.51470	-0.44190	3.90530
C	3.17440	-1.09090	2.71470
C	3.11700	1.76250	-1.20180
C	3.86170	2.34920	-2.23160
C	3.72970	1.88640	-3.54390
C	2.84850	0.83270	-3.81360
C	2.10650	0.25210	-2.78130
H	-0.84360	3.17980	-1.41670
H	-0.17140	2.07350	0.51280
H	-0.54010	-0.47190	0.31140
H	-0.41510	-0.26360	-1.42700

H	1.71660	0.60110	0.60030
H	1.58680	-1.99650	-1.01030
H	3.01660	-1.41840	-0.17320
H	0.35790	-2.20710	1.11990
H	1.63050	-4.14190	2.00810
H	1.64870	-4.12050	0.23270
H	3.08370	-3.60830	1.14160
H	-2.82430	0.90000	-1.92160
H	-5.21880	0.74030	-1.30280
H	-5.94750	1.25230	1.02400
H	-4.25710	1.93180	2.72360
H	-1.86380	2.09070	2.10030
H	-0.18510	-1.31490	3.16100
H	0.40880	-0.16770	5.27070
H	2.78760	0.40180	5.75970
H	4.55520	-0.19950	4.10770
H	3.96130	-1.34230	2.00740
H	3.22780	2.13170	-0.18400
H	4.54290	3.16640	-2.00660
H	4.30570	2.33910	-4.34720
H	2.73840	0.46200	-4.83030
H	1.42480	-0.56330	-3.01390

<u>Compound IV</u>

Final energy = -1177.371292 hartrees

C	-0.63530	-1.27960	-0.04190
N	0.10150	-0.03980	-0.01660
C	1.44510	-0.00180	0.06220
C	-0.69140	1.20220	-0.07870
S	2.22500	-1.61650	0.10800
S	2.33840	1.42590	0.10760
C	-0.99870	-1.90930	1.14930
C	-1.73910	-3.09070	1.06650
N	-2.12270	-3.64720	-0.09320
C	-1.76890	-3.02210	-1.22740
C	-1.02870	-1.83830	-1.25890
H	-0.40110	1.78900	-0.95450
H	-1.74390	0.92800	-0.14910
H	-0.52430	1.79800	0.82300
H	3.47340	-1.11770	0.15820
H	-0.71400	-1.49620	2.11160
H	-2.03830	-3.60950	1.97410
H	-2.09030	-3.48740	-2.15610
H	-0.76540	-1.37020	-2.20190

<u>Compound V</u>

Final energy = -75.924017 hartrees

O	0.00000	0.00000	-0.05760
H	0.00000	0.00000	0.91430

<u>Compound VI</u>

Final energy = -1176.928203 hartrees

C	0.06300	-0.08580	-0.13160
N	1.50110	-0.06220	-0.13840

C	2.21930	0.07860	-1.29200
C	2.13620	-0.14540	1.18820
S	1.37000	0.11260	-2.80460
S	3.94220	0.20250	-1.18420
C	-0.62710	-1.29530	-0.02430
C	-2.01950	-1.26130	0.06600
N	-2.73980	-0.12750	0.06350
C	-2.05900	1.02860	-0.02730
C	-0.66980	1.10380	-0.12260
H	2.87510	-0.95020	1.20700
H	2.63770	0.79600	1.43690
H	1.36010	-0.34530	1.92720
H	-0.09480	-2.24140	-0.01440
H	-2.58240	-2.18910	0.14500
H	-2.65420	1.93930	-0.01950
H	-0.17020	2.06490	-0.19070

<u>Compound VII</u>

Final energy = -1942.296996 hartrees

C	-3.18890	1.51010	-0.71010
N	-2.35530	0.57860	0.00670
C	-1.03240	0.81830	0.16250
C	-3.02690	-0.63950	0.50200
S	-0.20280	-0.48970	1.04550
S	-0.27520	2.20900	-0.42940
C	1.53240	0.11480	1.17380
C	-3.82550	2.55180	-0.03570
C	-4.67580	3.38420	-0.76530
N	-4.91980	3.23340	-2.07770
C	-4.30530	2.21790	-2.70560
C	-3.43570	1.32890	-2.07080
C	2.52390	-1.08000	1.30580
C	1.77590	1.15300	2.32270
C	2.31470	-1.81610	2.62390
O	2.82220	1.84170	2.16290
O	3.30200	-1.58150	3.51080
O	1.36770	-2.54170	2.87700
O	0.97310	1.19720	3.28950
C	2.50850	-2.09760	0.12240
C	3.55830	-3.18710	0.32750
C	2.69160	-1.40280	-1.24090
C	3.16800	-4.53010	0.43990
C	4.11510	-5.54660	0.60610
C	5.47610	-5.23420	0.66470
C	5.87970	-3.89900	0.55370
C	4.93020	-2.88700	0.38600
H	-2.66310	-1.52610	-0.02750
H	-2.85620	-0.75890	1.57560
H	-4.09680	-0.53190	0.32530
H	1.74900	0.62190	0.23340
H	-3.66120	2.71440	1.02450
H	-5.18490	4.20830	-0.27170
H	-4.51720	2.10690	-3.76690
H	-2.96220	0.52330	-2.62340

H	3.50970	-0.60860	1.34490
H	3.10350	-2.07000	4.33520
H	1.52940	-2.58850	0.12010
H	2.75660	-2.15750	-2.03290
H	1.84690	-0.74820	-1.47730
H	3.60940	-0.80370	-1.27010
H	2.11120	-4.78450	0.39570
H	3.78750	-6.58020	0.69000
H	6.21490	-6.02130	0.79470
H	6.93560	-3.64380	0.59680
H	5.26790	-1.85720	0.29790

<u>Compound VIII</u>

Final energy = -841.343586 hartrees

O	0.91420	-1.66280	1.24480
C	1.69490	-0.52380	0.90530
C	2.77700	-0.87790	-0.15700
C	2.32410	0.05710	2.20980
C	3.58560	0.35400	-0.52880
O	3.01300	1.10370	2.11040
O	2.82240	1.34770	-1.03470
O	4.80200	0.43820	-0.46910
O	2.06510	-0.59230	3.26820
C	2.17740	-1.52050	-1.45920
C	1.71850	-2.96400	-1.27640
C	3.16370	-1.47030	-2.65100
C	0.42310	-3.34010	-1.65950
C	0.00130	-4.67140	-1.57710
C	0.87590	-5.65760	-1.11120
C	2.17490	-5.29760	-0.73560
C	2.59150	-3.96580	-0.82270
H	1.05300	-1.73510	2.21830
H	1.03490	0.24330	0.47870
H	3.49130	-1.57660	0.28940
H	3.40540	2.09210	-1.27750
H	1.29880	-0.92410	-1.73400
H	2.71490	-1.96190	-3.52090
H	3.40530	-0.44230	-2.94080
H	4.09950	-1.99110	-2.41700
H	-0.26800	-2.58070	-2.01960
H	-1.01050	-4.93580	-1.87530
H	0.55160	-6.69320	-1.04350
H	2.86720	-6.05500	-0.37540
H	3.60770	-3.71220	-0.52930

<u>Compound IX</u>

Final energy = -1996.689590 hartrees

C	-3.65530	1.66660	0.67410
N	-2.46640	0.85670	0.77370
C	-1.50780	0.92780	-0.17690
C	-2.37510	-0.01470	1.96400
S	-0.11790	-0.14250	0.18820
S	-1.62530	1.89880	-1.54700
C	1.14110	0.21150	-1.12590

C	-4.81130	1.15520	0.08580
C	-5.95490	1.95660	0.08090
N	-6.00340	3.18740	0.61570
C	-4.88130	3.65700	1.18530
C	-3.68510	2.93970	1.24330
N	1.83250	1.46770	-0.90720
C	2.12730	-0.97700	-1.17060
C	1.51790	-2.30870	-1.66290
C	2.29330	-2.90410	-2.85860
C	1.32780	-3.31030	-0.53360
C	3.73560	-3.36370	-2.55150
O	4.02170	-3.63200	-1.34870
O	4.51470	-3.46950	-3.54480
O	0.85830	-4.49640	-0.98580
O	1.48100	-3.09770	0.65800
C	2.29220	1.95480	0.40360
C	3.26270	3.09480	0.03570
C	2.78590	3.55650	-1.35100
C	2.10510	2.32600	-1.93260
O	1.83730	2.12500	-3.12420
H	-1.51620	0.26570	2.58160
H	-3.28700	0.11240	2.54680
H	-2.28950	-1.06430	1.66630
H	0.60940	0.30200	-2.07560
H	-4.82280	0.16540	-0.35910
H	-6.87330	1.59100	-0.37300
H	-4.93950	4.65330	1.61780
H	-2.80420	3.36250	1.71520
H	2.91320	-0.66480	-1.86660
H	2.60420	-1.10990	-0.19590
H	0.49700	-2.12150	-2.02950
H	1.75200	-3.77090	-3.25670
H	2.31640	-2.16010	-3.66240
H	0.71590	-5.08050	-0.21550
H	1.43630	2.31580	0.98690
H	2.77600	1.15230	0.96690
H	3.25210	3.89170	0.78360
H	4.28330	2.70360	-0.03170
H	2.03710	4.35750	-1.28470
H	3.58720	3.90330	-2.00860

<u>Compound X</u>

Final energy = -895.741469 hartrees

O	2.31150	0.71240	1.17030
C	1.62350	-0.46130	0.72650
N	0.21030	-0.40440	1.06340
C	1.86930	-0.71240	-0.76710
C	1.31080	-2.04620	-1.29690
C	1.48160	-2.15740	-2.83160
C	1.94090	-3.26990	-0.65930
C	0.54890	-1.23230	-3.65550
O	-0.55020	-0.88050	-3.14050
O	0.95810	-0.93830	-4.81770
O	3.27670	-3.16210	-0.48750

O	1.33330	-4.28690	-0.36270
C	-0.76330	0.45040	0.36110
C	-1.98410	0.44480	1.30430
C	-1.84030	-0.86730	2.09210
C	-0.34700	-1.16060	2.05270
O	0.26030	-1.96590	2.77210
H	2.17260	1.43180	0.53020
H	2.03960	-1.26930	1.33040
H	1.41780	0.09900	-1.35060
H	2.95000	-0.66780	-0.93480
H	0.24140	-2.11230	-1.08130
H	2.52170	-1.96390	-3.11470
H	1.25330	-3.18440	-3.15130
H	3.61100	-4.00080	-0.11240
H	-0.35630	1.45540	0.21480
H	-0.99600	0.02570	-0.62310
H	-1.92730	1.29840	1.98720
H	-2.92400	0.51440	0.75050
H	-2.19560	-0.81360	3.12410
H	-2.36130	-1.70230	1.60340

<u>Compound XI</u>

Final energy = -1851.008074 hartrees

C	-2.52310	1.26410	-0.61110
N	-1.56080	2.02230	-1.37320
C	-0.22720	1.88600	-1.20060
C	-2.11010	2.92440	-2.40260
S	0.18430	0.76470	0.14380
S	0.89500	2.70270	-2.15450
C	2.04030	0.82890	0.25110
C	-3.11270	1.81260	0.52890
C	-4.06830	1.05030	1.20450
N	-4.45300	-0.17560	0.81410
C	-3.87970	-0.68250	-0.28930
C	-2.91370	-0.00660	-1.03770
C	2.58190	-0.54830	0.68840
N	2.49640	1.89780	1.12650
C	2.37340	-1.71650	-0.30430
C	2.84780	-3.01740	0.33500
C	3.04430	-1.46820	-1.66930
C	1.92540	-4.02250	0.66420
C	2.33910	-5.21800	1.26300
C	3.69170	-5.42980	1.54470
C	4.62350	-4.43710	1.22110
C	4.20500	-3.24470	0.62300
C	2.23080	1.97820	2.46170
C	3.03660	3.13310	3.04070
C	3.51930	3.91470	1.80760
C	3.52300	2.86160	0.68430
O	1.47320	1.22720	3.09020
H	-1.72490	3.93680	-2.25530
H	-1.83000	2.57700	-3.40200
H	-3.19580	2.92430	-2.30420
H	2.37930	1.08430	-0.75340

H	-2.83370	2.79940	0.88460
H	-4.54460	1.44400	2.09950
H	-4.20710	-1.67430	-0.59260
H	-2.47890	-0.45740	-1.92410
H	3.65810	-0.40350	0.85230
H	2.15020	-0.81290	1.65860
H	1.29590	-1.82260	-0.47870
H	2.92070	-2.34080	-2.32100
H	2.59980	-0.60800	-2.18210
H	4.11870	-1.27650	-1.56080
H	0.86950	-3.86870	0.44970
H	1.60420	-5.98120	1.50780
H	4.01710	-6.35690	2.01010
H	5.67870	-4.59070	1.43480
H	4.94700	-2.48770	0.37990
H	2.42770	3.71860	3.73530
H	3.87250	2.70510	3.61070
H	2.80510	4.70910	1.56410
H	4.50360	4.37010	1.94300
H	4.49440	2.35540	0.59780
H	3.25330	3.27040	-0.29210

<u>Compound XII</u>

Final energy = -750.058218 hartrees

O	0.27950	0.09760	0.32710
C	1.64680	0.42260	0.53290
C	2.50370	-0.79990	0.90170
N	1.78510	1.47010	1.55570
C	2.45870	-1.95310	-0.12880
C	3.14890	-3.19190	0.43010
C	3.04000	-1.54620	-1.49770
C	2.40970	-4.35330	0.70310
C	3.02490	-5.49980	1.21910
C	4.39950	-5.50440	1.47350
C	5.14990	-4.35340	1.20690
C	4.53010	-3.21130	0.69110
C	1.09120	1.46290	2.71740
C	1.37310	2.75800	3.46240
C	2.59620	3.34660	2.73490
C	2.53670	2.71400	1.32860
O	0.31890	0.55220	3.07650
H	-0.08000	-0.14090	1.20610
H	1.98880	0.86290	-0.40620
H	3.53880	-0.45530	1.03040
H	2.16600	-1.17110	1.87690
H	1.40470	-2.21440	-0.27880
H	3.02020	-2.39430	-2.19180
H	2.46120	-0.73410	-1.95180
H	4.07960	-1.20760	-1.40820
H	1.33890	-4.36130	0.50900
H	2.42940	-6.38710	1.42080
H	4.88160	-6.39310	1.87380
H	6.22040	-4.34450	1.40030
H	5.13330	-2.32860	0.49020

H	0.48630	3.39930	3.37140	H	3.51750	3.03540	3.23920
H	1.53480	2.56660	4.52700	H	3.52790	2.49160	0.92070
H	2.58560	4.43840	2.68990	H	1.99980	3.34620	0.60860

References

1. J. Gardiner, I. Martinez-Botella, J. Tsanaktsidis and G. Moad, *Polym. Chem.*, 2016, **7**, 481–492.
2. M. Benaglia, J. Chiefari, Y. K. Chong, G. Moad, E. Rizzardo and S. H. Thang, *J. Am. Chem. Soc.*, 2009, **131**, 6914–6915.
3. G. Pound, Z. Eksteen, R. Pfukwa, J. M. Mckenzie, R. F. M. Lange and B. Klumperman, *J. Polym. Sci. Part A: Polym. Chem.*, 2008, **46**, 6575–6593.
4. E. Senogles and R. A. Thomas, *J. Chem. Soc., Perkin Trans.*, 1980, **2**, 825–828.
5. A. Madl and S. Spange, *Macromolecules*, 2000, **33**, 5325–5335.
6. C. F. Huang, R. Nicolay, Y. Kwak, F. C. Chang and K. Matyjaszewski, *Macromolecules*, 2009, **42**, 8198–8210.
7. G. Pound, J. M. McKenzie, R. F. M. Lange and B. Klumperman, *Chem. Commun.*, 2008, **27**, 3193–3195.
8. T. M. Legge, A. T. Slark and S. Perrier, *J. Polym. Sci. Part A: Polym. Chem.*, 2006, **44**, 6980–6987.
9. M. Destarac, I. Blidi, O. Coutelier, A. Guinaudeau, S. Mazières, E. Van Gramberen and J. Wilson, in Controlled Radical Polymerization: Mechanisms and Techniques, American Chemical Society, 2012, vol. Chapter 17, pp. 259–275.
10. D. J. Keddie, C. Guerrero-Sanchez, G. Moad, E. Rizzardo and S. H. Thang, *Macromolecules*, 2011, **44**, 6738–6745.
11. A. D. Bochevarov, E. Harder, T. F. Hughes, J. R. Greenwood, D. A. Braden, D. M. Philipp, D. Rinaldo, M. D. Halls, J. Zhang and R. A. Friesner, International Journal of Quantum Chemistry, 2013, 113, 2110-2142.
12. Schrödinger, 2021-4.
13. D. Feller, *J. Comput. Chem.*, 1996, **17**, 1571-1586.
14. B. P. Pritchard, D. Altarawy, B. Didier, T. D. Gibsom and T. L. Windus, *J. Chem. Inf. Model.*, 2019, **59**, 4814-4820.

15. K. L. Schuchardt, B. T. Didier, T. Elsethagen, L. Sun, V. Gurumoorthi, J. Chase, J. Li and T. L. Windus, *J. Chem. Inf. Model.*, 2007, **47**, 1045-1052.

16. S. H. Vosko, L. Wilk and M. Nusair, *Can. J. Phys.*, 1980, **58**, 12.

17. P. J. Stephens, F. J. Devlin, C. F. Chabalowski and M. J. Frisch, *J. Phys. Chem.*, 1994, **98**, 5.

18. A. D. Becke, *J. Chem. Phys.*, 1993, **98**, 5648-5652.

19. C. Lee, W. Yang and R. G. Parr, *Phys. Rev. B*, 1988, **37**, 785-789.

20. T. Clark, J. Chandrasekhar, G. W. Spitznagel and P. V. R. Schleyer, *J. Comput. Chem.*, 1983, **4**, 294-301.

21. R. Ditchfield, W. J. Hehre and J. A. Pople, *J. Chem. Phys.*, 1971, **54**, 724-728.

22. W. J. Hehre, R. Ditchfield and J. A. Pople, *J. Chem. Phys.*, 1972, **56**, 2257-2261.

23. P. C. Hariharan and J. A. Pople, *Theor. Chim. Acta*, 1973, **28**, 213-222.

24. M. Cossi, N. Rega, G. Scalmani and V. Barone, *J. Comput. Chem.*, 2003, **24**, 669-681.

25. A. Klamt and G. Schüürmann, *J. Chem. Soc., Perkin Trans.*, 1993, **2**, 799-805.

26. M. Cossi and V. Barone, *J. Phys. Chem.*, 1998, **102**, 7.

27. Y. Zhao and D. G. Truhlar, *Theor. Chem. Acc.*, 2007, **120**, 215-241.

28. M. M. Francl, W. J. Pietro, W. J. Hehre, J. S. Binkley, M. S. Gordon, D. J. DeFrees and J. A. Pople, *J. Chem. Phys.*, 1982, **77**, 3654-3665.

29. R. Krishnan, J. S. Binkley, R. Seeger and J. A. Pople, *J. Chem. Phys.*, 1980, **72**, 650-654.

30. A. D. McLean and G. S. Chandler, *J. Chem. Phys.*, 1980, **72**, 5639-5648.

31. G. W. Spitznagel, T. Clark, P. v. R. Schleyer and W. J. Hehre, *J. Comput. Chem.*, 1987, **8**, 1109-1116.

32. G. B. Desmet, R. D'Hooge D, M. K. Sabbe, M. F. Reyniers and G. B. Marin, *J. Org. Chem.*, 2016, **81**, 11626-11634.
